# Exploratory phase II trial of an anti-PD-1 antibody camrelizumab combined with a VEGFR-2 inhibitor apatinib and chemotherapy as a neoadjuvant therapy for triple-negative breast cancer (NeoPanDa03): efficacy, safety and biomarker analysis

**DOI:** 10.1038/s41392-025-02337-1

**Published:** 2025-07-21

**Authors:** Xiaoxiao Liu, Chunying Zhuang, Lei Liu, Ling Xiong, Xin Xie, Ping He, Juanjuan Li, Bing Wei, Xi Yan, Tinglun Tian, Xiaorong Zhong, Jie Chen, Yan Cheng, Dan Zheng, Peng Cheng, Tianlin Sun, Weiwei Li, Changbin Zhu, Shuaitong Chen, Chao Fang, Jun Fu, Shibao Li, Jing Jing, Ting Luo

**Affiliations:** 1 Institute of Breast Health Medicine, Breast Center, Department of Medical Oncology, Cancer Center, West China Hospital, Sichuan University, Chengdu, Sichuan 610041 China; 2 Department of Radiation Oncology, Cancer Center, Affiliated Hospital of Xuzhou Medical University; Cancer Institute, Xuzhou Medical University, Xuzhou, Jiangsu 221000 China; 3 Institute of Breast Health Medicine, West China Hospital, Sichuan University, Chengdu, Sichuan 610041 China; 4 Department of physiology, School of Basic Medical Sciences, Xuzhou Medical University, Xuzhou, Jiangsu 221000 China; 5 Department of Laboratory Medicine, Affiliated Hospital of Xuzhou Medical University, Xuzhou, Jiangsu 221000 China; 6 Department of Pathology, West China Hospital, Sichuan University, Chengdu, Sichuan 610041 China; 7 Division of Breast Surgery, Department of General Surgery, West China Hospital, Sichuan University, Chengdu, Sichuan 610041 China; 8 Translational Medicine Department, Amoy Diagnostics Co., Ltd., Xiamen, Fujian 361000 China; 9 LC-Bio Technology Co., Ltd., Hangzhou, Zhejiang 310000 China

**Keywords:** Breast cancer, Predictive markers, Tumour biomarkers

## Abstract

Chemotherapy serves as the primary therapeutic approach for triple-negative breast cancer (TNBC), yet its efficacy remains unsatisfactory. This study was a single-arm, open-label, single-center clinical trial (NCT05447702) involving patients with newly diagnosed stage II-III TNBC at West China Hospital. The treatment regimen consisted of camrelizumab (200 mg intravenously every 2 weeks, 12 cycles), apatinib (250 mg orally daily), and alternating chemotherapy [nab-paclitaxel (d1, 8, 15 every 4 weeks) for 4 cycles and epirubicin plus cyclophosphamide (every 2 weeks) for 4 cycles]. From June 2023 to April 2024, 35 patients were enrolled, of whom 1 patient withdrew due to adverse reaction intolerance. At treatment completion, the total pathological complete response (tpCR, ypT0/is, ypN0) rate was 67.6% (23/34), and the breast pCR (ypT0/is) rate was 70.6% (24/34). The overall response rate following neoadjuvant treatment reached 94.1% (32/34). Elevated levels of alanine aminotransferase (38.2%) and aspartate aminotransferase (29.4%) were the most common grade 3-4 adverse events, with no significant toxicities or treatment-related deaths reported. Comprehensive analysis of serum and tissue samples collected before and after neoadjuvant therapy via Olink and RNA sequencing revealed that the treatment induced a complex systemic immune response. These findings enabled the development of two novel scoring systems: a pretreatment response predictive score system for stratification and an efficacy assessment score system for treatment response evaluation. In conclusion, camrelizumab and apatinib combined with chemotherapy have good clinical efficacy and good safety as neoadjuvant treatments for stage II-III TNBC, warranting further investigation and potential clinical application.

## Introduction

Breast cancer (BC) represents the most commonly diagnosed malignancy and the primary cause of cancer-related death among women globally.^1,2^ Triple-negative breast cancer (TNBC), constituting roughly 15% of BC cases, is defined by the absence of estrogen receptor (ER), progesterone receptor (PR), and human epidermal growth factor receptor 2 (HER2) expression.^3,4^ Relative to other molecular subtypes, this aggressive subtype has distinct clinical features, including accelerated disease progression, early recurrence patterns, and diminished survival outcomes.^5^ The therapeutic landscape for TNBC remains particularly challenging due to its inherent biological heterogeneity and the scarcity of established molecular targets, resulting in a persistently poor prognosis over several decades.

Current standard-of-care predominantly relies on cytotoxic chemotherapy, although recent paradigm shifts have transitioned the emphasis of treatment from postoperative adjuvant regimens to preoperative neoadjuvant approaches. Emerging evidence from landmark clinical trials, including KEYNOTE-522, has further revolutionized management protocols, demonstrating superior outcomes with PD-1/PD-L1 immune checkpoint inhibitors (ICIs) combined with chemotherapy in early-stage, high-risk TNBC.^6^ However, critical analysis of phase III trial data (GeparNuevo, IMpassion031, KEYNOTE-522) reveals persistent limitations, with pathological complete response (pCR) rates plateauing at 53.4-64.8% and nearly half of patients do not achieve a pathologic complete response (non-pCR).^7–10^ The CamRelief trial established the clinical viability of camrelizumab, a humanized anti-PD-1 monoclonal antibody, combined with chemotherapy as neoadjuvant therapy (NAT) for early or locally advanced TNBC. The study enrolled patients across all nodal statuses, including those with N3 disease, and employed an intensive chemotherapy regimen consisting of nab-paclitaxel plus carboplatin, followed by dose-dense epirubicin plus cyclophosphamide. The results revealed a significantly greater pCR rate in the camrelizumab-chemotherapy group than in the placebo-chemotherapy group (56.8% *vs*. 44.7%). Notably, in stage III patients, the pCR rate was 49.4% with camrelizumab plus carboplatin-based chemotherapy versus with chemotherapy alone. These rates surpass those reported in the IMpassion031 trial (45% and 23%, respectively), further underscoring the potent antitumor activity of camrelizumab. Nevertheless, fatal adverse events occurred in 2 patients (0.9%) in the camrelizumab-chemotherapy group.^11^ Thus, these findings underscore the urgent need for innovative combinatorial strategies to increase immunotherapeutic efficacy.

Mounting preclinical and clinical evidence supports the synergistic potential of antiangiogenic agents in potentiating immune checkpoint blockade. Mechanistically, inhibiting the vascular endothelial growth factor (VEGF) pathway reshapes the tumor microenvironment by promoting immune cell infiltration, activating T-cells, and modulating PD-1/PD-L1.^12^ This scientific rationale has translated into clinical success, with combination regimens demonstrating improved survival outcomes in advanced TNBC settings.^13^ An open-label phase II trial (NCT03394287) revealed that the combination of apatinib, an orally bioavailable tyrosine kinase inhibitor that selectively targets vascular endothelial growth factor receptor-2 (VEGFR-2), with camrelizumab yielded favorable ORR and PFS in advanced TNBC patients, irrespective of prior lines of therapy or PD-­L1 status. The chemo-free regimen achieved an ORR of 43.3%, which was significantly greater than those reported for anti-PD-1/PD­-L1 antibody monotherapy (5.2%-18.5%) or apatinib alone (10.7%). These findings highlight the favorable clinical efficacy and acceptable safety profile of combination therapy.^14–18^

Despite these advancements, critical knowledge gaps persist. Notably, the trial of KEYNOTE-522 demonstrated the independence of PD-L1 expression in predicting the benefit of neoadjuvant immunotherapy, highlighting the importance of novel predictive biomarkers. Furthermore, substantial interpatient heterogeneity in treatment response necessitates precision medicine approaches to optimize therapeutic selection. To address these challenges, we designed this single-arm phase II trial investigating neoadjuvant camrelizumab combined with apatinib and chemotherapy in early or locally advanced TNBC. Following REMARK guidelines for tumor marker prognostic studies,^19^ our study incorporates prospective collection of tumor tissues and peripheral blood samples. Through comprehensive multiomics analyses, Our objectives were to (1) assess the regimen’s efficacy and safety profile; (2) identify predictive biomarkers for treatment response; and (3) search for potential efficacy-enhancing strategies for non-pCR TNBC patients, namely, the use of camrelizumab in conjunction with apatinib and chemotherapy.

## Results

### Patients

**Fig. 1:**
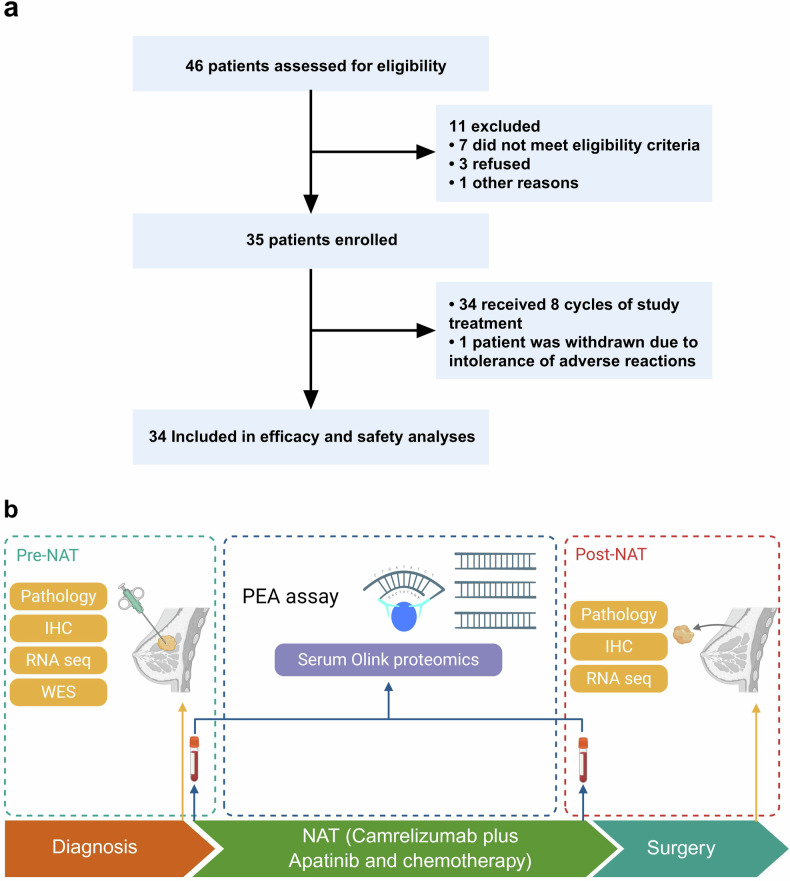
Flow chart of the study design and sample collection. **a** Flow charts of 34 patients were screened in this experiment. **b** The experimental design for the biomarker analysis. Created in BioRender. Luo, T. (2025) https://BioRender.com/05zm9gt

Between June 2023 and April 2024, a total of 46 TNBC patients who met the study criteria were screened. Among these patients, 35 were enrolled for NAT consisting of camrelizumab combined with apatinib and chemotherapy (Fig. 1a). One patient requested that treatment be stopped because of intolerable adverse reactions. After completing the second treatment cycle, the patient achieved a partial response (PR) but subsequently developed an erythematous rash and oral ulcers. The liver function results revealed that alanine aminotransferase (ALT) was 1091 U/L, and aspartate aminotransferase (AST) was 225 IU/L (grade 3 increase). These adverse effects were considered potentially related to the use of albumin-paclitaxel, camrelizumab, or apatinib. After 20 days of liver protection and antiallergic symptomatic treatment, transaminase levels decreased, and the rash subsided. Aside from this patient who withdrew due to adverse reactions, the remaining 34 patients (97.1%) completed the scheduled 8 cycles of NAT. All 34 remaining patients underwent efficacy and safety evaluation following NAT. The experimental design for the biomarker analysis is illustrated in Fig. 1b.

Table 1 Detailes the baseline clinical characteristics of the study cohort. The median age was 41 years (range, 32-67 years), with all patients exhibiting an Eastern Cooperative Oncology Group (ECOG) score of 0. HER2 status was scored as 0-1 in 29 patients (85.3%) and ≥2 in 5 patients (14.7%). Elevated Ki-67 levels (>30%) were observed in 26 patients (76.5%), and positive PD-L1 (CPS ≥ 1) was detected in 24 patients (70.6%).

**Table 1:** Baseline patient characteristics

	Total (*n* = 34)
Age (years), median (IQR)	41(32,67)
>40 year—*n* (%)	18 (52.9)
Menopausal status	
Premenopausal	17 (50)
Menopausal	17 (50)
ECOG status, n (%)	
0	34 (100)
1	0
HER2 status score, n (%)	
IHC 0-1	29 (85.3)
IHC 2+	5 (14.7)
Ki-67 level, *n* (%)	
≤30%	8 (23.5)
>30%	26 (76.5)
Clinical T stage, *n* (%)	
T1	1 (2.9)
T2	22 (64.7)
T3	10 (29.4)
T4	1 (2.9)
Clinical N stage, *n* (%)	
N0	7 (20.6)
N1	19 (55.9)
N2	2 (5.9)
N3	6 (17.6)
Overall clinical stage, *n* (%)	
Stage II	20 (58.8)
Stage III	14 (41.2)
PD-L1 status, *n* (%)^a^	
Positive	24 (70.6)
Negative	6 (17.6)
Unknown	4 (11.8)

*IQR* interquartile range, *ECOG* eastern cooperative oncology group, *HER2* human epidermal growth factor receptor 2

^a^PD-L1 positive is defined as a combined positive score of ≥1

### Efficacy and safety

**Fig. 2:**
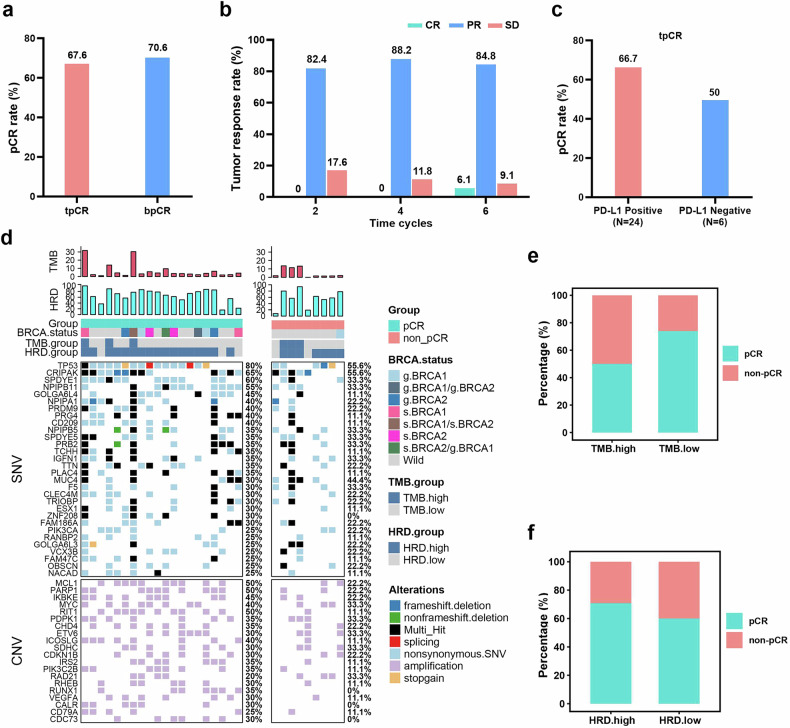
Efficacy analysis of camrelizumab plus apatinib and chemotherapy as neoadjuvant therapy (NAT). **a** Pathological complete response (pCR) rate of NATs. tpCR: total pCR; bpCR: breast pCR. **b** Imaging-assessed tumor response rates: complete response (CR); partial response (PR), stable disease (SD). **c** tpCR rate stratified by different PD-L1 expression levels. **d** Results of Whole-exon Sequencing (WES). The pCRs used in the subsequent experiments were all tpCRs. **e** Comparison of pCR *vs*. non-pCR patients distribution between the TMB-H and TMB-L groups. **f** Comparison of pCR *vs*. non-pCR patients distribution between the HRD-H and HRD-L groups

Among the 35 patients enrolled, only 1 patient withdrew from the trial due to intolerance of adverse reactions, and the remaining 34 patients participated in the evaluation of pCR (no residual cancer was found in the breast or lymph nodes after NAT). After NAT (Fig. 2a), 23 patients (67.6%, 95% CI, 49.5–82.6) achieved tpCR (ypT0/is, ypN0), and the rate of breast pCR (bpCR, ypT0/is) was 70.6% (95% CI, 52.5–84.9). Subgroup analysis revealed higher pCR rates in patients with stage III disease than in those with stage II disease (71.4% *vs*. 65%), nodal-negative versus nodal-positive (100.0% *vs*. 59.3%), T3/T4 versus T2 disease (T3: 70%; T4: 100%; combined T3/T4: 72.7% *vs*. T2: 68.1%), and N2/N3 versus N1 nodal involvement (N2: 100%; N3: 80%; combined N2/N3: 85.7% *vs*. N1: 50%). Notably, patients with more advanced disease (stage III) and a greater number of nodal-positive lymph nodes tended to have a relatively high pCR rates.

Bilateral breast MRI was performed in all patients before and during NAT. The number of patients with stable disease (SD) progressively declined throughout the treatment course (Fig. 2b). After 6 cycles of treatment, 2 patients achieved a complete response (CR). Additionally, the tpCR rate of the PD-L1-positive subgroup was greater than that of the PD-L1-negative subgroup (66.7% *vs*. 50.0%, Fig. 2c). The overall response rate (ORR) was 94.1% (32/34) after NAT. At least one treatment-related adverse event (AE) occurred in all 34 patients who were evaluated (Table 2). Elevated levels of alanine aminotransferase (13 patients, 38.2%) and aspartate aminotransferase (10 patients, 29.4%) were the most frequent grade 3-4 AEs. The possible immune-related AEs, such as hypothyroidism (12 patients, 35.3%) and rash (11 patients, 32.4%), were all grade 1 or 2. A single case of grade 3 immune-related pneumonitis was reported. No significant toxicities or treatment-related deaths occurred during the study.

**Table 2:** Adverse events

	All (*n* = 34)		
**Treatment-emergent adverse events (TEAEs),** ***n*** **(%)**	**Any grade**	**Grade 1-2**	**Grade** ≥ **3**
Any TEAE	34 (100)	30 (88.2)	4 (11.8)
Any TEAE leading to dose interruption/delay	21 (61.8)	18 (52.9)	3 (8.8)
Nausea	8 (23.5)	8 (23.5)	0
Alopecia	34 (100)	34 (100)	0
Anemia	19 (55.9)	19 (55.9)	0
Decreased platelet count	4 (11.8)	4 (11.8)	0
Decreased neutrophil count	6 (17.6)	4 (11.8)	2 (5.9)
Fatigue	15 (44.1)	15 (44.1)	0
Diarrhea	10 (29.4)	10 (29.4)	0
Increased aspartate aminotransferase	26 (76.5)	16 (47.1)	10 (29.4)
Increased alanine aminotransferase level	26 (76.5)	13 (38.2)	13 (38.2)
Vomiting	1 (2.9)	1 (2.9)	0
Asthenia	15 (44.1)	15 (44.1)	0
Constipation	10 (29.4)	10 (29.4)	0
Decreased white blood cell count	9 (26.5)	7 (20.6)	2 (5.9)
Rash	11 (32.4)	11 (32.4)	0
Peripheral neuropathy	5 (14.7)	5 (14.7)	0
Hypertension	3 (8.8)	3 (8.8)	0
Hand-foot syndrome	18 (52.9)	18 (52.9)	0
**Immune-related adverse events (irAEs),** ***n*** **(%)**	**Any grade**	**Grade 1-2**	**Grade** ≥ **3**
Any irAE	11 (32.4)	10 (29.4)	1 (2.9)
Reactive cutaneous capillary endothelial proliferation	5 (14.7)	5 (14.7)	0
Rash	11 (32.4)	11 (32.4)	0
Infusion reactions	1 (2.9)	1 (2.9)	0
Hypothyroidism	12 (35.3)	12 (35.3)	0
Hyperthyroidism	2 (5.9)	2 (5.9)	0
Adrenal insufficiency	3 (8.8)	3 (8.8)	0
Pneumonitis	1 (2.9)	0	1 (2.9)
Thyroiditis	6 (17.6)	6 (17.6)	0
Hypophysitis	1 (2.9)	1 (2.9)	0

### Genomic heterogeneity in TNBC baseline tissues

Whole-exon sequencing (WES) was used to analyze exon mutations in samples from 20 pCR (pCRs in the subsequent experimental data were all tpCRs) patients and 9 non-pCR patients. The baseline tumor mutation profiles are illustrated in Fig. 2d. The mutated single nucleotide change (SNV/indel/frameshift) or amplification that occurred in each case is shown in one vertical column. The patient’s BRCA mutation status and the total number of tumor mutations in each patient are displayed separately at the top of the figure. In addition, germline mutations were excluded, and only somatic mutations are presented. Genomic analysis showed that the two most common mutations were *TP53* (pCR 80%, non-pCR 55.6%) and *CRIPAK* (pCR 60%, non-pCR 55.6%) in the pCR and non-pCR groups, respectively. Copy number variation (CNV) profiles differed substantially between groups: *MCL1* and *PARP1* were predominant in the pCR group, whereas the non-pCR group presented higher frequencies of *MYC*, *PDPK1*, *ETV6*, *SDHC*, and *RAD21* (each with *n* = 3). The proportion of HRD-H patients was 85% (*n* = 17/20) in the pCR group and 77.8% (*n* = 7/9) in the non-pCR group. There was no significant difference in TMB (*P* = 0.2) or HRD (*P* = 0.36) between the groups (Supplementary Fig. 1a and 1b). The distribution of pCR and non-pCR patients was not significantly different between the TMB-H and TMB-L groups (*P* = 0.339, Fig. 2e). Similar results were observed when the patients were stratified into HRD-H and HRD-L groups (*P* = 0.633, Fig. 2f). Four patients carried *BRCA* germline pathogenic mutations (*BRCA1*, *n* = 3; *BRCA2*, *n* = 1), with 3 patients in the pCR group and 1 patient in the non-pCR group. Notably, one patient presented both a somatic *BRCA2* mutation and a pathogenic germline *BRCA1* mutation (Supplementary Fig. 1c).

### Dynamics of plasma immune proteomics are associated with the response to NAT

The Olink proteomics method was employed to analyze the serum protein expression levels in TNBC patients before and after NAT. A total of 33 pretreatment and 30 posttreatment serum samples were collected. The Olink Target 96 Immuno-Oncology Panel was utilized to detect the expression levels of 92 marker proteins in key immune and tumor pathways. Comparison of serum protein levels before and after treatment revealed dynamic changes in serum immunoproteomics following NAT. Significant changes were observed in 31 of 92 tested proteins, which are involved in various immune-related signaling pathways (Supplementary Fig. 2), indicating that the NAT elicited a complex systemic immune response. Among them, the serum levels of MUC-16 significantly decreased after NAT.

### Serum IL-18 levels and biopsy PD-L1 levels predict the response to neoadjuvant chemotherapy

**Fig. 3:**
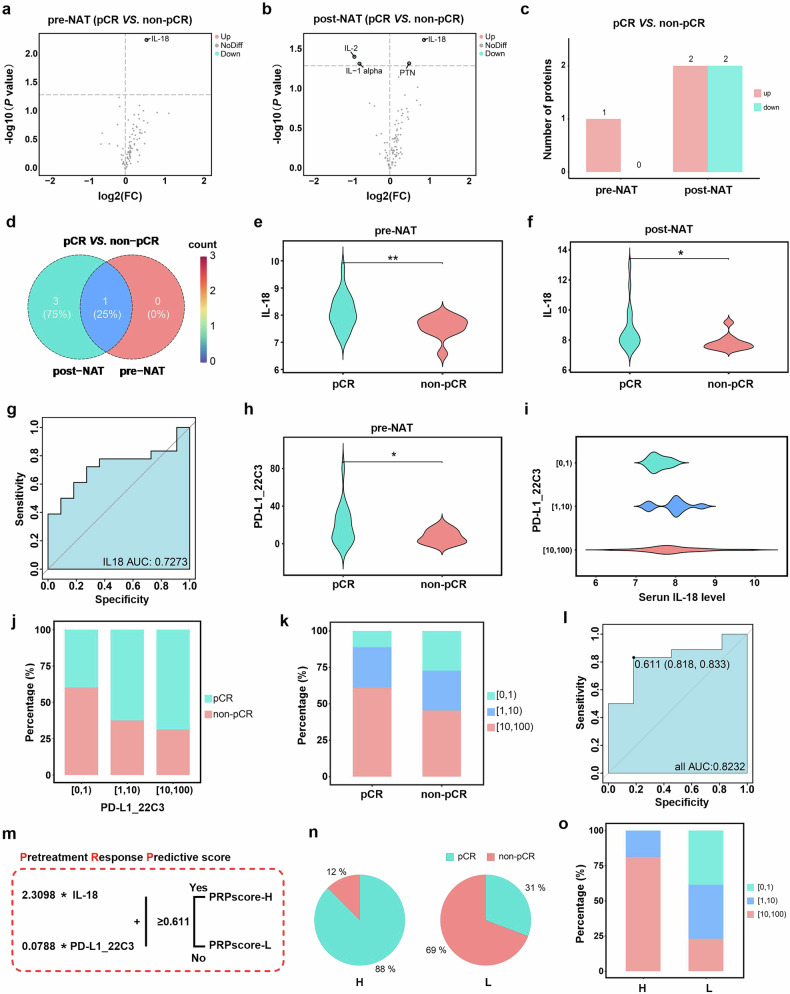
Pre-NAT serum IL-18 and biopsy PD-L1 levels predict the response to NAT. **a** Comparison of pre-NAT serum protein levels between pCR patients and non-pCR patients. **b** Comparison of post-NAT serum protein levels between the pCR and non-pCR patients. **c** The amount of protein that significantly changed in the serum between pCR patients and non-pCR patients. **d** Venn diagram showing altered protein intersections before and after NAT. **e**, **f** Compared with non-pCR patients, pCR patients had higher IL-18 expression before and after treatment. **g** ROC curve assessing the accuracy of the pre-NAT serum IL-18 level for treatment response. **h** pCR patients had higher PD-L1 levels in biopsy samples before treatment than non-pCR patients did. **i** No significant difference in pretreatment serum IL-18 levels between different biopsy PD-L1 staining scores. **j** Treatment response in patients with different biopsy PD-L1 staining scores. **k** Biopsy PD-L1 staining scores of patients with different treatment responses. **l** ROC curve evaluating the pretreatment response predictive (PRP) score’s accuracy for treatment response prediction. **m** Formula for the PRP score. **n** Treatment response in patients stratified by high *vs*. low PRP scores. **o** Percentages of different biopsy PD-L1 staining scores in PRPscore-high and PRPscore-low patients. [0,1): 0 ≤ PD-L1 score<1, [1,10):1 ≤ PD-L1 score<10, [10,100): 10 ≤ PD-L1 score<100; *P < 0.05, **P < 0.01

To identify an effective method for predicting pCR rates, we compared the serum immune proteomic profiles between pCR patients and non-pCR patients. Only 1 protein, IL-18, was significantly different before treatment, with higher expression levels observed in the pCR patients than in the non-pCR patients (Fig. 3a, c). We also compared the absolute changes in protein levels after chemotherapy between the two groups. Compared with those in non-pCR patients, IL-18 and PTN levels in pCR patients increased, whereas IL-2 and IL1-alpha levels in pCR patients decreased (Fig. 3b, c). Venn diagram analysis revealed that IL-18 was the only protein consistently elevated in pCR patients before and after NAT (Fig. 3d, e, f). Notably, the levels of serum IL-18 did not differ significantly before and after treatment in the overall patient cohort, pCR patients or non-pCR patients (Supplementary Fig. 3). These findings suggest that pre-NAT serum IL-18 levels may serve as a potential marker for predicting the efficacy of NAT. To further refine our predictive capabilities, we developed a pretreatment response predictive score (PRP score) system. Although pretreatment serum IL-18 levels varied among TNBC patients, the AUC value for IL-18 alone was only 0.7273 (Fig. 3g), indicating limited predictive power as a standalone marker.

In our study, the tpCR rate was 66.7% in PD-L1-positive patients (Fig. 2c). Further investigation revealed that PD-L1 expression was significantly greater in biopsy samples from pCR patients than in those from non-pCR patients (Fig. 3h, *P* < 0.05). Furthermore, no significant differences in serum IL-8 levels were observed among patients with varying biopsy PD-L1 scores (Fig. 3i). Additionally, patients with higher PD-L1 scores presented higher pCR rates, and the proportion of patients with high PD-L1 scores was also greater in the pCR group (Fig. 3j, k).

We explored the combined use of pre-NAT serum IL-18 levels and biopsy PD-L1 levels to predict the response to NAT, which consisted of camrelizumab plus apatinib and chemotherapy. The ROC logistic regression curve of the combined model revealed that the AUC value reached 0.8232 (95% CI, 0.818–0.833), and the cutoff value was 0.611 (Fig. 3l). Patients were divided into PRPscore-high and PRPscore-low groups (Fig. 3m). Those with high PRP scores exhibited a significantly better treatment response (pCR=88%), whereas patients with low PRP scores had a worse treatment response (Fig. 3n). Furthermore, a greater proportion of patients with high PRP scores also had elevated PD-L1 levels (Fig. 3o). The PRP score system, which incorporates pre-NAT serum IL-18 and biopsy PD-L1 data, may provide a valuable tool for stratifying TNBC patients and identifying those unlikely to benefit from this treatment regimen. However, the sensitivity and specificity of the PRP score require further validation in larger clinical studies.

### Analysis of immune infiltration and immune-related genes in pCR and non-pCR patients at baseline

**Fig. 4:**
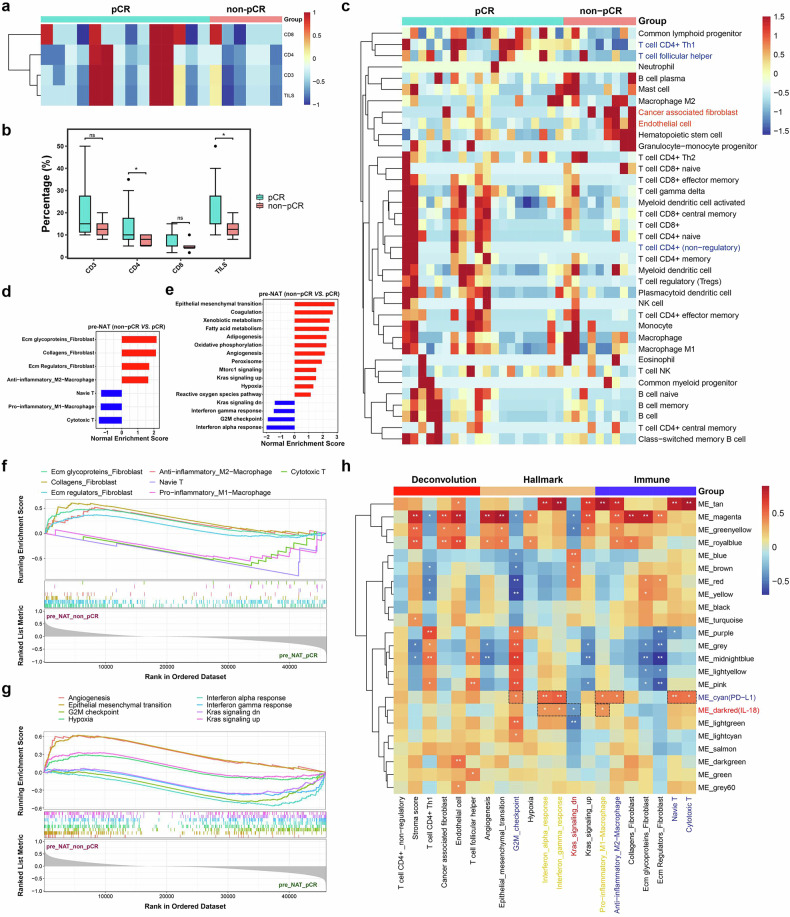
Analysis of immune infiltration and immune-related genes in pCR and non-pCR patients at baseline. **a**, **b** CD3, CD4, CD8 and TIL levels were detected by immunohistochemistry in pCR and non-pCR patients. **c** Deconvolution of the RNA-seq data was performed to analyze the proportions of immune cell subtypes. **d** Screened single-cell immune-related gene sets that conformed to the expected direction of change. **e** Screened for significant hallmark gene sets of change. **f** GSEA line plots showing the significantly altered single-cell immune-related gene sets. **g** GSEA line plots showing the screened hallmark gene sets that fit the expected changes. **h** Weighted gene coexpression network analysis (WGCNA) evaluating correlations between gene modules and phenotypic characteristics. *P < 0.05

The levels of CD3, CD4, CD8 and TILs in the patient samples were detected via immunohistochemistry. Compared with those in the pCR group, there were significantly fewer CD4^+^ TILs and TILs in the samples from non-pCR patients (Fig. 4a, b). The proportions of various immune cell subtypes were analyzed via RNA-seq deconvolution. Compared with those in the pCR group, the numbers of CD4^+^ T cells (nonregulatory), CD4^+^ Th1 T cells and follicular helper T cells in non-pCR patients were significantly lower, whereas the numbers of cancer-associated fibroblasts and endothelial cells were significantly greater (Fig. 4c, Supplementary Fig. 4).

To explore the relationship between the tumor microenvironment and the immune response, gene sets related to immune cell function were analyzed via GSEA/GSVA. The results revealed that in the non-pCR group, the activities of the collagens, ecm glycoproteins and ecm regulators, which are closely related to fibroblasts, were significantly increased. Moreover, the naive score and cytotoxicity score, which reflect T-cell infiltration, decreased, whereas the M1-related proinflammatory macrophage score decreased, and the anti-inflammatory score of M2 macrophages increased (Fig. 4d, f). To analyze the characteristics of non-pCR tumors more accurately, the hallmark gene set was introduced for GSEA/GSVA scoring. The results revealed that the tumor cells in non-pCR patients presented more significant malignant characteristics. The KRAS, epithelial mesenchymal transition, G2/M checkpoint, hypoxia, and angiogenesis pathways were significantly upregulated, whereas the interferon alpha response and interferon gamma response were significantly decreased in non-pCR patients (Fig. 4e, g).

Weighted gene coexpression network analysis (WGCNA) was performed on the basis of the bulk RNA-seq data. Genes were divided into 23 modules, and correlation analysis was conducted between the modules and cell proportions obtained from deconvolution, scores of cell characteristic gene sets, and scores of hallmark gene sets. Among them, we focused on the darkred module containing IL-18 and the cyan module containing PD-L1 (Fig. 4h). Both were strongly correlated with immune features, indicating that they may further affect the formation of the tumor immune microenvironment by regulating the functions of immune cells.

### Efficacy assessment score to identify potential strategies for increasing the pCR rate

**Fig. 5:**
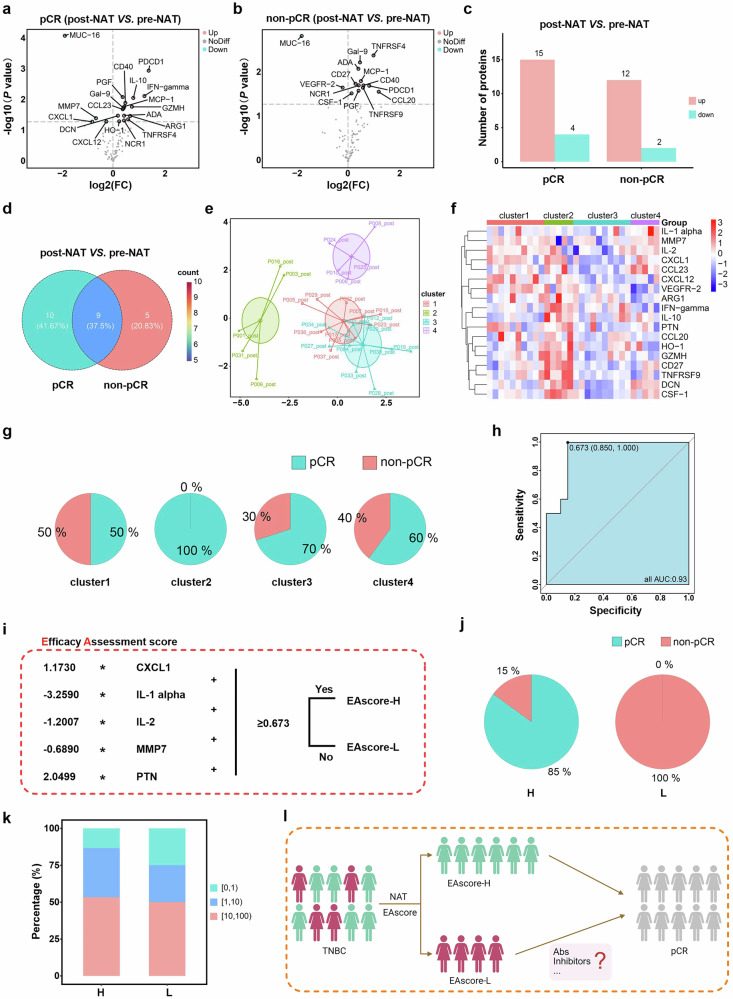
An efficacy assessment score was used to determine the possibility of increasing the pCR rate. **a** Volcano plot of serum proteins changes after NAT at pCR. **b** Volcano plot of serum proteins changes after NAT in non-pCR patients. **c** The amount of protein that significantly changed in pCR and non-pCR serum. **d** Venn diagram showing altered protein intersections in pCR and non-pCR serum samples. **e** Clustering analysis of post-NAT serum protein levels identifying 4 patient subgroups. **f** Post-NAT serum protein levels of the four clusters. **g** Treatment response in four clusters of patients. **h** ROC curve evaluating the predictive accuracy of the post-NAT serum efficacy assessment score (EA score) for treatment response. **i** Formula for the EA score. **j** Treatment response in high *vs*. low EA scores patients. **k** Percentages of different biopsy PD-L1 staining scores in EAscore-high and EAscore-low patients. [0,1): 0 ≤ PD-L1 score<1, [1,10):1 ≤ PD-L1 score<10, [10,100): 10 ≤ PD-L1 score<100. **l** Post-NAT patient stratification strategy based on the EA score. Created in BioRender. Luo, T. (2025) https://BioRender.com/6potstj

To explore potential strategies for enhancing efficacy in non-pCR patients and improving the pCR rate among patients receiving this treatment, we compared the dynamics of serum immune proteomics before and after NAT in patients with different treatment responses. Compared with the pretreatment samples, 19 proteins were significantly altered in the pCR patients, whereas 14 proteins were changed in the non-pCR patients (Fig. 5a−c). A Venn diagram revealed 5 unique protein changes in non-pCR patients and 10 in pCR patients following NAT (Fig. 5d).

A total of 18 proteins were included in the consensus clustering, comprising 3 differentially expressed proteins between non-pCR patients and pCR patients after NAT (Supplementary Fig. 5), 10 unique proteins in post-NAT pCR patients (Supplementary Fig. 6), and 5 unique proteins in post-NAT non-pCR patients (Supplementary Fig. 7). K-means cluster analysis was applied to the expression data of the screened proteins in post-NAT serum samples, categorizing patients into distinct subtypes. Cluster 2 was associated with a significantly better treatment response (Fig. 5e−g). This clustering result may be linked to the clinical characteristics of the patients and the uniquely expressed proteins.

To identify a potential approach for increasing the pCR rate, we developed an efficacy assessment score (EA score) system. We selected 3 proteins that were differentially expressed between non-pCR patients and pCR patients after NAT (IL-1 alpha, IL-2 and PTN), along with 2 proteins that uniquely changed in pCR patients after NAT (CXCL1 and MMP7), which presented the largest AUC values. These 5 post-NAT serum proteins (IL-1 alpha, IL-2, PTN, CXCL1, and MMP7) were used to construct the EA scoring formula. The ROC curve of the EA score yielded an AUC of 0.93 (95% CI, 0.850–1.000), with a cutoff value of 0.673 (Fig. 5h). Patients were stratified into EAscore-high and EAscore-low groups (Fig. 5i), and the EAscore-low group was associated with a significantly poorer treatment response (100%, Fig. 5j). Additionally, no significant difference was observed in the proportion of patients with varying PD-L1 expression levels across the different EA score groups (Fig. 5k). The EA score system is useful for stratifying TNBC patients receiving NAT and identifying those who may not benefit from this therapy. For EAscore-low patients, we wondered whether the pCR rate could be improved by timely administration of other appropriate agents, such as antibodies or inhibitors (Fig. 5l).

To further investigate this possibility, we analyzed protein and gene expression changes before and after NAT via Olink and RNA sequencing, followed by enrichment of potentially involved signaling pathways. RNA sequencing revealed 2109 genes that were differentially expressed genes between pCR and non-pCR samples before NAT, and 7282 genes were altered in non-pCR patients after NAT (Supplementary Fig. 8a-d). Through Venn diagram analysis, 69 genes that were abnormally upregulated or downregulated in non-pCR patients were identified (Supplementary Fig. 8e-f). KEGG pathway enrichment analysis highlighted hallmark signaling pathways associated with the 18 proteins and 69 genes (Supplementary Fig. 9). Notably, the IL-17 signaling pathway was identified in both the gene and protein KEGG enrichment results, suggesting that the poor response to camrelizumab and apatinib combined with chemotherapy in EAscore-low patients may be attributed to dysregulation of the IL-17 signaling pathway. This pathway may represent a potential target for improving the pCR rate of this NAT regimen.

## Discussion

In this single-center, prospective, nonrandomized clinical trial, we evaluated the efficacy, safety, and potential predictive biomarkers for neoadjuvant camrelizumab and apatinib in combination with chemotherapy in patients with previously untreated early or locally advanced TNBC. To the best of our knowledge, this is the first trial to report the efficacy and safety of combining camrelizumab and apatinib in this patient population. This regimen resulted in a significant improvement in total pathological complete response (pCR) and a manageable toxicity profile, irrespective of PD-L1 status. Considering that pCR after NAT is associated with a favorable prognosis in TNBC patients,^20,21^ these findings are especially promising.

This trial focused primarily on enhancing the efficacy of ICIs plus chemotherapy through antiangiogenic therapy, while also optimizing chemotherapy protocols. Antiangiogenic therapy has been shown to reverse resistance to ICIs and enhance treatment effects, offering significant survival benefits in advanced TNBC patients.^13,18^ This enhancement is mediated by promoting CD8^+^ T cell infiltration and activation within the tumor microenvironment, alongside antiangiogenic-induced PD-L1 upregulation.^22,23^ In our study, the combination of apatinib with camrelizumab and chemotherapy further supported the notion that antiangiogenic therapy can augment the efficacy of immunotherapy, demonstrating promising results in early or locally advanced TNBC. Patients with previously untreated stage II-III TNBC were enrolled to receive NAT consisting of 12 cycles of camrelizumab (200 mg every 2 weeks), apatinib (250 mg daily), and nab-paclitaxel (d1, 8, 15 every 4 weeks), followed by an additional 4 cycles of camrelizumab and epirubicin-cyclophosphamide (every 2 weeks). The chemotherapy regimen was designed on the basis of efficacy, side effects, immune response activation, and optimal treatment duration. Anthracycline- and taxane-based neoadjuvant chemotherapy remains the standard treatment for early or locally advanced TNBC. Short-term induction via doxorubicin and cisplatin has been shown to create a favorable tumor microenvironment and enhance the clinical response to PD-1 inhibitors in TNBC.^24^ However, steroid premedication for cytotoxic chemotherapy may weaken the antitumor immune response triggered by ICIs.^25^ Therefore, nab-paclitaxel, which does not require steroid premedication, may serve as an ideal chemotherapeutic agent for combination strategies with ICIs.^26,27^ In this study, we selected a regimen of nab-paclitaxel and epirubicin-cyclophosphamide without platinum to strengthen the antitumor potential of camrelizumab.

The incorporation of camrelizumab and apatinib into neoadjuvant chemotherapy led to a significant increase in the proportion of patients achieving pCR at the time of definitive surgery. The pCR rate observed in this study surpassed those reported in the KEYNOTE-522 trial (64.8%) with neoadjuvant pembrolizumab plus platinum-containing chemotherapy,^9^ the IMpassion031 trial (58%) with neoadjuvant atezolizumab plus sequential nab-paclitaxel and anthracycline-based chemotherapy,^7^ and the CamRelief trial (56.8%) with neoadjuvant camrelizumab plus nab-paclitaxel and anthracycline-based chemotherapy.^11^ Further studies should explore whether differing chemotherapy regimens influence antitumor efficacy when combined with immunotherapy and antiangiogenic therapy. Notably, the proportion of patients with N3 disease varied across these studies (KEYNOTE-522: 0/784, 0%; IMpassion031: 10/165, 6%; CamRelief: 23/222, 10.4%). In this study, 5 (14.7%) patients with N3 disease were included, 4 of whom achieved pCR (4/5, 80%). The pCR benefit of camrelizumab combined with apatinib was observed across several prespecified subgroups, including those defined according to PD-L1 expression, tumor size and nodal status. These results suggest that the addition of camrelizumab and apatinib improves the total pCR rate in a subgroup of patients with a historically poor prognosis.

The observed AEs aligned with the established safety profiles of camrelizumab monotherapy, apatinib, nab-paclitaxel, and anthracycline-containing chemotherapy. Adding camrelizumab and apatinib did not substantially exacerbate common chemotherapy-related toxic effects. These findings are consistent with the known safety profiles of platinum-free neoadjuvant chemotherapy in early or locally advanced TNBC, as well as safety camrelizumab’s profiles in early or metastatic TNBC.^13,18,28^ In this trial, the hypertension and hand‒foot syndrome observed were associated with apatinib, whereas rash, hypothyroidism, pneumonitis, and capillary hemangioma were linked to camrelizumab. Reactive capillary endothelial proliferation, a common immune-related side effect linked to camrelizumab, occurred in only 14.7% of patients receiving camrelizumab plus apatinib. Other AEs were likely attributed to the combination therapy. The most frequent severe (grade≥3) treatment-related AEs were elevated alanine aminotransferase (ALT) (13 [38.2%]) and aspartate aminotransferase levels (AST) (10 [29.4%]). A prior meta-analysis suggests platinum-free neoadjuvant chemotherapy carries a lower risk of grade 3 or 4 hematological AEs (e.g., neutropenia, anemia, and thrombocytopenia) than platinum-based regimens do, according to a prior meta-analysis.^29^ Overall, these AEs were relatively well tolerated, with low rates of discontinuation and dose reduction. No treatment-related deaths occurred during the study. Further research is warranted, as long-term survival outcomes remain under evaluation.

TMB and HRD have been previously reported as potential biomarkers associated with immunotherapy efficacy in cancer treatment.^30,31^ In our study, although comparative genomic profiling analysis revealed distinct genomic heterogeneity between treatment response groups, TMB and HRD status exhibited no statistically significant intergroup differences. These findings suggest that additional, more robust biomarkers may exist to better predict NAT efficacy. Notably, serum IL-18 levels did not change in either pCR or non-pCR patients after NAT, yet pre-NAT and post-NAT expression was greater in pCR patients. IL-18 treatment enhances antitumor immunity, and components of the IL-18 pathway have been reported to be upregulated in tumor-infiltrating lymphocytes.^32^ IL-18, originally termed interferon-gamma-inducing factor, is a cytokine produced by various cells and tissues that bridges innate and adaptive immunity.^33^ IL-18 may play a dual role in tumor immune biology. On the one hand, IL-18 gene expression and serum levels are significantly higher in BC patients than in healthy individuals.^34^ IL-18 expression is low or negative in benign breast fibroadenomas but is elevated in tumor cells from most BC patients, with positive expression associated with lymph node metastasis.^35^ These findings suggest that high IL-18 expression in BC cells may promote the progression of BC. On the other hand, IL-18 is believed to exert antitumor effects by inducing IFN-gamma expression. Previous studies have shown that IL-18 can suppress the growth and spread of BC, suppress angiogenesis, and induce tumor cell apoptosis.^36,37^ IL-18-secreting chimeric antigen receptor T cells have been shown to increase CAR-T-cell proliferation and antitumor activity.^38^ Additionally, IL-18 synergizes with IL-12 to increase NK cell activity and induce tumor cell apoptosis.^36^ In this study, pCR patients presented increased serum IL-18 levels before and after treatment, and IL-18 expression was unaffected by NAT. Furthermore, we also observed increased IFN-gamma expression in pCR patients after NAT (supplementary Fig. 6) but not in the non-pCR patients. We speculate that IL-18 may induce the expression of other proteins, such as IFN-gamma, which could explain the favorable response to NAT in these patients. Whether increasing IL-18 levels in TNBC patients can enhance their response to camrelizumab plus apatinib combined with chemotherapy is also a subject of further study.

We also evaluated whether pre-NAT serum IL-18 could predict treatment response. However, ROC analysis revealed that the AUC value of pre-NAT serum IL-18 alone was insufficient to support its use as a standalone predictive marker. We further incorporated biopsy PD-L1 expression into the predictive model, and the ROC logistic regression curve of pre-NAT serum IL-18 combined with biopsy PD-L1 expression yielded an AUC value of 0.8232. According to the formula, patients with high PRP scores exhibited a significantly better treatment response (pCR=88%). The PRP scoring system may aid in stratifying TNBC patients, although its sensitivity and specificity require further clinical validation.

When the relationship between the tumor microenvironment and the immune response was studied, the activities of the collagens, ECM glycoproteins and ECM regulators, which are closely associated with fibroblasts, were significantly increased in non-pCR. Moreover, the naive score and cytotoxicity score reflecting T-cell infiltration decreased, whereas the proinflammatory score of M1 macrophages decreased, and the anti-inflammatory score of M2 macrophages increased. On the basis of these results, we speculate that fibroblasts in non-pCR patients may construct the extracellular matrix by secreting collagen and other substances, thereby forming a barrier that encloses cancer nests, hindering the further infiltration of T cells and weakening the ability of T cells to target and kill tumor cells effectively. In addition, there may be specific interactions between the active factors released by fibroblasts and macrophages in non-pCR. This interaction may be key in promoting macrophage polarization from the proinflammatory M1 phenotype to the anti-inflammatory M2 phenotype, further intensifying the formation and development of the tumor immunosuppressive tumor microenvironment.

The tumor cells in non-pCR patients presented more significant malignant characteristics. KRAS mutation is an important factor that promotes the progression of lung cancer. The significant upregulation of KRAS signaling revealed that tumor cells in non-pCR have a greater degree of tumor malignancy. The activities of the epithelial mesenchymal transition and G2/M checkpoint pathways were significantly increased in non-pCR patients, suggesting that tumor cells in non-pCR patients have greater advantages in terms of invasion, metastasis and proliferation ability. Furthermore, the increase in the activities of the hypoxia and angiogenesis pathways attracted our attention. Although antiangiogenic therapy was adopted during the treatment process, the massive consumption of oxygen and nutrients by tumor cells in non-pCR with strong proliferation ability during proliferation induced the formation of the tumor hypoxic microenvironment, and the a hypoxic tumor microenvironment, in turn, promoted a further increase in the activity of the angiogenesis pathway. This led to the failure of the antiangiogenic therapy to achieve the expected therapeutic effect. We also observed that the activities of the key pathways involved in the inflammatory response, interferon alpha response and interferon gamma response decreased significantly, providing strong evidence for the determination of the immunosuppressive microenvironment in non-pCR patients. IL-18 and PD-L1 were incorporated into our established PRP scoring system. The coexpression modules strongly correlated with immune cell infiltration levels and functional scores.

To identify strategies for increasing the pCR rate, we developed an efficacy assessment score (EA score) system. Patients with low EA scores were associated with a significantly poorer treatment response (100%). This scoring system could help identify patients unlikely to benefit from the current treatment, enabling timely intervention with alternative therapies. There is currently a lack of dedicated ongoing clinical trials or robust preclinical data specifically investigating IL-17 inhibition in TNBC. Pathway analysis revealed that the IL-17 signaling pathway, involving the genes FOS and FOSB and the protein CCL20, was upregulated in the non-pCR group after treatment (supplementary Fig. 9). Reportedly, the FOS and FOSB genes activate and upregulate the IL-17 signaling pathway,^39^ which promotes the expression of the cancer-promoting gene CCL20.^40^ The activation of the IL-17 signaling pathway in non-pCR patients may explain their poor response to therapy. Inhibiting this pathway with targeted agents or antibodies could improve pCR rates in patients with low EA scores. Notably, the anti-IL-17A monoclonal antibody vunakizumab has been approved for treating moderate-to-severe plaque psoriasis.^41^ Whether vunakizumab combined with NAT can improve pCR rates in EAscore-low patients warrants further investigation.

Although this is the first trial to evaluate neoadjuvant anti-PD-1 therapy combined with anti-VEGFR2 therapy and chemotherapy in previously untreated early or locally advanced TNBC patients, several limitations should be acknowledged. First, the sample size was relatively small, and larger randomized controlled trials are required to validate the efficacy of this regimen for TNBC. Second, as an exploratory study, it did not evaluate the efficacy of this apatinib‒camrelizumab regimen in comparison with checkpoint inhibition,antiangiogenic monotherapies or other chemotherapy combinations. Further phase III trials are needed to directly evaluate apatinib plus camrelizumab against camrelizumab monotherapy or apatinib monotherapy combined with chemotherapy in the neoadjuvant TNBC setting. Third, long-term outcomes and safety data are critical for assessing potentially curative treatments. The contributions of adjuvant ICIs to treatment efficacy and the optimal management of residual disease remain to be determined. Follow-up and further analyses to evaluate overall survival and long-term safety are currently in progress. Additionally, the predictive scoring system and conclusions drawn from this study require further validation in subsequent clinical trials.

In short, our study comprehensively evaluated the efficacy and the safety profile of combining camrelizumab and apatinib with chemotherapy for the treatment of stage II-III TNBC. Through systematic analysis of serum and tissue samples from TNBC patients, we identified a complex systemic immune response following NAT. On the basis of these findings, we established two novel scoring systems: a pretreatment response predictive score system for stratification and an efficacy assessment score system for treatment response evaluation. The combination regimen demonstrated significant clinical efficacy and favorable safety outcomes, indicating that it deserves further investigation and clinical implementation as a therapeutic strategy in TNBC treatment.

## Methods

### Study design and patients

This single-arm, open-label, single-center phase II trial (NCT05447702, MA-BC-II-026) investigated the safety and antitumor efficacy of camrelizumab combined with apatinib and chemotherapy in previously untreated TNBC patients. The study protocol received approval from the Ethics Review Committee of West China Hospital (No. 1523, 2021) and was conducted in compliance with good clinical practice principles. Written consent was obtained from all patients prior to their enrollment, with recruitment occurring between June 2023 and April 2024.

This study employs a single-arm design. NCSS PASS 15 statistical software (LLC. Kaysville, Utah, USA, ncss.com/software/pass), it was calculated that enrolling approximately 31 subjects would yield a 95% confidence interval of [44%, 78%] for the tpCR rate in the experimental group. Considering a 10% dropout rate, this study requires a total enrollment of 35 subjects.

The key eligibility criteria were as follows: 1) female patients between the ages of 18 and 75 years with newly diagnosed BC; 2) histologically confirmed early or locally advanced TNBC as defined by the latest ASCO/CAP guidelines; 3) Eastern Cooperative Oncology Group (ECOG) score of 0–1; and 4) expected survival time of not less than 3 months; 5) present at least one measurable lesion as defined by the Response Evaluation Criteria in Solid Tumors (RECIST) 1.1; and 6) adequate organ function. Patients were excluded if they had 1) inflammatory, metastatic or bilateral BC; 2) received any antitumor treatment within the last 12 months; 3) had severe cardiac diseases; 4) previously received PD-1/PD-L1 antibodies, CTLA-4 antibodies, or other PD-1/PD-L1 inhibitor therapy; or 5) had a definite history of previous neurological or psychiatric disorders, including epilepsy or dementia, and if they had a known history of psychotropic substance abuse, alcohol abuse, or drug use.

### Treatments and endpoints

In this single-center, prospective, nonrandomized clinical trial, patients with newly diagnosed stage II-III TNBC received 12 cycles of camrelizumab (at a dose of 200 mg) every 2 weeks, apatinib (at a dose of 250 mg) every day, and nab-paclitaxel (125 mg/m^2^ d1, 8, 15) every 4 weeks, and then received an additional 4 cycles of epirubicin (90 mg/m^2^) - cyclophosphamide (600 mg/m^2^) every 2 weeks. The primary endpoint was defined as the total pathological complete response (tpCR) rate, whereas the secondary endpoints included the breast pathological complete response (bpCR), overall response rate (ORR), and incidence of treatment-related adverse events (AEs).

### PD-L1 immunohistochemical staining

PD-L1 immunohistochemical analysis was performed on 4-μm-thick tissue sections from formalin-fixed, paraffin-embedded (FFPE) samples. Specific staining was performed via the use of monoclonal antibodies against PD-L1 (DAKO 22C3) following standardized IHC protocols. PD-L1 positivity was determined via the combined positive score (CPS) metric, with a threshold of a CPS ≥ 1 considered positive. The CPS calculation included all PD-L1-expressing cells, including tumor cells, lymphocytes, and macrophages, divided by the total number of tumor cells multiplied by 100.

### Plasma Olink Proteomics analysis

Proteomic analysis was conducted at the LC-Bio Technology Co., Ltd. (Hangzhou, Zhejiang, China) via the Olink Proteomics Target 96 Immuno-Oncology Panel. This high-throughput platform employs proximity extension assay (PEA) technology, a dual recognition immunoassay methodology that combines antibody-based protein detection with nucleic acid amplification. Following target protein recognition by matched antibody pairs, DNA-based reporter molecules undergo proximity-dependent extension, enabling precise quantification of 92 immuno-oncology-related biomarkers through real-time quantitative polymerase chain reaction (qPCR). Protein expression levels were quantified via normalized protein expression (NPX) values, a relative log2-scale measurement derived from standardized data transformation. NPX values are positively correlated with absolute protein concentrations, with higher values indicating greater analyte abundance. The complete list of 92 proteins analyzed in this panel is provided in Supplementary Table 1.

### Whole-exon sequencing (WES) and RNA sequencing (RNAseq)

DNA and RNA were isolated from FFPE specimens via the AmoyDx®MagPure FFPE DNA LQ Kit and the AmoyDx® FFPE RNA Extraction Kit following the manufacturers’ protocols. For WES, DNA libraries were constructed employing the xGen® Exome Research Panel v1 (IDT:1056115) for targeted capture. Library quality control was performed with KAPA HotStart ReadyMix for amplification, Qubit fluorometry for quantification, and an Agilent 2100 Bioanalyzer for size distribution analysis.

Following library pooling, 2 × 150 bp end sequencing was performed on the NovaSeq 6000 platform. The sequencing data were analyzed and annotated by ANDAS. The raw sequencing reads underwent quality control processing, including adapter trimming and removal of low-quality sequences (quality<15) and poly N stretches, followed by alignment to the human reference genome version 19 (hg19). PCR repeats were tagged and removed. The final VCF files were obtained by comparing Indels and nucleotide polymorphisms (SNPs). Somatic short variants (SNVs) and indels were filtered on the basis of (i) ≥5 supporting reads and ≥5% variant allele frequencies supporting the variant; (ii) present >2% population frequency in the 1000 g or ExAC or GnomAD databases; (iii) non-CDS localization; and (iv) the variant was not annotated as (likely/predicted) carcinogenic in the OncoKB database. The resulting high-confidence variants were retained for functional characterization.

The TMB of a tumor sample is determined according to the literature and is calculated by the number of nonsynonymous somatic mutations (single nucleotide variants and small insertions/deletions) per megabase (MB) in coding regions, as previously reported.^42^ TMB-high status was defined as ≥10 mut/MB. HRD status was assessed via the Genomic Scar Score (GSS) model (25) from AmoyDx by considering the length, type and location of genome-wide chromosomal CNVs through 24,000 single nucleotide polymorphisms (SNPs) distributed across the human genome.^43^ The GSS was calculated via sum loss of heterozygosity (LOH), large-scale state transition (LST), and telomeric allelic imbalance (TAl) via ANDAS software (Amoy Diagnostics, Xiamen, China). HRD-positivity was defined as a GSS score ≥50.

RNA-seq was also performed on a Novaseq 6000 platform. We constructed a transcriptome based on the GRCh37/hg19 reference genome via STAR 2.7 and estimated the transcript abundance in transcripts per million (TPM) with RSD v1.3.3. Read alignment and quantification were performed via a GRCh37/hg19-based transcriptome reference, with sequence mapping conducted via STAR (v2.7) and transcript abundance estimation calculated in TPM units via RSEM (v1.3.3).

### Statistical analysis

Differentially expressed proteins in the Olink data were identified via the OlinkAnalyze package (version 3.4.1; Olink_ttest function). Visualization of the proteomic profiles was performed via the OmicStudio platform (https://www.omicstudio.cn/tool) to generate heatmaps and volcano plots. Functional annotation analyses were performed through enrichment analysis of Gene Ontology (GO) terms and Kyoto Encyclopedia of Genes and Genomes (KEGG) pathways. The results were supplemented by Reactome pathway, InterPro domain, and Disease Ontology (DO) analyses. All enrichment evaluations were conducted through hypergeometric testing, with functional terms achieving *P* < 0.05 considered statistically significant for differential protein expression. The WoLF PSORT bioinformatics tool (https://wolfpsort.hgc.jp/) was employed to predict protein subcellular localization patterns. Protein‒protein interaction (PPI) networks were constructed by extracting interactions with confidence scores ≥0.4 from the STRING database, followed by network visualization. The diagnostic performance of treatment response biomarkers was evaluated by analyzing receiver operating characteristic (ROC) curves via the pROC function in the R package, with AUC values quantifying classification accuracy. A significance level of *P* < 0.05 was applied for all the statistical tests. Analyses were conducted via the R statistical computing environment (version 4.1.0; R Foundation for Statistical Computing).

## Supplementary information

Supplementary Materials

Statistical Analysis Plan

study protocol

Raw data for olink detection

## Data Availability

The raw data have been deposited in the Genome Sequence Archive for Human (GSA-Human) at the National Genomics Data Center (NGDC) under BioProject accession number PRJCA042227. Datasets generated in this study are available from the corresponding author upon request via email at luoting@wchscu.cn, use for commercial purposes is prohibited. All requests will be reviewed by the corresponding author within 2 weeks. Prior to data sharing, a signed data access agreement with the sponsor is required.
