# Supplementary material for: Exploratory phase II trial of an anti-PD-1 antibody camrelizumab combined with a VEGFR-2 inhibitor apatinib and chemotherapy as a neoadjuvant therapy for triple-negative breast cancer (NeoPanDa03): efficacy, safety and biomarker analysis
Source: Signal Transduct Target Ther. 2025 Jul 21;10:237. doi: 10.1038/s41392-025-02337-1 (PMC12279984; doi:10.1038/s41392-025-02337-1)
Supplement: Supplementary file 1 — Supplementary Materials [file 41392_2025_2337_MOESM1_ESM.docx]

Supplementary Materials for

Exploratory phase Ⅱ trial of an anti-PD-1 antibody camrelizumab combined with a VEGFR-2 inhibitor apatinib and chemotherapy as a neoadjuvant therapy for triple-negative breast cancer (NeoPanDa03): efficacy, safety and biomarker analysis

Xiaoxiao Liu, Chunying Zhuang, Lei Liu, Ling Xiong, Xin Xie, Ping He, Juanjuan Li, Bing Wei, Xi Yan, Tinglun Tian, Xiaorong Zhong, Jie Chen, Yan Cheng, Dan Zheng, Peng Cheng, Tianlin Sun, Weiwei Li, Changbin Zhu, Shuaitong Chen, Chao Fang, Jun Fu, Shibao Li, Jing Jing, Ting Luo

Correspondence to: drliuxx@126.com; sdjnshlb@xzhmu.edu.cn; jingjing@wchscu.edu.cn; luoting@wchscu.cn

**This PDF file includes:**

Figures. S1 to S9

Tables S1

**Other Supplementary Materials for this manuscript include the following:**

Supplementary Data S1

**
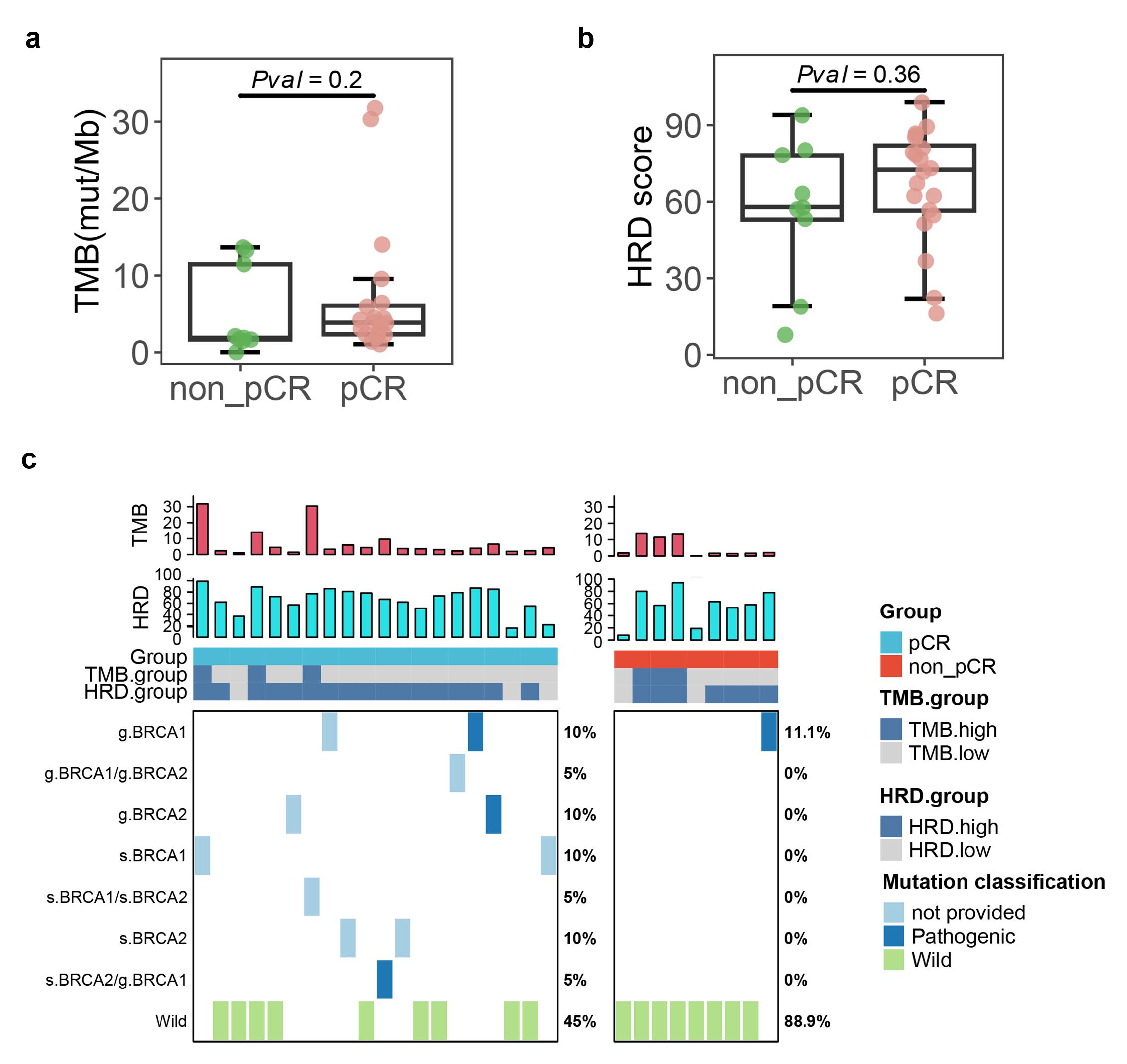
**

Figure. S1. WES results are further analyzed.

**a** TMB expression levels in pCR and non-pCR patients. **b** HRD expression levels in pCR and non-pCR patients. **c** Analysis of BRCA germline pathogenic mutations.



Figure. S2. The dynamics of serum immune proteomics associated with response to neoadjuvant therapy (NAT).

**a** Quantification of protein significantly altered in serum following NAT. **b** Volcano plot illustrating serum proteins with altered expression levels post-NAT. **c** GO enrichment analysis of proteins of proteins affected by NAT. **d** KEGG enrichment analysis of proteins altered post-NAT. **e** All proteins significantly changed after NAT. **f** Correlation analysis between the abundance of 92-panel proteins in pre-NAT serum and selected clinical indicators.


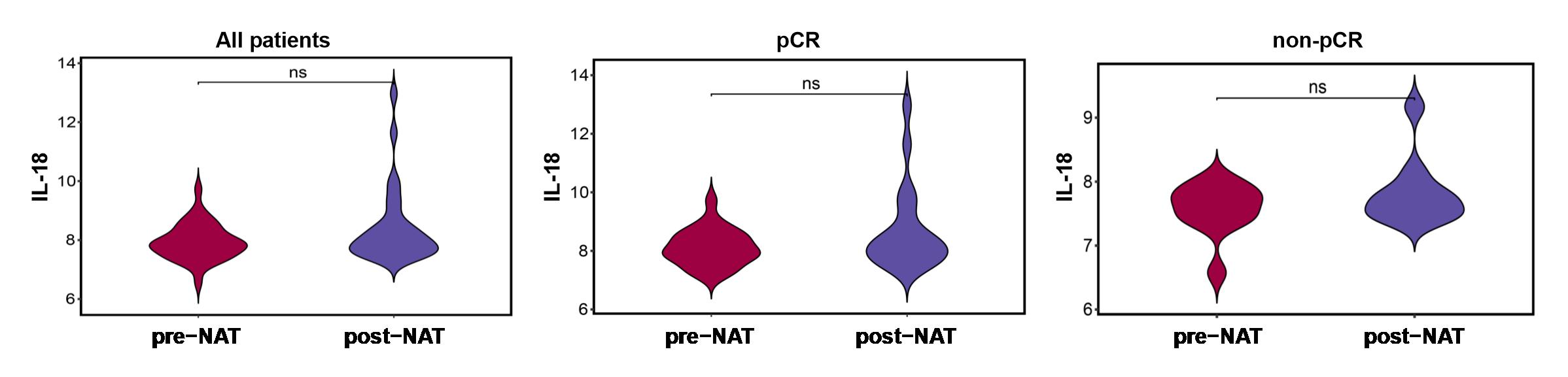


Figure. S3. Comparison of serum IL-18 levels before and after NAT in all patients, pCR or non-pCR.


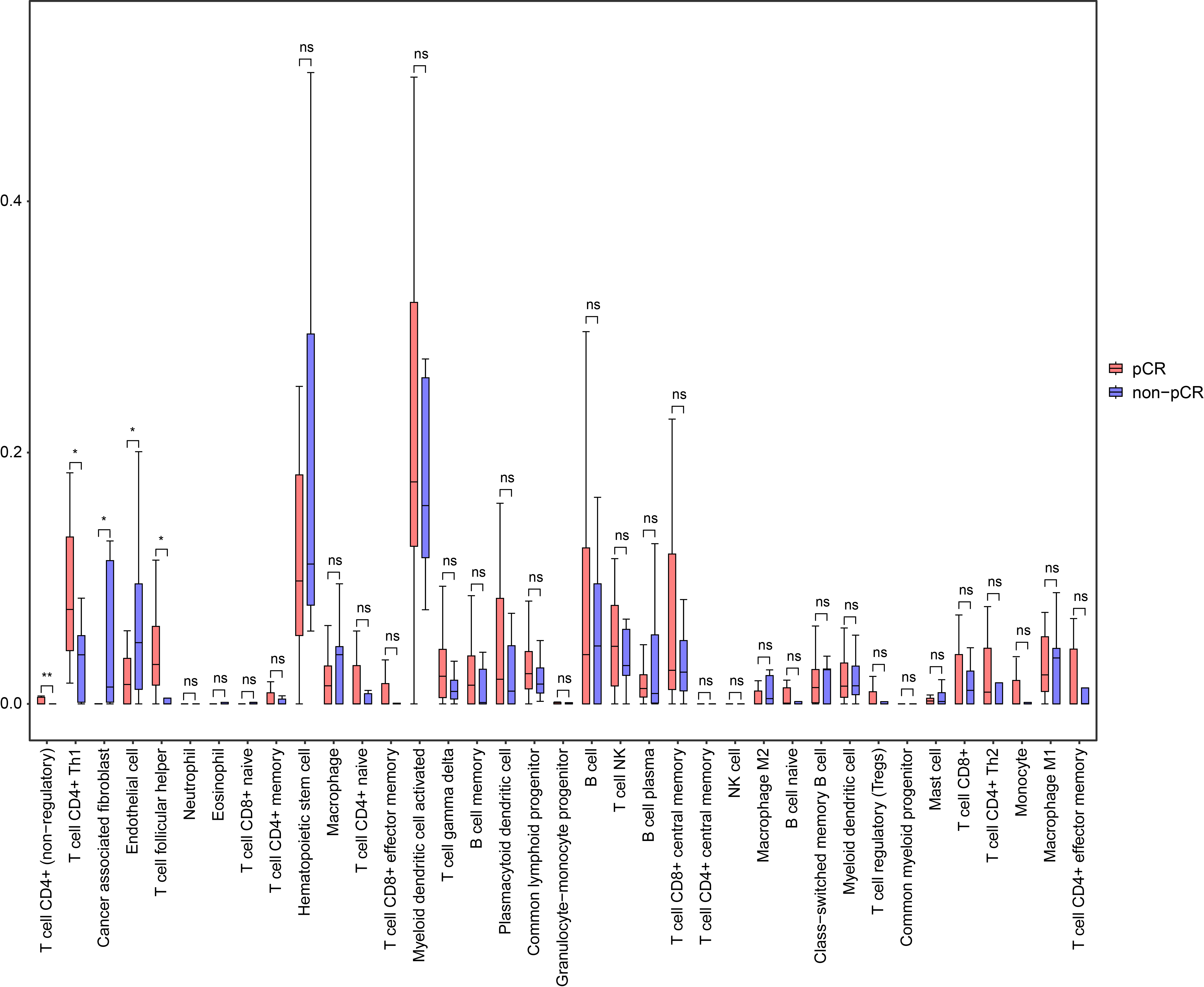


Figure. S4. The proportion of various immune cell subtypes in pCR and non-pCR was analyzed by RNA-seq deconvolution.


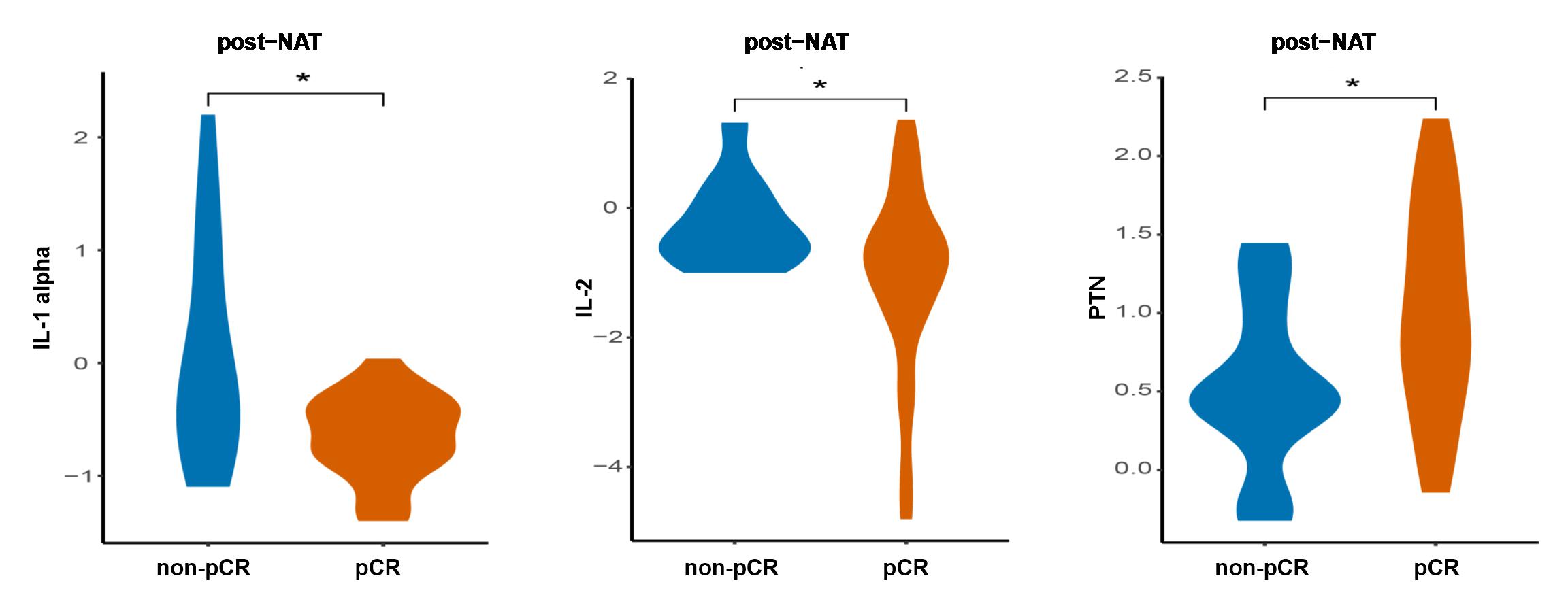


Figure. S5. Except for IL-18, the other three differentially expressed proteins between pCR and non-pCR after NAT. * *P*<0.05.


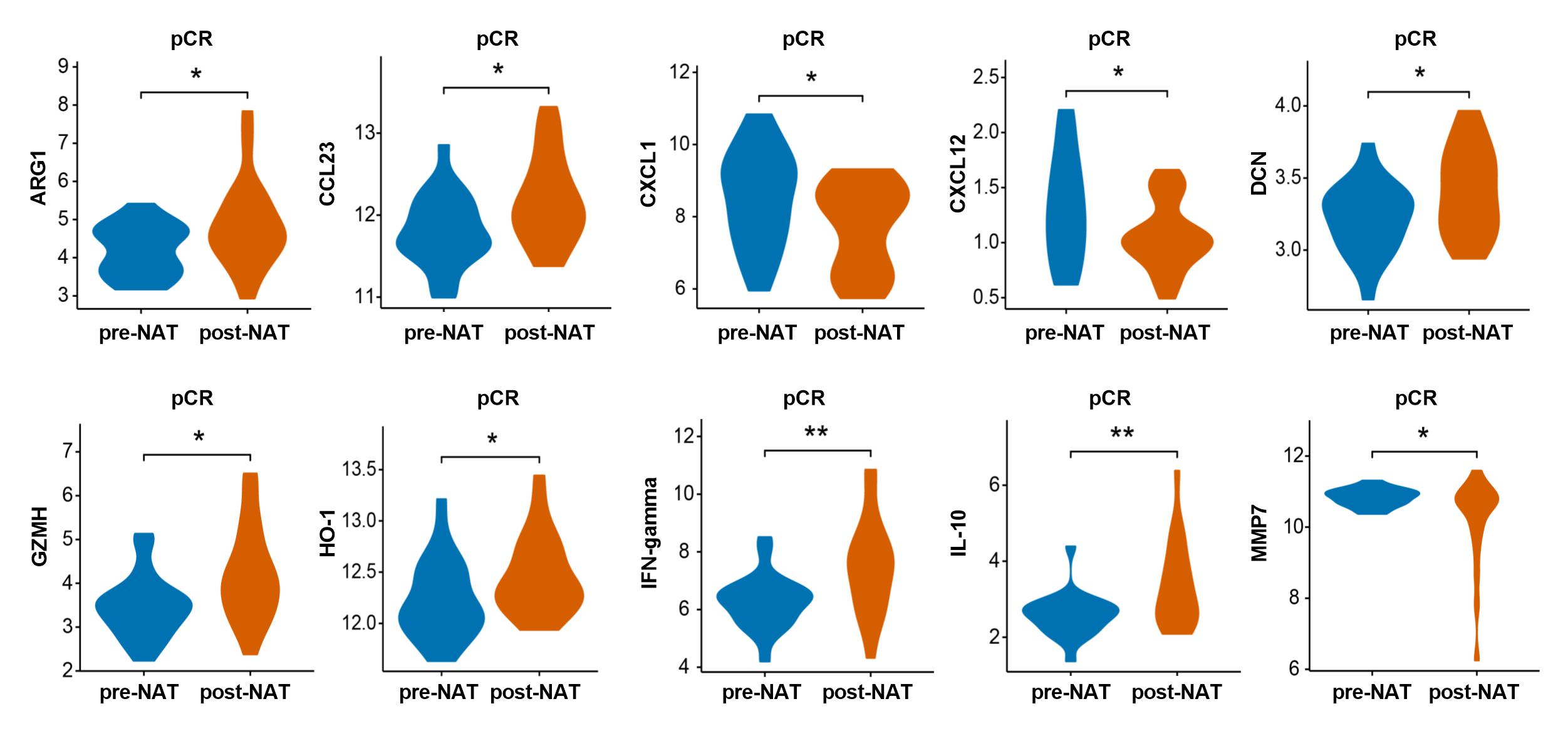


Figure. S6. Ten proteins that changed only in pCR following NAT. * *P*<0.05,** *P* <0.01.


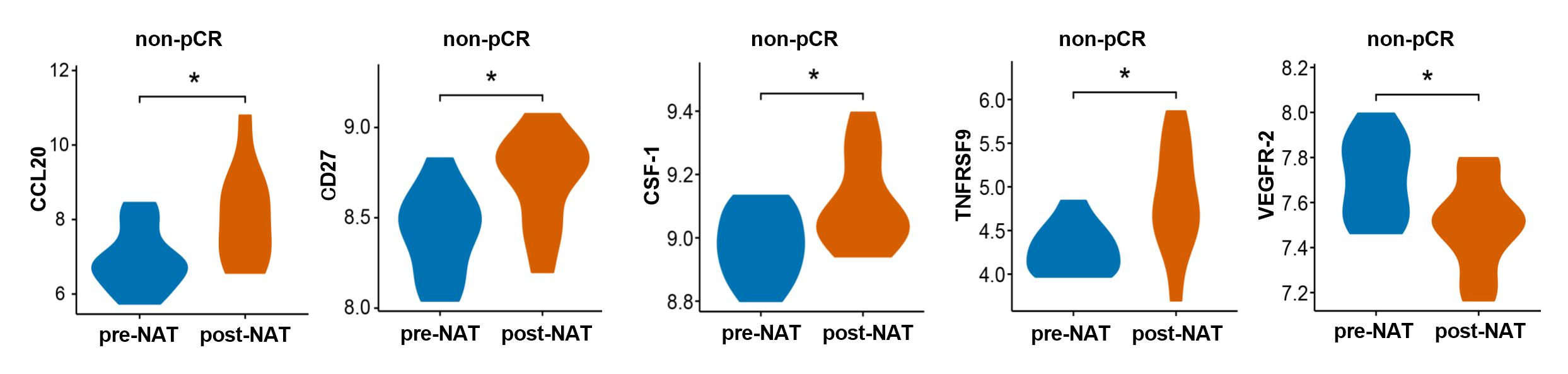


Figure. S7. Five proteins that changed only in non-pCR after NAT. * *P*<0.05.


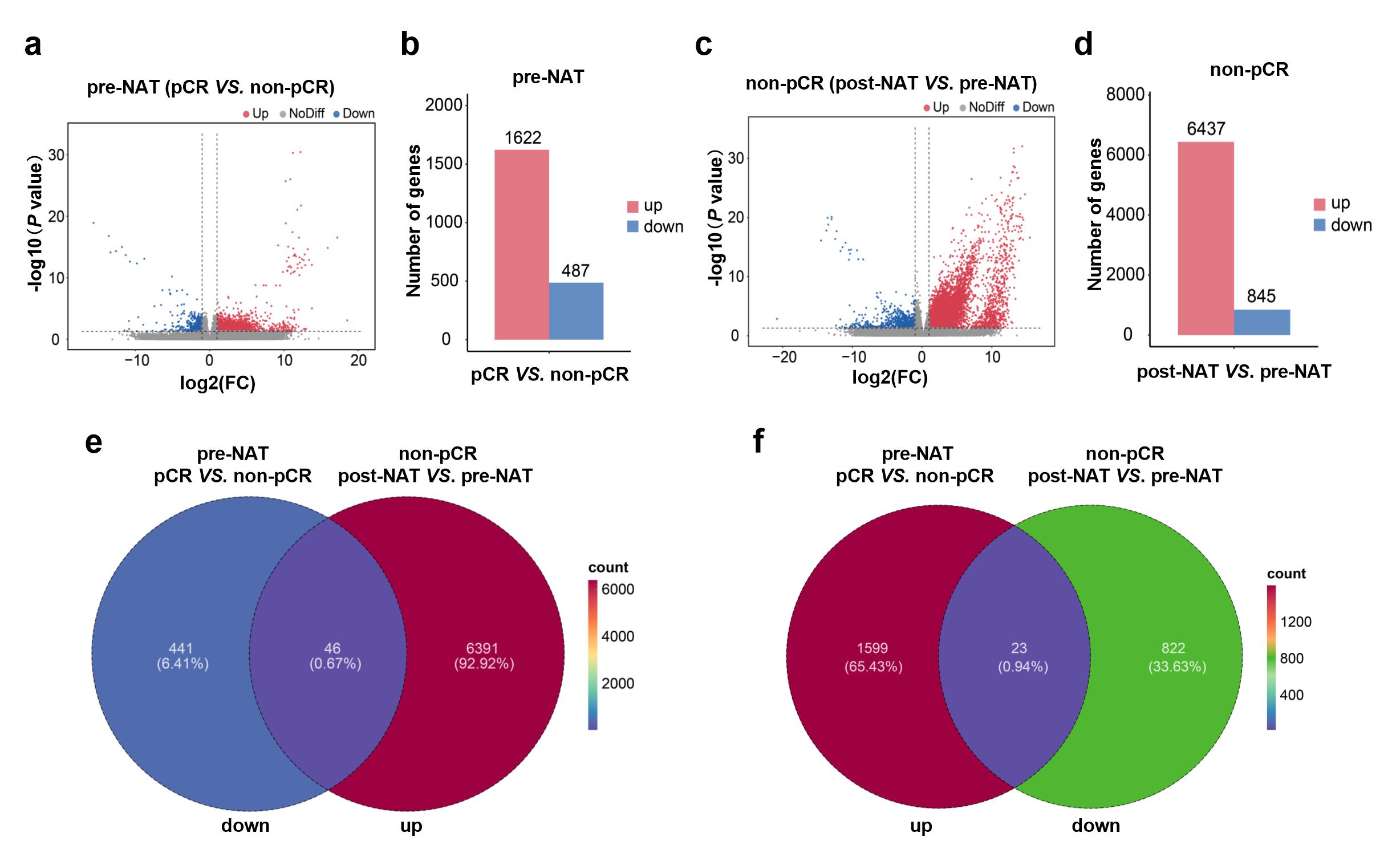


Figure. S8. Dynamics of genes associated with response to NAT.

**a** Volcano plot illustrating differentially expressed genes between pCR and non-pCR group prior to NAT. **b** The amount of differential genes between pCR and non-pCR before NAT. **c** Volcano plot demonstrating gene expression changes following NAT adminstration. **d** The amount of genes that significantly changes post-NAT. **e** Venn diagram depicting the number of genes showing further increased expression in non-pCR patients after NAT. **f** Venn diagram illustrating the number of genes further decreased in the non-pCR following NAT.





Figure. S9. Investigation of signaling pathways potentially associated with enhanced pCR rates in EAscore-low patients.

**a** Screened 18 proteins. **b** Screened 69 genes, the screening method is shown in Supplementary Fig. S8e and f. **c** KEGG pathway enrichment analysis for 18 proteins. **d** KEGG pathway enrichment analysis for 69 genes.

Table S1. All 92 marker proteins included in Olink Target 96 Immuno-Oncology panel

| **UniProt** | **Protein target** | **Abbreviation** |
| --- | --- | --- |
| P00813 | Adenosine deaminase | ADA |
| Q9Y653 | Adhesion G-protein coupled receptor G1 | ADGRG1 |
| Q15389 | Angiopoietin-1 | ANGPT1 |
| O15123 | Angiopoietin-2 | ANGPT2 |
| P05089 | Arginase-1 | ARG1 |
| Q16790 | Carbonic anhydrase 9 | CAIX |
| Q14790 | Caspase-8 | CASP-8 |
| Q92583 | C-C motif chemokine 17 | CCL17 |
| Q99731 | C-C motif chemokine 19 | CCL19 |
| P78556 | C-C motif chemokine 20 | CCL20 |
| P55773 | C-C motif chemokine 23 | CCL23 |
| P10147 | C-C motif chemokine 3 | CCL3 |
| P13236 | C-C motif chemokine 4 | CCL4 |
| Q9BZW8 | Natural killer cell receptor 2B4 | CD244 |
| P26842 | CD27 antigen | CD27 |
| P10747 | T-cell-specific surface glycoprotein CD28 | CD28 |
| P01730 | T-cell surface glycoprotein CD4 | CD4 |
| P25942 | Tumor necrosis factor receptor superfamily member 5 | CD40 |
| P29965 | CD40 ligand | CD40-L |
| P06127 | T-cell surface glycoprotein CD5 | CD5 |
| P32970 | CD70 antigen | CD70 |
| Q01151 | CD83 antigen | CD83 |
| P01732 | T-cell surface glycoprotein CD8 alpha chain | CD8A |
| O95727 | Cytotoxic and regulatory T-cell molecule | CRTAM |
| P09603 | Macrophage colony-stimulating factor 1 | CSF-1 |
| P78423 | Fractalkine | CX3CL1 |
| P09341 | Growth-regulated alpha protein | CXCL1 |
| P02778 | C-X-C motif chemokine 10 | CXCL10 |
| O14625 | C-X-C motif chemokine 11 | CXCL11 |
| P48061 | Stromal cell-derived factor 1 | CXCL12 |
| O43927 | C-X-C motif chemokine 13 | CXCL13 |
| P42830 | C-X-C motif chemokine 5 | CXCL5 |
| Q07325 | C-X-C motif chemokine 9 | CXCL9 |
| hP07585 | Decorin | DCN |
| P01133 | Pro-epidermal growth factor | EGF |
| P48023 | Tumor necrosis factor ligand superfamily member 6 | FASLG |
| P09038 | Fibroblast growth factor 2 | FGF2 |
| P09382 | Galectin-1 | Gal-1 |
| O00182 | Galectin-9 | Gal-9 |
| P12544 | Granzyme A | GZMA |
| P10144 | Granzyme B | GZMB |
| P20718 | Granzyme H | GZMH |
| P14210 | Hepatocyte growth factor | HGF |
| P09601 | Heme oxygenase 1 | HO-1 |
| O75144 | ICOS ligand | ICOSLG |
| P01579 | Interferon gamma | IFN-gamma |
| P01583 | Interleukin-1 alpha | IL-1 alpha |
| P22301 | Interleukin-10 | IL10 |
| P29459_P29460 | Interleukin-12 | IL12 |
| P42701 | Interleukin-12 receptor subunit beta-1 | IL12RB1 |
| P35225 | Interleukin-13 | IL13 |
| P40933 | Interleukin-15 | IL15 |
| Q14116 | Interleukin-18 | IL18 |
| P60568 | Interleukin-2 | IL2 |
| O95760 | Interleukin-33 | IL33 |
| P05112 | Interleukin-4 | IL4 |
| P05113 | Interleukin-5 | IL5 |
| P05231 | Interleukin-6 | IL6 |
| P13232 | Interleukin-7 | IL7 |
| P10145 | Interleukin-8 | IL8 |
| P43629 | Killer cell immunoglobulin-like receptor 3DL1 | KIR3DL1 |
| Q13241 | Natural killer cells antigen CD94 | KLRD1 |
| P18627 | Lymphocyte activation gene 3 protein | LAG3 |
| Q9UQV4 | Lysosome-associated membrane glycoprotein 3 | LAMP3 |
| P01137 | Transforming growth factor beta-1 | LAP TGF-beta-1 |
| P13500 | C-C motif chemokine 2 | MCP-1 |
| P80075 | C-C motif chemokine 8 | MCP-2 |
| P80098 | C-C motif chemokine 7 | MCP-3 |
| Q99616 | C-C motif chemokine 13 | MCP-4 |
| Q29983_Q29980 | MHC class I polypeptide-related sequence A/B | MIC-A/B |
| P39900 | Macrophage metalloelastase | MMP12 |
| P09237 | Matrilysin | MMP7 |
| Q8WXI7 | Mucin-16 | MUC-16 |
| O76036 | Natural cytotoxicity triggering receptor 1 | NCR1 |
| P29474 | Nitric oxide synthase, endothelial | NOS3 |
| Q15116 | Programmed cell death protein 1 | PDCD1 |
| P01127 | Platelet-derived growth factor subunit B | PDGF subunit B |
| Q9NZQ7 | Programmed cell death 1 ligand 1 | PD-L1 |
| Q9BQ51 | Programmed cell death 1 ligand 2 | PD-L2 |
| P49763 | Placenta growth factor | PGF |
| P21246 | Pleiotrophin | PTN |
| Q02763 | Angiopoietin-1 receptor | TIE2 |
| P01375 | Tumor necrosis factor | TNF |
| Q9NP84 | Tumor necrosis factor receptor superfamily member 12A | TNFRSF12A |
| O75509 | Tumor necrosis factor receptor superfamily member 21 | TNFRSF21 |
| P43489 | Tumor necrosis factor receptor superfamily member 4 | TNFRSF4 |
| Q07011 | Tumor necrosis factor receptor superfamily member 9 | TNFRSF9 |
| O43557 | Tumor necrosis factor ligand superfamily member 14 | TNFSF14 |
| P50591 | Tumor necrosis factor ligand superfamily member 10 | TRAIL |
| O43508 | Tumor necrosis factor ligand superfamily member 12 | TWEAK |
| P15692 | Vascular endothelial growth factor A | VEGFA |
| P35968 | Vascular endothelial growth factor receptor 2 | VEGFR-2 |

Supplementary Data S1. (separate file)

Raw data for olink detection
