# Supplementary material for: Exploratory phase II trial of an anti-PD-1 antibody camrelizumab combined with a VEGFR-2 inhibitor apatinib and chemotherapy as a neoadjuvant therapy for triple-negative breast cancer (NeoPanDa03): efficacy, safety and biomarker analysis
Source: Signal Transduct Target Ther. 2025 Jul 21;10:237. doi: 10.1038/s41392-025-02337-1 (PMC12279984; doi:10.1038/s41392-025-02337-1)
Supplement: Supplementary file 2 — Statistical Analysis Plan [file 41392_2025_2337_MOESM2_ESM.pdf]

Statistical Analysis Plan (SAP)

HYDRATION TO OPTIMIZE METABOLISM

Principal Investigator	Ting Luo
Protocol identification number	V2.0（dated 2024-6-28）
ClinicalTrials.gov identifier	NCT05447702
Author	Ting Luo
Version	Draft 1.0

SIGNATURE PAGE

Principal Investigator

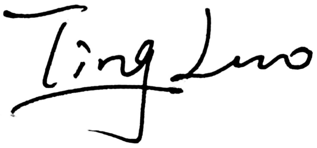

Ting Luo (2023-09-08)

Author

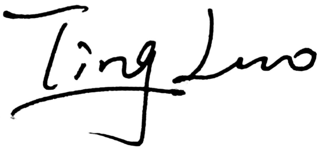

Ting Luo(2023-09-08)

Abbreviations

PFS	Progression-Free-Survival
PPS	Per-Protocol Set
PR	Partial response
RECIST	Response evaluation criteria in solid tumors
SAE	Serious adverse event
SAP	Statistical analysis plan
SAS	-
SD	Stable disease
SOP	Standard Operation Procedure
SS	Safety Analysis set
TNBC	Triple-negative breast cancer
AE	Adverse event
ORR	Objective response rate
OS	Overall survival
P	Phosphorus
PD	Progressive disease
PD-1/PD-L1	Programmed death 1/programmed death ligand 1

Table of contents

SIGNATURE PAGE 2

1. Introduction 5

2. Study design 5

2.1 Sample size calculation 5

3. Aims and objectives 5

4. Outcomes 5

4.1 Primary outcome 5

4.2 Secondary outcomes 6

4.3 Exploratory study endpoints 6

5. Statistical Analysis Methods 6

5.1 General Principle 6

5.2 Analysis Data Sets 6

5.2.1 Full Analysis Set (FAS) 6

5.2.2 Safety Set (SS) 6

5.3 Demographic and Baseline Characteristics 6

5.4 Efficacy Assessment 6

5.4.1 Primary Efficacy Assessment 7

5.4.2 Secondary Efficacy Endpoints 7

5.4.3 Exploratory Endpoints 7

1. Introduction

Triple-negative breast cancer, as a poor prognosis of refractory breast cancer, has still taken chemotherapy as the main treatment for many years. At present, preclinical studies have shown that immune combined with anti-angiogenic drugs can significantly improve anti-tumor activity, and clinical studies have also shown that Camrelizumab combined with anti-angiogenic drugs plus chemotherapy has good efficacy in the treatment of advanced TNBC with controllable safety. However, for the neoadjuvant treatment of early breast cancer, there is no research data of similar treatment at home and abroad.

Therefore, on the basis of previous studies, clinical studies of PD-1 immunotherapy combined with apatinib and neoadjuvant chemotherapy for triple-negative breast cancer are considered, aiming to explore more optimal treatment options for such patients.

This statistical analysis plan (SAP) will give more detailed descriptions of the endpoints in the study and the corresponding analyse.

2. Study design

This study is a single arm, prospective phase II clinical study scheduled to enroll 35 screened eligible subjects receiving 8 cycles of Camrelizumab plus apatinib plus chemotherapy neoadjuvant therapy.

In this study, the screening period shall not exceed 28 days, and the subjects who pass after completinsg the screening examination and assessment shall enter the study treatment period. Subject will be administered for 72h after enrollment, 1-4 cycles every 4 weeks, 5-8 cycles every 2 weeks until the end of the course or disease progression, intolerable toxicity, subject withdrawal of consent, or the investigator.

The overall design of the study is as follows:

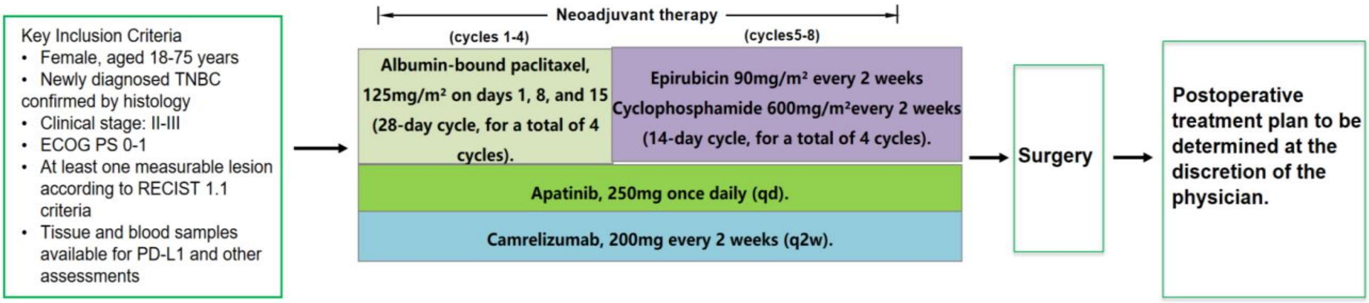

2.1 Sample size calculation

This study is a single arm design, and according to the study data of the KEYNOTE-522 and IMpassion031 trial treatment regimen, the tpCR rate of Camrelizumab+ apatinib + chemotherapy regimen in early TNBC. Using NCSS PASS 15 (LLC. Kaysville,Utah,USA,ncss.com/software/pass), the 95% confidence interval of tpCR rate for the approximately 31 subjects was [44%, 78%]. Considering the 10% shedding rate, a total of 35 subjects will be enrolled in this study.

3. Aims and objectives

To evaluate the efficacy of Camrelizumab combined with apatinib plus chemotherapy in neoadjuvant treatment of triple negative breast cancer.

4. Outcomes

4.1 Primary outcome

To evaluate the pathological complete response rate (tpCR) (ypT0 /is ypN0) of Camrelizumab plus apatinib plus chemotherapy for neoadjuvant treatment of triple-negative breast cancer（TNBC）.

The primary analysis will be conducted for the primary efficacy measure, the investigator-assessed tpCR.

4.2 Secondary outcomes

Breast pathological complete response (bpCR) (ypT0/is)
Objective Response Rate (ORR),
Event-Free Survival (EFS), and
Invasive Disease-Free Survival (iDFS).The incidence and severity grades of adverse events (AEs) and serious adverse events (SAEs) will be summarized and categorized according to NCI-CTCAE v5.0

4.3 Exploratory study endpoints

Potential predictive biomarker parameters related to efficacy (e. g., pCR, nonpCR) in peripheral blood and tumor tissue specimens, including but not limited to PD-L1, CTCs, TILs, CD4 / CD8, Tregs, BRCA 1 / 2, and PI3K / AKT / mTOR.

5. Statistical Analysis Methods

5.1 General Principle

Statistical analyses will be performed Using NCSS PASS 15 statistical software (LLC. Kaysville, Utah, USA, ncss.com/ software/pass).Continuous data will be summarized descriptively using mean, standard deviation (SD), median, maximum, and minimum values. Categorical and ordinal data will be described using frequencies and percentages.
In general, the maximum and minimum values will maintain the same number of decimal places as in the raw data; means and medians will be reported with one additional decimal place compared to raw data; standard deviations will be reported with two additional decimal places; percentages will be presented with one decimal place, and percentages will not be displayed when the count is zero. Confidence intervals will be presented with two decimal places. Changes from baseline in categorical variables will be summarized using cross-tabulation tables.

5.2 Analysis Data Sets

5.2.1 Full Analysis Set (FAS)

The Full Analysis Set will include all enrolled subjects who received at least one dose of the study treatment. Subjects may be excluded from the FAS if they meet any of the following criteria: failure to meet key inclusion criteria, did not receive any dose of study drug, or had no post-enrollment data available. Analyses of demographic characteristics, baseline data, and efficacy endpoints will be based on the FAS.
For patients with missing genomic testing data, appropriate handling will be implemented (e.g., exclusion from specific analyses or application of appropriate imputation or modeling strategies)

5.2.2 Safety Set (SS)

The Safety Set will consist of all subjects who received at least one dose of study treatment and have post-treatment safety evaluation data available. This set will be used for all safety analyses.

5.3 Demographic and Baseline Characteristics

Baseline is defined as the last non-missing assessment performed prior to the first dose of study treatment.Baseline characteristics will primarily include the following demographic and clinical parameters:General demographic variables (e.g., age, sex)，ECOG performance status，Menopausal status，HER2 status score，Ki-67 expression level，Clinical T stage，Clinical N stage，Overall clinical stage，PD-L1 status.Descriptive statistics will be applied based on data type:Categorical and ordinal variables: summarized using frequency counts and percentages (n, %).Continuous variables: summarized using the number of observations (n), mean, SD, median, minimum, and maximum.

5.4 Efficacy Assessment

Efficacy will be evaluated based on primary and secondary endpoints

5.4.1 Primary Efficacy Assessment

The primary efficacy endpoint is the total pathological complete response (tpCR), defined as the absence of invasive cancer in both breast and axillary lymph nodes following neoadjuvant therapy, i.e., ypT0/is and ypN0, as determined by local pathological evaluation.
The tpCR rate will be calculated as the proportion of patients achieving tpCR among all patients in the FAS. The point estimate of tpCR rate will be presented along with its corresponding 95% confidence interval (CI), computed using the Clopper-Pearson exact method.

5.4.2 Secondary Efficacy Endpoints

The secondary efficacy endpoints include:
(1) Breast Pathological Complete Response (bPCR):
Defined as the absence of invasive cancer in the breast (ypT0/is), regardless of nodal status. The bPCR rate will be calculated as the proportion of patients achieving bPCR among those in the FAS, along with the corresponding 95% CI calculated using the Clopper-Pearson exact method.
(2) Objective Response Rate (ORR):
Defined as the proportion of patients with a best overall response of complete response (CR) or partial response (PR) according to RECIST v1.1 (and/or iRECIST where applicable), as assessed by the Independent Review Committee (IRC) and investigators. The ORR will be summarized with 95% CI using the Clopper-Pearson method. Subgroup comparisons may be conducted using Chi-square or Fisher's exact tests.
(3) Event-Free Survival (EFS):
Defined as the time from randomization to the first documented occurrence of disease progression, local or distant recurrence, secondary malignancy, or death from any cause. EFS will be analyzed using Kaplan-Meier estimates, with median EFS and 95% CI reported. Between-group comparisons will be conducted using the log-rank test, and Cox proportional hazards models will be used to estimate hazard ratios and 95% CIs.
(4) Invasive Disease-Free Survival (iDFS):
Defined as the time from surgery to the first occurrence of invasive recurrence (local/regional/distant), secondary primary cancer, or death. iDFS will also be estimated using Kaplan-Meier analysis, and statistical comparisons will follow the same methods as for EFS.
(5) AEs and SAEs will be graded according to NCI-CTCAE version 5.0.
Descriptive statistics (frequency and percentage) will be used to summarize:

- The overall incidence of AEs and Immune-related adverse events (irAEs)
- AEs by system organ class (SOC) and preferred term (PT)
- Treatment-related Aes： Any grade and Grade ≥3

5.4.3 Exploratory Endpoints

The exploratory endpoints of this study include:
(1) Genomic profiling of baseline tumor tissues
Whole-exome sequencing (WES) will be used to identify somatic mutations, copy number variations (CNVs), and tumor mutational burden (TMB) in baseline tumor samples.
Associations between genomic alterations (e.g., HRD score, BRCA mutation) and treatment response (tpCR/non-tpCR) will be explored.
(2) Dynamic changes in plasma immune proteomics before and after NAT
Differentially expressed proteins will be identified from Olink proteomic data.
Longitudinal changes will be analyzed to identify immune-related biomarkers predictive of response to NAT.
ROC analysis will be used to evaluate predictive value of candidate biomarkers.
