# Supplementary material for: Exploratory phase II trial of an anti-PD-1 antibody camrelizumab combined with a VEGFR-2 inhibitor apatinib and chemotherapy as a neoadjuvant therapy for triple-negative breast cancer (NeoPanDa03): efficacy, safety and biomarker analysis
Source: Signal Transduct Target Ther. 2025 Jul 21;10:237. doi: 10.1038/s41392-025-02337-1 (PMC12279984; doi:10.1038/s41392-025-02337-1)
Supplement: Supplementary file 3 — study protocol [file 41392_2025_2337_MOESM3_ESM.docx]

**Biomedical ethics research protocol**

**(Interventional clinical study)**

Exploratory Phase ll Trial of Camrelizumab Combined with Apatinib and Chemotherapy as Neoadjuvant Therapy for Triple-Negative Breast Cancer (NeoPanDa03): efficacy, safety and biomarker analysis

Research unit: West China Hospital, Sichuan University

Project leader: Ting Luo

Department: Head and Neck Oncology Department

Contact number: 18980606230

Group leader unit: West China Hospital, Sichuan University

Study years: December 2021 —— June 2023

Version number: V2.0

Version date: September 08,2023

**Clinical Trial Team Leader Unit**

| Research unit information | |
| --- | --- |
| Name | West China Hospital, Sichuan University |
| Location | No.37, Guoxue Lane, Wuhou District, Chengdu city, Sichuan Province |
| Principal investigator | Ting Luo |

**scenario summary**

| **research topic** | Exploratory Phase II Trial of Camrelizumab Combined with Apatinib and Chemotherapy as Neoadjuvant Therapy for Triple-Negative Breast Cancer (NeoPanDa03): efficacy, safety and biomarker analysis |
| --- | --- |
| **Scheme number** | MA-BC-II-026 |
| **version number** | V1.0 |
| **Version date** | On April 30,2021 |
| **research on drug** | Camrelizumab, apatinib, albumin paclitaxel |
| **Research unit** | West China Hospital, Sichuan University |
| **Principal investigator** | Ting Luo |
| **Research basis** | Previous studies have shown that chemotherapy combined with immunotherapy can improve the anti-tumor efficacy, and chemotherapy has the effect of activating the tumor immune circulation. IMpassion130 And KEYNOTE-355 showed that combination chemotherapy with atezolizumab or pembrolizumab had significant efficacy and controllable safety in the first-line treatment of metastatic triple-negative breast cancer (mTNBC).  KEYNOTE522 The study showed that pembrolizumab (Carboplatin-based paclitaxel increased pT0/Tis ypN0) by 13.6% (51.2% vs 64.8%).  IMpassion031 The study showed that for neoadjuvant therapy in TNBC patients, atezolizumab + chemotherapy showed a statistically significant pCR rate compared with placebo + chemotherapy, and pCR rate increased by 16.5% (57.6% vs 41.1%, p=0.0044). |
| **purpose of research** | **Main research objectives**   - To evaluate the efficacy of Camrelizumab combined with apatinib plus chemotherapy in neoadjuvant treatment of triple negative breast cancer   **Secondary research purpose**   - To evaluate the efficacy and safety of Camrelizumab combined with apatinib plus chemotherapy in neoadjuvant treatment of triple-negative breast cancer   **Exploratory study objectives**   - To explore potential predictive biomarkers related to efficacy in peripheral blood and tumor specimens. |
| **Study end point** | **Main study endpoints**   - To evaluate the pathological complete response rate (tpCR) (ypT0 /is ypN0) of Camrelizumab plus apatinib plus chemotherapy for neoadjuvant treatment of triple-negative breast cancer.   **Secondary study endpoints**   - To evaluate the efficacy of Camrelizumab combined with apatinib + chemotherapy in neoadjuvant treatment of triple negative breast cancer: bpCR (ypT0/is), RCB-0 / I, ORR, EFS, iDFS; - To evaluate the safety of Camrelizumab plus apatinib + chemotherapy in neoadjuvant treatment in triple-negative breast cancer: adverse events (AEs), serious adverse events (SAEs), abnormal laboratory tests, etc.   **Exploratory study endpoints**   - Potential predictive biomarker parameters related to efficacy (e. g., pCR, RCB-0 / I, EFS, iDFS, etc.) in peripheral blood and tumor tissue specimens, including but not limited to PD-L1, CTCs, TILs, CD4 / CD8, Tregs, BRCA 1 / 2, and PI3K / AKT / mTOR. |
| **study population** | Patients with histopathologically confirmed early (locally advanced stage II-III) primary triple-negative breast cancer with negative ER (IHC ER positive <1%) and negative PR (IHC PR positive <1%) and HER 2 negative (IHC 0 / 1 + or IHC 2 + but ISH-). |
| **research design** | This study is a single arm, prospective phase II clinical study scheduled to enroll 35 screened eligible subjects receiving 8 cycles of Camrelizumab plus apatinib plus chemotherapy neoadjuvant therapy. The overall design is shown as follows:  In this study, the screening period shall not exceed 28 days, and the subjects who pass after completing the screening examination and assessment shall enter the study treatment period. Subject will be administered for 72h after enrollment, 1-4 cycles every 4 weeks, 5-8 cycles every 2 weeks until the end of the course or disease progression, intolerable toxicity, subject withdrawal of consent, or the investigator. |
| **The way of administration** | The following drug use regimen was recommended for initial treatment in all trial subjects:  Camrelizumab Injection:  200 mg every 2 weeks, Cycles 1-4 on Days 1,15, Cycle 5-8 for approximately 30min (no less than 20min, no more than 60min). No dose increase or decrease is allowed, delay / interruption, delay / interruption maximum 12 weeks, calculated from the last dose, otherwise treatment was terminated.  Apatinib mesylate tablets:  250mg qd orally, about half an hour after meals (the time of daily medication should be the same as far as possible), with warm boiled water, take 8 consecutive treatment cycles, if missing is not allowed to fill. No dose increase or downregulation is allowed, delay / interruption is 28 days, calculated from the last dose, otherwise treatment was terminated.  Albumin-Paclitaxel Injection:  Every 4 weeks is one treatment cycle, cycles 1-4 for 4 cycles; 100mg / m2 albumin on days 1,8,15, IV infusion, approximately 30min. Dose down and delayed dose were allowed, with a maximum of 3 weeks, calculated from the last dose time, otherwise treatment was terminated.  **epirubicin:**  Every 2 weeks was a treatment cycle, cycles 5-8 for 4 cycles; 90mg / m2 epirubicin, intravenous infusion, for approximately 30min. Dose down and delayed dose were allowed, with a maximum of 3 weeks, calculated from the last dose time, otherwise treatment was terminated.  **cyclophosphamide:**  Every 2 weeks was one treatment cycle, cycles 5-8 for 4 cycles; 600mg / m2 cyclophosphamide was administered on day 1 of each cycle, with intravenous infusion of approximately 30min. Dose down and delayed dose were allowed, with a maximum of 3 weeks, calculated from the last dose time, otherwise treatment was terminated.  **Order of the combination of drug administration:**  During the combination of Camrelizumab and chemotherapy, Camrelizumab was given by intravenous infusion of at least 30min intervals before intravenous infusion of chemotherapeutic agents.  The subject should continue study treatment until the end of the course, disease progression, toxicity intolerance, active request for study treatment termination or withdrawal from the study, or the investigator determines the subject to withdraw from the study. |
| **Enrollment criteria** | Subjects must meet all of the following inclusion criteria to be enrolled in this trial:   1. Women aged 18 years and 75 years old, treated for primary breast cancer; 2. Histopathologically confirmed early or locally advanced triple-negative invasive breast cancer, as defined by the latest ASCO / CAP guidelines, with the following conditions:  - Pathological types were triple negative, specifically: ER negative: IHC <1%, PR negative: IHC <1%, HER 2 negative: IHC 0 / 1 + or IHC2 + but ISH negative; - Tumor stage: stage II-III;  1. ECOG score of 0~1; 2. The expected survival period is not less than 3 months; 3. At least one measurable lesion was present as per the RECIST 1.1 criteria; 4. The functional level of the organs must meet the following requirements:   1) routine blood test  • Neutrophil count (ANC)) 1.5109 / L (no 14 days prior to the first dose of the study);  White blood cell count (WBC) 3.0109 / L and 15109 / L;  Lymphocyte count (LC) 0.5109 / L;  • Platelet count (PLT) 100109 / L (no blood transfusion within 14 days prior to the first dose of the study);  Hemoglobin (Hb): 90 g / L;  2) Blood biochemical  • TBIL≤1.5×ULN；  ALT and AST 2.5 ULN;  • ALP≤2.5 ULN  • BUN and Cr 1.5 ULN with a creatinine clearance of 50 mL/min (Cockcroft-Gault formula);  INR and APTT 1.5 ULN (no anticoagulant therapy)  • Upper limit of normal (TSH) thyroid-stimulating hormone (ULN); abnormal T3 and T4 levels, normal T3 and T4 levels can be included;  3) heart function  Heart color ultrasound: LVEF 50%;  12-lead ECG: 1 ° QT interval <470 ms.  7. Tissue samples for evaluation of PD-L1 (at least 5 white slices of tissue specimens);  8. Contraception:  Female fertile subjects are required to have a negative serum pregnancy test within 72 hours before the first dose and agree to use a medically approved, highly effective method of contraception during the study and within 90 days after the last administration of study drug.  After consultation with the subject, the investigator or his / her designee selected the appropriate contraceptive measures for him and her partner from the following contraceptive methods and confirmed that the subject had known how to correctly and consistently use the contraceptive method. At the time points listed in the flow sheet, the investigator will inform the subject of the need for continuous, correct contraception. In addition, the subject should be informed of the cessation of the chosen contraception or of suspected or confirmed pregnancy.  Efficient contraception is used correctly alone or combined with other methods, with a failure rate of less than 1% per year.  Including the following types:  1) Correct placed was placed placed.  2) Condoms are simultaneously combined with topical spermicide (i. e. foam, gel, film, cream, or suppositories).  3) Bilateral tubal ligation / bilateral salpingectomy or bilateral tubal occlusion surgery (the occlusion surgery has been proved to be effective by the relevant instruments).  4) Men were sterilized by vasectomy.  9. voluntarily joined the study, signed informed consent, had good compliance and willingness to cooperate with follow-up. |
| **Exclusion criteria** | Not to be included in any of the following conditions:  1.Tumor-related symptoms and treatment:   1. Patients with metastatic breast cancer or bilateral breast cancer; 2. Inflammatory breast cancer patients; 3. Having received any anti-tumor therapy within 12 months before signing the informed consent form, including targeted therapy, chemotherapy, radiotherapy, endocrine therapy, immunotherapy, biological therapy or tumor embolization; 4. Prior treatment with PD-1 / PD-L1 antibody, CTLA-4 antibody, or other therapies against PD-1 / PD-L1 and / or VE GF R inhibitors.   2.Concurrent disease / medical history   1. Any other active malignancies within 5 years prior to signing the informed consent. Curable localized tumors, such as skin basal cell carcinoma, skin squamous cell carcinoma, superficial bladder cancer, cervical carcinoma in situ, can be enrolled; 2. Have undergone a major surgical procedure unrelated to breast cancer within the 4 weeks before enrollment, or the patient has not fully recovered from such surgical procedure (tissue biopsy for diagnosis and central venous catheter placement through peripheral venous puncture [PICC] are allowed); 3. Subjects with any known or suspected autoimmune disease except: hypothyroidism due to autoimmune thyroiditis requiring only hormone replacement therapy; stable type I diabetes with blood glucose control; 4. Hypertension that could not be well controlled with antihypertensive medication (systolic> 140 mmHg or diastolic> 90 mmHg); within 6 months prior to enrollment, the following conditions occurred: myocardial infarction, severe / unstable angina, grade NYHA 2 or above cardiac dysfunction, clinically significant supraventricular or ventricular arrhythmia, and symptomatic congestive heart failure; 5. Presence of interstitial lung disease, noninfectious pneumonia, or uncontrollable systemic diseases (such as: diabetes mellitus, pulmonary fibrosis, and acute pneumonia, etc.); 6. History of live attenuated vaccination within 28 days before the first study administration or vaccination during the expected study; 7. Human immunodeficiency virus (HIV) infection or known acquired immunodeficiency syndrome (AIDS); active hepatitis B (defined as positive test for hepatitis B virus surface antigen and positive for HBV-DNA); hepatitis C (defined as HCV-RNA above the lower limit of detection of the analytical method) or combined co-infection with hepatitis B and hepatitis C; autoimmune hepatitis; 8. Severe infection within 4 weeks prior to first dose, including, but not limited to bacteremia, severe pneumonia requiring hospitalization; or active infection with CTCAE grade 2 within 2 weeks prior to first dose, or unexplained fever> 38.5°C during screening / before first dose (according to the investigator); evidence of active TB infection within 1 year prior to administration; 9. Subjects with previous or ready allogeneic bone marrow transplantation or solid organ transplantation; 10. Peripheral neuropathy grade 2; 11. There is a clinically significant intestinal obstruction; 12. Arteriovenous thrombotic events occurring within 3 months before enrollment, such as cerebrovascular accidents (including temporary ischemic attacks), deep venous thrombosis and pulmonary embolism; 13. Subjects with hemoptysis within 2 months before enrollment and a maximum daily hemoptysis of 2 ~ 2.5 mL. Within 3 months before enrollment, there were significant bleeding symptoms or clear bleeding tendency, such as gastrointestinal bleeding, hemorrhagic gastric ulcer, baseline fecal occult blood + + or above, or known genetic or acquired bleeding and thrombosis tendency, such as hemophilia, coagulation skills disorder, thrombocytopenia, hypersplenism, etc.; 14. Abnormal coagulation (INR> 1.5 or APTT> 1.5 ULN), with bleeding tendency or being on thrombolysis or requiring long-term anticoagulation with warfarin or heparin, or requiring long-term antiplatelet therapy (aspirin 300 mg / day or clopidogrel 75 mg / day).   3.Study treatment-related   1. Subjects who had received systemic immune stimulants (including but not limited to interferon or interleukin-2, including those in the clinical study phase) within 4 weeks prior to the first dose; 2. Subjects who had received systemic immunosuppressive agent therapy (including but not limited to glucocorticoids, azathioprine, methotrexate, thalidomide, anti-tumor factor agents) within 2 weeks prior to the first dose. Not including nasal spray and inhaled corticosteroids or physiological doses of systemic steroid hormones (i. e., no more than 10 mg/d of prednisone or other corticophysiological doses of equivalent drugs); 3. Known allergy to study drug or any of its excipients; or a severe allergic reaction to other monoclonal antibodies.   4.Female patients during pregnancy and lactation, female patients with fertility and positive baseline pregnancy test, or female patients of childbearing age who are not willing to take effective contraceptive measures throughout the trial;  5.A clear prior history of neurological or psychiatric disorders, including epilepsy or dementia, and a known history of psychotropic substance abuse, alcohol abuse, or drug use;  6.The patient was not considered fit by the investigator to participate in any other circumstances of the study. |
| **Data analysis and statistical methods** | - **Sample size estimation**   This study is a single arm design, and according to the study data of the KEYNOTE-522 and IMpassion031 trial treatment regimen, the tpCR rate of Camrelizumab + apatinib + chemotherapy regimen in early TNBC. Using NCSS PASS 15 (LLC. Kaysville,Utah,USA,ncss.com/ software / pass), the 95% confidence interval of tpCR rate for the approximately 31 subjects was [44%, 78%]. Considering the 10% shedding rate, a total of 35 subjects will be enrolled in this study.   - **efficiency analysis**   The analysis of the efficacy endpoints of this study will be based on the FAS set and PPS set and will dominate the results of the FAS set.  Analysis will be performed using SAS ® System version 9.4 or above, and all tables, pictures and lists will be generated in an RTF file.  For descriptive statistical analysis, unless specified, number of subjects (n) versus percentage (%) and missing versus percentage for subtype data will be provided. For continuous data, the number of non-missing subjects (n), arithmetic mean, standard deviation, median, minimum, maximum will be provided.  For the efficacy endpoint of the second classification (including pCR, RCB-0 / I, ORR), the number and percentage of subjects under each classification will be summarized and the 95% confidence interval calculated using the Clopper-Pearson method.  For efficacy endpoints (including EFS, iDFS), the number and percentage of subjects with events and censoring were summarized and summary statistics were pooled for event types and cause of censoring.  The 3-and 5-year EFS, iDFS rates will be estimated using the Kaplan-Meier method and survival plots will be drawn. The 95% confidence interval for survival was calculated using the log (-log) method.   - **safety analysis**   All safety analyses will be based on the SS set. All adverse events will be graded according to NCI-CTCAE 5.0, with descriptive statistics: AE, SAE, Grade 3 AE, Grade 3 SAE, drug-related AE, AE, dose adjustment, and AE, treatment termination. For AEs of special concern, frequency and percentage were summarized, including the mean, standard deviation, median, maximum and minimum.  Laboratory test results, vital signs, and electrocardiogram data will be analyzed from baseline and after baseline.   - **exploratory analysis**   Correlation analysis of pCR, ORR, RCB-0 / I, EFS and iDFS according to the biomarker condition. |
| **Research progress** | Expected enrollment time of the first subject: September 2021  Expected enrollment time of the last subject: June 2022 |

**Test process table**

| **project** | **Screening period**  **(D-28 to D-1)** | **Neoadjuvant treatment period** | | | | **End of treatment / withdrawal from study / surgery** | **^Safety follow-up for the [34]^** | **Survival follow-up**  **(3 months / time)** |
| --- | --- | --- | --- | --- | --- | --- | --- | --- |
|  |  | **C1-C4** | | | **C5-C8** |  |  |  |
|  |  | ^D1[2]^ | **D8(±3)** | **D15(±3)** | **D1(±3)** |  |  |  |
| ^Informed consent was signed [1]^ | √ |  |  |  |  |  |  |  |
| ^Demographic data [3]^ | √ |  |  |  |  |  |  |  |
| ^Tumor diagnosis [4]^ | √ |  |  |  |  |  |  |  |
| ^anamnesis [5]^ | √ |  |  |  |  |  |  |  |
| ^History of previous antitumor therapy [6]^ | √ |  |  |  |  |  |  |  |
| ^physical examination [7]^ | √ |  |  |  |  | √ |  |  |
| stature | √ |  |  |  |  |  |  |  |
| ^Weight [8]^ | √ | √ | √ | √ | √ | √ | √ |  |
| ^vital sign [9]^ | √ | √ | √ | √ | √ | √ | √ |  |
| ^ECOG grade [10]^ | √ | √ |  |  | √ | √ | √ |  |
| ^routine blood test [11]^ | Vacation (within 7 days) | √ | √ | √ | √ | √ | √ |  |
| ^Blood biochemical [12]^ | Vacation (within 7 days) | √ | √ | √ | √ | √ | √ |  |
| ^Urinary routine [13]^ | Vacation (within 7 days) | √ |  |  | √ | √ | √ |  |
| ^Fecal occult blood [14]^ | Vacation (within 7 days) | √ |  |  | √ | √ | √ |  |
| ^Coagulation function [15]^ | Vacation (within 7 days) | √ |  |  | √ | √ | √ |  |
| ^Thyroid function examination [16]^ | Vacation (within 7 days) | √ |  |  | √ | √ | √ |  |
| ^HIV / HBV / HCV virus examination [17]^ | √ |  |  |  |  |  |  |  |
| ^Pregnancy test [18]^ | Vacation (within 3 days) |  |  |  |  | √ |  |  |
| ^12-lead ECG [19]^ | Vacation (within 7 days) | √ |  |  | √ | √ | √ |  |
| ^Echocardiogram [20]^ | √ |  |  |  | (Once at C5D1) | √(Preoperative) |  |  |
| Imaging examination (breast) [21] | √ | It was evaluated every two cycles | | | | √ |  |  |
| ^Imaging examination (sites other than the breast) [22]^ | √ |  |  |  |  | √(Preoperative) |  |  |
| The location of the primary tumor lesions marks skin tattoos and / or surgical metal clips | √ |  |  |  |  |  |  |  |
| Camrelizumab ^[24]^ |  | √ |  | √ | √ |  |  |  |
| ^Apatinib [25]^ |  | Dosed daily and administered continuously orally | | | |  |  |  |
| ^Aalbumin paclitaxel [26]^ |  | √ | √ | √ |  |  |  |  |
| ^Epirubicin plus cyclophosphamide [27]^ |  |  |  |  | √ |  |  |  |
| ^Assessment of the pathological response rate [28]^ |  |  |  |  |  | √(postoperative) |  |  |
| ^Tumor tissue specimens [29]^ | √ |  |  |  |  | √(postoperative) |  |  |
| ^Peripheral blood specimens [30]^ | √ |  |  |  | (Once at C5D1) | (Pre and Post) |  |  |
| ^Adverse event [31]^ | √ | | | | | | |  |
| ^Concomitant medication / concomitant therapy [32]^ | √ | | | | | | |  |
| ^Survival follow-up [33]^ |  |  |  |  |  |  |  | √ |

Note: All visits and assessments should be performed within ± 3 days of the prescribed visit. All tests should be completed within 3 days before each dose and the administration. If the implementation time required by the test protocol meets holidays or weekends, it will be postponed to the most recent date. In addition to the inspection items and time points listed in the table, the investigator may add the items necessary for inspection at any time, and the inspection results shall be recorded in the eCRF.

[1] Written informed consent must be obtained before any clinical study procedure, except for tumor imaging, tumor tissue biopsy, pathological diagnosis, and genetic testing within the prescribed time limit before the first medication. If the subject has the site within the specified window period before signing the ICF, no repeat testing will be required during the screening period. This study allows previously failed subjects to re-screen, re-sign the informed consent form and re-register to obtain a new subject number.

[2] Before the first dose, if all laboratory test items have been checked during the screening period and within 7 days, no retest is required before the first dose.

[3] Including the date of birth, age, gender, ethnicity, etc.

[4] The primary site of the subject must be recorded before enrollment, and the pathological histological diagnosis results, including pathological specimen type and acquisition method, pathological classification and immunohistochemical expression (ER, PR, HER 2 expression), histological grade, clinical stage and TNM stage, etc. If there is suspected lymph node metastasis at baseline, fine needle aspiration is required.

[5] Including all previous diseases and current active diseases, autoimmune diseases occurring at any time should be recorded.

[6] All previous anti-tumor treatments (systemic and local medication, neoadjuvant / adjuvant chemotherapy, radiotherapy, surgical therapy, physical therapy, etc.), as well as the specific regimen, efficacy evaluation, start and ending time of these treatments must be recorded.

[7] It includes the evaluation of general condition, head and neck (including thyroid), chest (including heart, lung), abdomen (liver, bile, spleen, kidney), limbs, skin, lymph nodes, genito-urinary system, musculoskeletal, nervous system and mental state. Abnormalities at baseline were recorded in the comprehensive medical history and baseline eCRF. Clinically significant abnormalities were recorded in the adverse event eCRF. Check once during the screening period and before surgery (if not performed within the previous 7 days); add any associated discomfort symptoms at any time.

[8] Body weight will be recorded during the screening period, the day of each dose, end of treatment or preoperative and safety follow-up.

[9] Vital signs include temperature, pulse, respiratory rate and blood pressure, screening period, day of each dose, end of treatment or preoperative, and safety follow-up. During the treatment period, one vital sign examination was performed within 30 min before each drug infusion and after 30min, not more than 60 min. If you have any relevant discomfort symptoms, it can be added at any time.

[10] ECOG: Screening period, within 3 days before each cycle, end of treatment, or preoperative, safety follow-up. If you have any relevant discomfort symptoms, it can be added at any time.

[11] Blood routine: including complete blood cell count and classification. The screening period was performed within 7 days before the first dose. Test within 3 days prior to each dose during the study (except the first dose), end of treatment or preoperative, safety follow-up, and additional tests if necessary.

[12] Blood biochemistry: including ALT, AST, γ -GT, TBIL, DBIL, ALP, BUN, TP, ALB, LDH, Cr, GLU, K +, Na +, Ca 2 +, Mg2 +, Cl-. The screening period was performed within 7 days before the first dose. Test within 3 days prior to each dose during the study (except the first dose), end of treatment or pre-operative, safety follow-up, and additional tests if necessary.

[13] Urine routine: including urine white blood cells, urine red blood cells, urine protein. The screening period was performed within 7 days before the first dose. If urinary protein 2 +, additional testing for 24-hour urinary protein quantification is required, and subjects with 1g of 24-hour urinary protein quantification are not recommended. Test within 3 days prior to each cycle dose during the study (except for the first dose during the first course). End of treatment or preoperative or safety follow-up is required once.

[14] fecal occult blood: within 7 days before the first dose. If positive, it must be reviewed for stool occult blood +. The investigator performed gastroenteroscopy according to clinical needs to exclude gastrointestinal bleeding. Test within 3 days prior to each cycle dose (except for the first dose of the first course). End of treatment or preoperative or safety follow-up is required once. If necessary, the investigator may add additional tests.

[15] Coagulation function: including international normalized ratio INR, activated partial thromboplastin time APTT, fibrinogen FIB, and thrombin time TT; if international normalized ratio INR is not available, then prothrombin time PT is used. One test within 7 days before the first dose, within 3 days before each cycle (except the first dose of the first course), end of treatment or preoperative, and complete follow-up, with additional tests added if necessary.

[16] Thyroid function test: including serum thyroid stimulating hormone (TSH), free triiodothyronine (FT3), free thyroxine (FT4), such as FT3, FT4 is not available, can be replaced by T3 and T4. The screening period was performed within 7 days before the first dose. Test within 3 days prior to each cycle dose during the study (except for the first dose). End of treatment or preoperative or safety follow-up is required once.

[17] Participants will be tested for HIV (HIV), hepatitis B (HBV) or hepatitis C (HCV). Hepatitis B virus testing includes: HBsAg, HBsAb, HBcAb, HBeAg, and HBeAb. If HBsAg is positive, HBV-DNA testing should be tested. Hepatitis C test: HCV-Ab, if HCV-Ab is positive, HCV-RNA test. If hepatitis carriers participate in the study, ask the investigator to arrange antiviral treatment at their discretion, and review the viral load (HBV-DNA or HCV-RNA) at 6 weeks intervals during the study, and, if necessary, as clinically indicated.

[18] Female subjects of childbearing age should have a serum pregnancy test within 72 hours before the first dose of medication in the screening period and at the end of treatment. If necessary, the investigator may add additional examinations.

[19] The 12-lead ECG should be attentive to heart rate, QT, QTc, and P-R interval, performed within 7 days prior to the first dose during the screening period. If you have chest pain, palpitations or other cardiac symptoms, do it at any time. A 12-lead ECG was required within 3 days before each cycle of the study (within 7 days before the first dose of the screening period) and for end of treatment or preoperative, safety follow-up.

[20] Echocardiography should include LVEF assessment, performed within 28 days before the first dose during the screening period, once within 3 days before cycle 5 administration, and once before the end of treatment or surgery. Furthermore, when examined when clinically indicated.

[21] Breast imaging assessment: during the screening period and preoperative, breast examination, mammography, breast ultrasound and breast MRI, breast ultrasound or MRI assessment every 2 cycles during neoadjuvant treatment, during treatment, necessary imaging tests can be added when other lesions are suspected.

[22] Radiological evaluation of sites outside the breast: regular clinical tumor imaging evaluation during screening and after treatment according to RECIST v1.1 criteria. During the screening period, the subjects should be evaluated as far as possible, and complete imaging examinations on the head, neck, chest, abdomen, pelvic and bone tissue, CT / MRI, chest, upper abdomen, pelvic CT scan, whole body bone scan) according to the principle of diagnosis and treatment, if the routine tumor imaging evaluation (within 42 days before 42 days before the first dose), it is not necessary to repeat during the screening period. During study treatment, the investigator may perform the necessary imaging tests according to the subject.

[23] Before neoadjuvant therapy, standard methods (e. g., skin tattoos or surgical metal clips).

[24] Carlizumab IV: fixed dose, 200 mg every 2 weeks, 200 mg, IV, days 1,15, cycles 5-8, allowing interruption or delay of 12 weeks, calculated from the last dose, otherwise treatment was terminated. The time window for each dose was calculated from the date of the first dose of each cycle, and the D15 dose delay was generally not exceeding 3 days. In case of a delay (3 days), the next dose window, the next cycle dose window, as planned. If D15 administration is delayed by more than 3 days, no dose supplementation is recommended for this cycle, and the next cycle administration time window continues as planned.

[25] Oral administration of apatinib: 250mg qd orally, about half an hour after meals (the daily medication time should be the same as much as possible), with warm water, if missing, will not be taken. No dose increase or downregulation is allowed, delay / interruption is 28 days, calculated from the last dose, otherwise treatment was terminated. Except for imaging, all tests should be completed within 3 days before each dose before continuing medication after safety assessment by the investigator.

[26] Intravenous infusion of albumin paclitaxel: the time interval should be no less than 7 days, every 4 weeks as a treatment cycle. Dose down and delayed dose were allowed, with a maximum of 3 weeks, calculated from the last dose time, otherwise treatment was terminated. From the time of first dose, the in-period delay dose window will generally not exceed 3 days. If there is a delay (3 days), the next medication time will be recalculated with the actual dose time, and the subsequent dose time window will remain 3 days. If D8 is delayed (> 3 days), skip directly to D15. If D15 administration is delayed for more than 3 days, the D15 administration is skip and the dose is not stopped in this cycle, and in the next cycle, the dose time of the next cycle will be the time window calculated by the first dose date of the cycle. Albumin paclitaxel and carlizumab were guaranteed to be administered on D1 and D15.

[27] IV administration of epirubicin combined with cyclophosphamide: The time dose interval of epirubicin combined with cyclophosphamide should be no less than 14 days for one cycle for every 2 weeks. From the time of first dose, the period generally generally from 3 days. If the next cycle is delayed (3 days), the subsequent dose time window remains unchanged. If the next cycle is delayed (> 3 days), the next dose time is recalculated with the actual dose time.

[28] Pathological response (tpCR) will be evaluated through tumor tissue specimens obtained during the operation, and all response assessments will be assessed by each study center according to the key points listed in Annex VI and 7.

[29] Pathological tissue specimens should be provided before and after enrollment, with paraffin blocks, or unstained tissue sections. No less than 10 white tablets are used for the detection of PD-L1 and other biomarkers, and fine needle puncture specimens are not accepted. Brush specimens, pleural effusion cell precipitation specimens, bone metastasis specimens and lavage specimens are not used for testing. All specimens should be verified by the pathology department of the participating center for this study.

[30] Peripheral blood samples: peripheral blood was collected once within 3 days before the first dose during the screening period, 3 days before cycle 5, and before and after the operation, and at least 7.5 mL at each time.

[31] Adverse events: Adverse events were observed and recorded according to NCI CTC AE v5.0 criteria and collected from the signing of informed consent to the end of safety follow-up. Only SAEs related to the study drug will be collected after the safety follow-up period. All adverse events should be traced to recovery or back to baseline, or stable disease or until grade 1 (CTCAE v5.0), or subject lost to follow-up or death.

[32] Concomitant / concomitant treatment: All concomitant medications from the 30 days prior to the first study medication to the end of the safe follow-up period. Concomitant medication records should include drug name, drug dose, route of administration, frequency of administration, purpose of administration, and start and end dates. If the subject initiated new systemic systemic anti-tumour therapy during safety follow-up, only concomitant medication / concomitant therapy for adverse events related to the study drug were recorded. Once the subject interrupted trial treatment, only the concomitant medication and concomitant treatments for new or unresolved adverse events related to trial treatment should be recorded.

[33] Survival follow-up: All subjects will undergo survival follow-up after completing surgery / early termination of treatment. From the date of procedure completion / early termination visit, every 3 months during the first year and every 6 months follow-up, call (or clinical follow-up) the subject, his family or local physician until disease progression or recurrence, loss to follow-up or until 5 years after the last subject is enrolled (whichever occurs first).

[34] Safety follow-up: 30 days (± 7 days) or 90 days (± 7 days) or new anti-tumor treatment, the first safety visit was 30 days (± 7 days) at the site. The second and third follow-ups are no longer checked for safety indicators and can be visited by telephone. Only all SAEs considered related to the study drug will be collected after the safety follow-up period.

abbreviation

| abbreviation | Full name of English | Full name of Chinese |
| --- | --- | --- |
| TNBC  12-Lead ECG | Triple-negative breast cancer  12-Lead electrocardiogram | Triple Negative Breast Cancer  12-lead ECG |
| AE | Adverse event | adverse event |
| ALP | Alkaline phosphatase | alkaline phosphatase |
| ALT | Alanine aminotransferase | Glutamate alanine aminotransferase |
| AST | Aspartate aminotransferase | Glutamate aspartate aminotransferase |
| ANC | Absolute neutrophil count | Neutrophil count was indicated |
| APTT  ADA | Activeated partial thromboplastin time  Anti-drug Antibody | Activated partial thromboplastin time  Anti-drug antibodies |
| BP | Blood pressure | blood pressure |
| BUN | Blood urea nitrogen | usea nitrogen |
| BOR | Best overall response | Best overall efficacy |
| BSC | Best supportive care | Optimal supportive care |
| Ca | Calcium | calcium |
| CD-ROM | Compact Disc Read-Only Memory | CD-ROM |
| CI | Confidence interval | credibility interval |
| CNS | Central nervous system | C. N. S |
| CR | complete response | complete remission |
| Cr | Creatinine | creatinine |
| CRF | Case report form | Case report form |
| Cl- | Blood chlorine | Blood chlorine |
| CT | computed tomography | Computerized X-ray tomography scan |
| CTCAE | Common Terminology Criteria for Adverse Events | Evaluation criteria for common adverse reaction events |
| DBIL | Direct bilirubin | bilirubin direct |
| DCR  CBR | disease control rate  Clinical benefit rate | Disease control rate  Clinical benefit rate |
| DMC | Data Monitoring Committee | The Data Monitoring Committee |
| DoR | Duration of response | Relieve duration |
| DNA | Deoxyribonucleic acid | deoxyribonucleic acid |
| EC | Ethics committee | Ethics Committee |
| ECG | Electrocardiogram | electrocardiogram |
| ECOG PS | Eastern Cooperative Oncology Group  Physical status score | Scoring criteria for physical strength status of the Eastern Cooperative Oncology Group |
| EGFR | Epidermal growth factor receptor | EGFR |
| eCRF | Electronic Case report form | Electronic medical record report form |
| EDC | Electroinc data collection | Clinical electronic data acquisition system |
| FDA | Food and Drug Administration | The US Food and Drug Administration |
| FT3 | Free triiodothyronine | Free triiodothyronine |
| FT4 | Free thyroxine | free thyroxine |
| FFPE | Formalin fixed paraffin embedded | Paraffin embedded tissue |
| GCP | Good Clinical Practice | Good Laboratory Practice for drug clinical studies |
| G-CSF | Granulocyte colony stimulating factor | Granulocyte-colony-stimulating factor |
| GLU | Blood glucose | blood sugar |
| GLU-U | Uglu urine glucose | glucose in urine |
| Hb | Hemoglobin | hemoglobin |
| HBV | Hepatitis B virus | hepatitis B virus |
| HbsAg | Hepatitis B surface antigen | Hepatitis B virus surface antigen |
| HbcAb | Hepatitis B core antibody | Hepatitis B virus core antibodies |
| HCV | Hepatitis C virus | HCV |
| HDL-C | High-density lipoproteincholesterol | Plasma with HDL cholesterol |
| HIV | Human immunodeficiency virus | Human immunodeficiency virus |
| HR | Hazard ratio | hazard ratio |
| IB | Investigator’s brochure | The researcher's manual |
| IBIL | Indirect bilirubin | indirect bilirubin |
| ICH | International Conference on Harmonization | Technical requirements for human drug registration IACC |
| irAE | Immune related adverse events | Immune-related adverse events |
| INR | International Normalized Ratio | international normalized ratio |
| IU | International unit | international unit |
| ITT | Intent to treat | intentional analysis |
| IRB | Institutional Review Board | The Institutional Review Board |
| IRC | Independent Review Committee | (Imaging) independent review committee |
| IHC | Immunohistochemistry | Immunohistochemical techniques |
| Ig | Immunoglobulin | immunoglobulin |
| IV | Intravenous | I.V |
| LLN | Lower limits of normal | lower limits of normal |
| LVEF | Left ventricular ejection fraction | Left ventricular ejection fraction |
| LEU | Leukocytes in urine | Urinary white blood cells |
| LDH | Lactate dehydrogenase | lactate dehydrogenase |
| LDL-C | Low-density lipoprotein cholesterol | Plasma with LDL cholesterol |
| LYMPH | Lymphocyte | leukomonocyte |
| K+ | Serum potassium | Blood potassium |
| KET | Urine acetone bodies | urine acetone bodies |
| min | Minute | minute |
| mPFS | Median progression-free survival | Median progression-free survival |
| MRI | Magnetic Resonance Imaging | nuclear magnetic resonance |
| MTD | Maximum tolerated dose | maximum tolerated drug dose |
| MedDRA | Medical Dictionary for Regulatory Activities | ICH International Medical Dictionary for Terminology |
| MOP | Medical Operation Procedure | Medical procedures |
| miRNA | MicroRNA | Microribonucleic acid |
| ^Na+^ | Plasma sodium | Blood sodium |
| NEUT | Neutrophil | neutrophile granulocyte |
| NCCN | National Comprehensive Cancer Network | The American National Comprehensive Cancer Network |
| NCI-CTC  NMPA | National cancer institute Common Terminology Criteria  National Medical Products Administration | National Institute of Oncology universal toxicity criteria  The National Medical Products Administration |
| OB | (Feces)Occult blood | (Bench) hidden blood |
| ORR | Objective response rate | Objective mitigation rate |
| OS | Overall survival | Overall survival |
| P | Phosphorus | phosphorus |
| PD | Progressive disease | PD |
| PD-1/PD-L1 | Programmed death 1/  programmed death ligand 1 | Programmed death receptor 1 / programmed death ligand 1 |
| PFS | Progression-Free-Survival | Progression-free survival |
| PPS | Per-Protocol Set | Meet the protocol set |
| PR | Partial response | partial remission |
| PT | Prothrombin time | prothrombin time |
| PLT | Blood platelet | blood cells |
| PI | Principal investigator | Principal investigator |
| PRO | Protein in urine | Urine protein |
| RBC | Red blood cell count | RBC |
| RECIST | Response evaluation criteria in solid tumors | Criteria for efficacy evaluation in solid tumors |
| RNA | Ribonucleic acid | ribonucleic acid |
| Sec | Second | second |
| SAE | Serious adverse event | Serious adverse events |
| SAP | Statistical analysis plan | Statistical analysis plan |
| SAS | - | SAS order |
| SD | Stable disease | stable disease |
| SOP | Standard Operation Procedure | standard practice |
| SS | Safety Analysis set | Safety analysis set |
| TBIL | Total bilirubin | total bilirubin |
| TCM | Traditional Chinese Medicine | Traditional Chinese medicine |
| TP | Plasma total protein | Total plasma protein |
| TKI | Tyrosine kinase inhibitor | Tyrosine kinase inhibitors |
| TSH | Thyroid-stimulating hormone | TSH |
| TT | Thrombin time | thrombin time |
| TTOR | Time to objective response | To ease the time |
| UA | Uric acid | purine trione |
| UBIL | Urine bilirubin | urine bilirubin |
| URBC | Urine red blood cell | Urinary red blood cells |
| ULN | Upper limit of normal | Upper limit of normal value |
| γ-GT | γ-glutamyltransferase | γ-glutamyltransferase |
| WBC | White blood cell count | leucocyte count |
|  |  |  |

# Introduction: Research background and scientific basis

## research background

Breast cancer is the highest incidence of female cancer in China and is on the rise, which is a common malignant tumor that endangers women's health. In China, according to the data collected by the National Cancer Register in 2017, the new cases of female breast cancer in 2014 were about 279,000, with the incidence of 41.82/1010000, ranking the first among female malignant tumors; the death cases were about 66,000, the mortality rate was 9.9 / 100,000, and the onset age is younger than that in Western countries. Breast cancer is the sum of a class of diseases, divided into at least four molecular subtypes: luminal A, luminal B, HER 2 overexpression and triple yin type. Among them, triple yin breast cancer (Triple negative breast cancer, TNBC) is a molecular type lacking estrogen receptor, progesterone receptor and human epidermal growth factor expression receptor 2, which accounts for about 15%~20% of the total breast cancer cases. It has the characteristics of [1] with high early recurrence rate, high distant metastasis rate and poor prognosis.

Because of the lack of effective targeted therapy for triple negative breast cancer, chemotherapy is still the conventional preoperative / adjuvant treatment for triple negative breast cancer. For early or locally advanced TNBC, according to the 2020 NCCN Breast Cancer Guidelines, the [3], the routinely recommended chemotherapy regimen is dose-intensive anthracycline combined with cyclophosphamide sequential paclitaxel (three weekly or weekly therapy), and paclitaxine, including doxoxorubicin, epirubicin, pyroubicin and polyPEGylated liposomal doxoxorubicin; taxetaxel and albumin-combined paclitaxel.

In the current neoadjuvant chemotherapy, the combination of anthracycline / taxanes achieves about 40% pCR in triple-negative breast cancer, while the addition of platinum agents can further improve this to approximately 50% [4-5]. However, the use of platinum drugs as neoadjuvant chemotherapy for TNBC remains controversial, although the addition of platinum improves pCR, the long-term outcome remains unknown, therefore, finding more effective treatments to improve the efficacy of early TNBC becomes a hot and difficult issue in research.

## scientific basis

### The use of albumin paclitaxel, immunotherapy, and apatinib in triple-negative breast cancer

#### Paclitaxel for injection (albumin-bound type) in triple-negative breast cancer

Taxanes are currently the treatment of choice for early breast cancer, including triple-negative breast cancer.

Paclitaxel for injection (albumin-bound type) (hereinafter referred to as "albumin paclitaxel") is a paclitaxel nanoparticle freeze-drying agent of human serum albumin as the carrier. A new dosage form of paclitaxel and human serum albumin is made into nanoparticles by nanotechnology. Albumin paclitaxel removes the co-solvent Cremo-phor EL of conventional paclitaxel injection, reduces the potential risk of solvent-related allergic reactions, and greatly reduces the drug infusion time to 30 minutes without pretreatment treatment. In addition, the human blood albumin as a drug carrier, using the characteristics of albumin binding-release, and using the cell membrane albumin receptor Gp 60 and cell membrane hole (caveolae), and tumor tissue rich in cysteine acid Asecretion protein (SPARC), promote drugs into tumor cells, achieve the purpose of targeted therapy, increase the anti-tumor curative effect.

Compared with paclitaxel has better pharmacokinetic properties, faster and higher tissue distribution, its distribution speed is 40 times the discharge rate, its binds to the albumin receptor in the tumor stroma, and the tumor tissue paclitaxel concentration is 26% higher than that of conventional paclitaxel. In 2005, based on a phase III study of albumin paclitaxel, albumin paclitaxel (Abraxane) was approved for advanced breast cancer, including 460 patients with advanced breast cancer, and the main efficacy index was response rate: 33% in albumin paclitaxel vs 19% for paclitaxel (p=0.001); time to disease progression between the two groups was 23.0 weeks vs16.9 weeks. In terms of safety: the proportion of grade 4 neutropenia was significantly lower in the albumin-paclitaxel group than in the paclitaxel group: 9% vs 22%. The incidence of grade 3 sensory nerve abnormalities was higher in the albumin-paclitaxel group (10% vs 2%), but was controllable and self-limiting with dose suspension or dose downregulation. Moreover, because the modified dosage form of albumin paclitaxel and the allergic reaction were significantly less than that of paclitaxel, the infusion time was also significantly shortened in the clinical application process. A total of 186 patients (41% of all subjects) were included in patients with advanced disease without paclitaxel or prior adjuvant therapy but with longer than 1 year of relapse. Among the first-line patients, the objective response rate was significantly higher in the albumin-paclitaxel group compared with the paclitaxel group (42% vs 27%, p=0.029). Time to no disease progression (PFS) also showed a longer trend, although no statistical difference was shown (24 weeks vs 19.7 weeks, p=0.173).

In January 2005, the US Food and Drug Administration (FDA) approved the albumin-paclitaxel Abraxane to market. In September 2018, the National Medical Products Administration (NMPA) approved the marketing of albumin paclitaxel (Aiyue), which is suitable for treating metastatic breast cancer that failed combination chemotherapy or breast cancer that relapsed within 6 months after adjuvant chemotherapy. The NCCN guidelines, ESMO guidelines and China breast cancer guidelines, both recommend albumin paclitaxel as a single agent for recurrent or metastatic breast cancer. Among them, the NCCN guidelines recommend that neoadjuvant treatment for triple-negative breast cancer should use paclitaxel instead of paclitaxel or docetaxel, measuring no more than 125mg / m2 per week.

#### Application of anti-PD-1 antibodies in triple-negative breast cancer

In recent years, with the deepening of the understanding of the immune system and the rapid development of biotechnology, tumor immunotherapy has made a breakthrough progress. Immunotherapy has become an important means of tumor treatment, and occupies an increasingly important position in the comprehensive treatment system of cancer.

The PD-1 full name is the programmed death molecule 1 (Programmed death-1), which is a negative costimulatory molecule found in recent years. PD-L1 and PD-L2 are ligands for PD-1 that can bind specifically to PD-1. By highly expressing PD-L1 molecules, tumor cells bind to PD-1 molecules on T lymphocytes to transmit negative regulatory signals, leading to the induction of apoptosis and immune inability of tumor antigen-specific T cells, and enabling tumor cells to evade the immune monitoring and killing of the body. PD-1 inhibitors are a new class of tumor immunotherapy drugs, which regulate the anti-tumor activity of T lymphocytes and cause tumor apoptosis by blocking the PD-1 / PD-L1 signaling pathway.

Since 2014, the United States food and drug administration (FDA) based on breakthrough efficacy, gradually approved anti-PD-1 monoclonal antibody (Squibb company Nivolumab and Pembrolizumab) for the treatment of advanced melanoma, non-small cell lung cancer, renal cell cancer, head and neck cancer, Hodgkin's lymphoma, sorafenib treatment of liver cell carcinoma and PD-L1 positive cervical cancer patients, including advanced, standard treatment failure, no effective treatment of patients, also advance to a second line treatment. In addition, anti-PD-1 monoclonal antibodies have conducted a series of hundreds of clinical trials, including advanced solid tumors and malignant blood diseases, because of their lasting efficacy and relatively mild adverse effects. The results of previous trials showed higher response rate and long-term survival rate compared with existing therapies.

More than 50 years ago, scientists found that the degree of TILs infiltration was negatively correlated with breast cancer recurrence. With further research, the degree of TNBC chemotherapy benefit was correlated with the density of TILs.

Although breakthroughs have been made in tumor immunotherapy in recent years. However, many triple-Yin breast cancer patients did not achieve good efficacy with anti-PD-1 / PD-L1 antibody alone. Pembrolizumab (KEYNOTE 012 / 086 / 119) and Atezolizumab (NCT01633970) have conducted more single-agent clinical studies [6] in triple-negative breast cancer.

KEYNOTE-012 (NCT01848834) is a multicenter, open phase Ib clinical study of advanced TNBC, gastric cancer (PD-L1 positive tumor stroma or 1% tumor cells, uroepithelial cancer and head and neck cancer) to evaluate the safety and efficacy of intravenous Pembrolizumab mAb in patients with PD-L1 positive solid tumors. Results of safety and efficacy in TNBC: Detection of PD-L1 expression in 111 advanced TNBC tumor specimens showed that 65 (58.6%) tumor specimens were positive for PD-L1. Thirty-two subjects were enrolled and evaluated for safety and tumor efficacy.27 assessed antitumor efficacy with an ORR of 18.5%, 1 CR (3.7%), 4 PR (14.8%), and 7 SD (25.9%) with median response time of 17.9 weeks (7.3-32.4 weeks) and median duration of response not reached (15-47.3 weeks). Common trial drug-related adverse events (TRAEs) included mild arthralgia (18.8%), fatigue (18.8%), myalgia (18.8%), and nausea (15.6%), and 5 subjects had a grade 3 TRAE and one had a drug-related death.

KEYNOTE-086 CohortB (NCT02447003) was enrolled in PD-L1 positive advanced TNBC to assess the safety and efficacy of Pembrolizumab mAb as its first-line therapy. Study Results: 137 patients were screened, and 79 patients (58%) were tested positive for PD-L1. A total of 52 subjects were enrolled with an ORR of 23%, CR 4%, PR 19%, and SD 17%; the median response time was 8.7 weeks (8.1-17.7 weeks). The median PFS was 2.1 months (95% CI,2.0-3.9). In 37 cases (71%), fatigue (31%), nausea (15%), and diarrhea (13%) were common.

Single agent is about ORR 20% in PD-L1 positive advanced TNBC patients, so it is urgent to find drugs that can be combined with anti-PD-1 / PD-L1 antibodies and enhance their efficacy. A large number of ongoing phase I / II clinical trials are combination targeted therapies and immunotherapy. The basic support for these combination therapies is that the two therapies combine different immunology and tumor biological mechanisms to enhance anti-tumor activity; in addition, some experiments show that targeted therapies can enhance certain aspects of cancer-immune circulation (such as tumor antigenicity, T cell initiation / transport / infiltration, etc.).

#### Application of antiangiogenic agents in triple-negative breast cancer

In 1971, Judah Folkman first proposed the vascular dependence theory of tumor growth, indicating that angiogenesis plays an important role in the development of tumors. Current studies show that tumor angiogenesis is a landmark process in the development of solid tumors, and is critical for rapid tumor growth, invasion and metastasis. And antiangiogenic therapy prevents tumor development, spread, and metastasis by inhibiting tumor vascular generation. Among them, VE GF is a key driver of angiogenesis, which participates in the generation of tumor neoformed blood vessels by mediating various mechanisms such as vascular endothelial cell proliferation, migration, infiltration, changing vascular permeability and vascular dilation. VE GF A is the most important vascular endothelial growth factor (VE GF), which induced the strongest tumor blood vessel formation. VE GF-A protein expression and gene amplification was higher in TNBC. This suggests that anti-angiogenic agents may be active in TNBC.

**Application of apatinib monotherapy in triple-negative breast cancer**

Apatinib (apatinib) is a new targeted therapeutic drug independently developed in China. It is a small molecule tyrosine kinase inhibitor (Tyrosine kinase inhibitor, TKI), which can highly selectively compete for the ATP binding site of VE GF R-2 in cells, block downstream signal transduction, and inhibit neoangiogenesis in tumor tissue. The antitumor effect of apatinib monotherapy has been confirmed in several clinical studies related to solid tumors, and it was approved by the National Food and Drug Administration (China Food and Drug Administration, CFDA) in 2014. Its marketing registry study is a prospective, randomized, double-blind, placebo-controlled, multicenter phase clinical study evaluating the efficacy of apatinib monotherapy in patients with advanced / metastatic gastric cancer who have failed second-line chemotherapy. The results showed that apatinib significantly prolonged OS in patients with advanced / metastatic gastric cancer, with a median OS of 7.6 months, 2.6 months longer than placebo (P=0.0027), and the risk of death decreased by nearly 40%. In addition, the median PFS in the apatinib treatment group was 2.8 months, 0.9 months longer than the placebo group (P &lt;0.0001), or a nearly 55% reduction.

Apatinib in a phase clinical study of triple-negative breast cancer [7], mainly enrolled patients with triple-negative breast cancer who had failed at least a third-line chemotherapy regimen (including anthracyclines and taxane). Use apatinib 750 mg qd orally or 500 mg qd orally. In the Phase A study with a starting dose of 750 mg qd, 84% of subjects had dose reduction (21 patients). Nine subjects (36%) had DLTs, including 1 grade 4 thrombocytopenia, 3 grade 3 plus hand and foot syndrome, 3 uncontrolled hypertension, 1 symptomatic pericardial effusion and 1 uncontrolled atrial fibrillation. Three subjects had SAEs, two of which were considered as drug-related deaths (1 bronchoalveolar hemorrhage and one sudden death of unknown cause). Considering the above safety concerns, 500 mg qd was chosen as the starting dose. In the phase b study, most AEs of 500 mg qd oral apatinib were mild to moderate (grade 1-2), and the most common AEs were hypertension, proteinuria, and hand and foot syndrome. Two affected subjects experienced grade 4 thrombocytopenia (3.4%). The dose suspension and / or dose reductions were 15 (25.4%) and 19 (32.2%), respectively. There were 3 subjects with drug-related SAEs, 2 of whom died within 28 days of the last treatment.

In terms of efficacy: the results of phase A (single center, oral apatinib 750 mg qd) showed 22 evaluable patients had a median PFS of 4.6 months, ORR 36.4%. Phase b (multicenter, oral apatinib 500 mg qd) showed a median PFS of 3.3 months, ORR 10.7%, CBR 25%.

Through this study, it is seen that apatinib monotherapy has some efficacy against metastatic triple yin breast cancer at tolerable doses, but the efficacy is limited.

### Scientific basis for combination drug use development

#### Overview of Camrelizumab (development code: SHR1210)

This clinical study involves recombinant humanized anti-PD-1 monoclonal antibody injection (Camrelizumab), a new drug for therapeutic biological products developed by Jiangsu Hengrui Pharmaceutical Co., Ltd., which has been marketed in China. It can specifically block the binding of PD-1 to PD-L1 and terminate the PD-1 immunosuppressive signal caused by the interaction between PD-1 and PD-L1 in T cells. Preclinical research data show that compared with similar foreign drugs, it has considerable body efficacy and safety. As of August 25,2018,21 clinical studies of Camrelizumab have been conducted.

The highest incidence of AEs (TEAEs) during treatment was reactive cutaneous capillary hyperplasia (in> 30% of subjects receiving Camrelizumab ). Other common reported TEAEs (occurring in> 10% of Camrelizumab subjects) include anemia, fatigue, fever, elevated transaminase (elevated alanine aminotransferase [ALT] and / or increased aspartate aminotransferase [AST]).

The serious adverse events (SAEs) most frequently reported (approximately 1% of subjects) in all studies, included reactive cutaneous capillary dysplasia, pulmonary infection, pneumonia, upper gastrointestinal (GI) bleeding, liver failure, and malignant progressive tumors. Immune-related SAE pneumonia (in 1.1% of subjects) and interstitial lung disease (in 0.5% of subjects) were considered Camrelizumab-related adverse events.

#### Justification for the combination of Camrelizumab with chemotherapy

In addition to causing tumor cell death through cytotoxicity, more and more studies have proved that chemotherapy drugs have a role in enhancing the anti-tumor immune function of the body. Clinical practice suggests that T cells and NK cells are more functional than in untreated breast cancer patients (stage II / III). Chemotherapy kills tumor cells to release a large number of tumor antigens, increase the amount of cross-presented tumor antigens, and then stimulate the body to produce an immune response. The combined blockade of T cell activation caused by PD-1 / PD-L1 inhibitors, identifying the signal of releasing tumor antigens by standard chemotherapy, further enhancing the immune response, and killing tumor cells, benefits patients. This suggests that PD-1 / PD-L1 mAb combined with standard chemotherapy is a feasible therapeutic strategy.

Anti-PD-1 antibodies have limited single-agent efficacy in PD-L1 positive triple-negative breast cancers, and based on the presence of TILs and the association of PD-L1 over-expression in breast cancer, combination chemotherapy and immunotherapy became the current hot anticancer strategy in triple-negative breast cancer. Currently, Pembrolizumab (PD-1 mAb) and Atezolizumab (PD-L1 mAb) have been combined with chemotherapy in TNBC. In TNBC, albumin paclitaxel can be used as the best co-chemotherapeutic drug because of its efficacy, safety, clinical operability, and the avoidance of immunosuppressive effects caused by steroids. The phase Ib results showed that Atezolizumab combined with albumin paclitaxel in triple negative breast cancer without selected PD-L1 status, 32 evaluable cases ORR 38% and 6 months DCR 52%. Based on this result, the continued phase clinical study of Atezolizumab combination chemotherapy (NCT02425891, IMpassion130) has made a breakthrough.

IMpassion130 Is a multicenter, randomized, double-blind study [8], enrolled advanced TNBC patients without previous systemic anti-tumor therapy in the metastatic stage, to evaluate the efficacy, safety and pharmacokinetics of Atezolizumab plus albumin paclitaxel versus placebo combined with albumin paclitaxel in the first-line treatment of patients with advanced TNBC.

Patients in the study were randomized in a 1:1 ratio to the Atezolizumab + albumin paclitaxel treatment group or the placebo + albumin paclitaxel control group. The stratification factors were receipt of taxane treatment in the neoadjuvant or adjuvant phase, presence of liver metastasis at baseline, and expression of baseline PD-L1 (positive VS negative). The two primary endpoints of the study are PFS (as assessed by RECIST 1.1) and overall survival (OS).

Results: A total of 902 patients were enrolled, including 451 patients in the treatment and control groups. Results of the intent-to-treat population (ITT) analysis showed that the median PFS for treatment group was 7.2 months, the median PFS was 5.5 months (hazard ratio, 0.80; 95% CI,0.69-0.92; P = 0.002), the median OS was 21.3 months, and the median OS was 17.6 months for control group (HR 0.84; 95% CI, 0.69-1.02; P = 0.08). Analysis of the PD-L1 positive subgroup data showed that median PFS in treatment group was 7.5 months and median PFS was 5.0 months (HR 0.62; 95% CI, 0.49-0.78; P <0.001); median OS in treatment group was 25 months, and median OS was 15.5 months in control group (hazard ratio, 0.62; 95% CI, 0.45-0.86).

Based on this finding, on March 8,2019, the FDA preferentially passed the review for triple-negative breast cancer combined with first-line chemotherapy with atezolizumab for unresectable locally advanced or metastatic PD-L1 positive treatment. It also provides a strong basis for the combination of PD-1 / PD-L1 antibody drugs and chemotherapy in the treatment of triple-negative breast cancer.

KEYNOTE-522 [9] is a Phase III randomized double-blind trial to explore the efficacy of plus chemotherapy versus placebo as neoadjuvant therapy in patients with TNBC, and pembrolizumab versus placebo as adjuvant therapy in patients with TNBC. Treatment-naive T1c N1-2 or T2-4 N0-2 TNBC 2:1 randomized to receive neoadjuvant pembrolizumab (200mg Q3W) or placebo were treated with 4 cycles of paclitaxel + carboplatin and 4 cycles of doxorubicin or epirubicin + cyclophosphamide (neoadjuvant). Following surgery, patients received pembrolizumab or placebo adjuvant therapy for 9 cycles until relapse or unacceptable toxicity. Stratification factors included lymph node status (positive or negative), tumor size (T1/T2 or T3/T4), and carboplatin regimen (Q3W or QW). The primary endpoints were the pCR and EFS as defined by ypT 0 / Tis ypN0. Secondary endpoints included efficacy in the pCR, overall survival (OS), and PD-L1 + population as defined by ypT0 ypN0 and ypT 0 / Tis. At a median follow-up time of 15.5 months, patients were randomly assigned to receive pembrolizumab (n=784) or placebo (n=390). In 602 evaluable patients, pembrolizumab + chemotherapy significantly improved pCR rates (ypT 0 / Tis ypN0) versus placebo + chemotherapy, with pCR rates of 64.8% (95%CI, 59.9-69.5) and 51.2% (95%CI, 44.1 to 58.3) (P=0.00055). In the PD-L1 positive and negative populations, the pCR rate (ypT 0 / Tis ypN0) was 68.9%vs 54.9% vs 4.9% and 45.3%vs 30.3%, respectively. The addition of pembrolizumab to neoadjuvant chemotherapy and sequential adjuvant pembrolizumab therapy prolonged EFS in the patient (HR 0.63 [95%CI,0.43-0.93]). In terms of safety, the incidence of treatment-related adverse events was 78.0% and grade 3 or higher for pembrolizumab and placebo plus chemotherapy and 73.0%, respectively.

IMpassion031[10] Study a multicenter, randomized, double-blind phase clinical study designed to evaluate the efficacy and safety of Atezolizumab combined with chemotherapy in the neoadjuvant therapy of TNBC. A total of 333 untreated TNBC patients aged 18 years, tumors&gt; 2cm and pathologically confirmed invasive stage II-III, were randomized 1:1. The experimental group received 12 weeks of Atezolizumab (840mg, IV, Combined q2w) with Nab-P (125mg / m2, IV, qw), Sequential 8 weeks of Atezolizumab (840mg, IV, Combined q2w) doxorubicin (60mg / m2, IV, And q2w) + cyclophosphamide (600mg / m2, IV, q2w); The control group received 12 weeks of placebo in combination with Nab-P (125mg / m2, IV, qw), Sequential 8-week placebo combined with doxorubicin (60mg / m2, IV, And q2w) + cyclophosphamide (600mg / m2, IV, q2w). All patients were underwent surgery and pathological complete response was evaluated, stratified by clinical stage of breast cancer and tumor-infiltrating immune cell PD-L1 expression (IC 1% VS &lt;1%). The primary study endpoint was pCR rate in patients with ITT or PD-L1 positive (IC 1%), and the secondary study endpoint included overall survival, event-free survival, disease-free survival and quality of life indicators. A preset unilateral P &lt;0.0184 was statistically significant.

The study showed that the pCR rate increased 16.5% (57.6% vs. 41.1%, P=0.0044), and the pCR rate increased 19.5% (68.8% vs. 49.3%, P=0.021). In terms of safety, the incidence of grade 3-4 adverse reactions in the Atezolizumab and placebo groups was 56.7% and 53.3%, respectively, during neoadjuvant therapy. Atezolizumab Significantly increased pCR rate in TNBC patients independent of PD-L1 expression and with good safety.

Based on the results of the KEYNOTE-522 and IMpassion031 studies, we further demonstrated the efficacy and safety of PD-1 / PD-L1 antibody drug combination chemotherapy in triple-negative breast cancer. It also provides a strong basis for the combination of Camrelizumab and chemotherapy for triple negative breast cancer.

#### Basis for the combination of anti-PD-1 antibodies and antiangiogenic agents

The VE GF and VE GF R pathways regulate the immune response by increasing DNA damage and tumor mutation load. The VE GF R signaling pathway plays an important role in mediating tumor immune escape, and inhibition of this pathway may enhance the activating tumor immune effects of PD-1 antibodies.

Preclinical studies have shown that in the tumor microenvironment, antiangiogenic drugs can not only inhibit tumor vascularization, but also sensitize anti-PD-1 / PD-L1 treatment to [11-12] by increasing PD-L1 expression and CD8 + T cell infiltration. Also found in animal studies that low doses of antiangiogenic agents sensitize breast cancer to PD-1 by increasing tumor infiltrating CD8 + T and B cells and elevated CD45 + immune cells in the tumor microenvironment, and antiangiogenic therapy may enhance response to PD-1 / PD-L1 arrest and improve survival [13].

At present, a number of clinical studies of PD-1 antibody carilzumab combined with apatinib, involving hepatocellular carcinoma, gastric cancer, non-small cell lung cancer and breast cancer. The preliminary results confirm that Camrelizumab and apatinib are safe and tolerable, and the combination therapy plays a certain role in enhancing the efficacy.

In a phase I clinical study of Camrelizumab and apatinib for advanced liver, gastric, and esophago-gastric junction cancer, Phase 1b is a dose-climbing study, Apatinib 125 mg/d, 250 mg/d, 500 mg/d was administered in combination with Camrelizumab (200mg, Dose exploration of q2w), DLT not reported in the apatinib 125 mg cohort, One patient in the apatinib 250 mg cohort had grade 3 DLT, Three patients in the 500-mg cohort developed grade 3 immune-related pneumonia, Thus, in the phase Ib extension study, The recommended dose of apatinib is 250 mg/d, And this dose group was well tolerated by [14].

In another phase II study of PD-1 antibody SHR-1210 with apatinib or FOLFOX4 for liver cancer, led by the 81st Hospital of PLA, the dose exploration of apatinib 125 mg/d, 250 mg/d, 375 mg/d, and 500 mg/d with SHR-1210 was conducted, showing that no DLT in apatinib 125,250 and 375mg groups, while two patients in the apatinib 500mg group had DLT with grade 3 diarrhea. Therefore, the dose of Camrelizumab combined with apatinib 375 mg was selected in the dose extension phase, with the interruption of apatinib treatment in all 19 patients enrolled in the dose extension phase, and 8 patients (42.1%) reduced the dose to 250 mg within 2 months of initiation of treatment. In addition, basic studies have found that high-dose anti-VE GF treatment may aggravate the hypoxia and immunosuppression of the tumor microenvironment. Therefore, considering the safety and efficacy, this study recommends a lower dose of apatinib (250 mg, QD) [15] in further combination therapy studies.

Apatinib combined with Camrelizumab in a phase clinical study of advanced triple-negative breast cancer [16], 40 advanced TNBC patients 1:1 randomized to Camrelizumab (200mg Q2W) + apatinib (250mg qd d1-d14), Camrelizumab (200mg Q2W) + apatinib (250mg qd d1-d7), the primary endpoint was ORR. Since the 10 subjects randomized to intermittent apatinib 1:1, all subjects in the second phase were enrolled in the continuous apatinib group. In the continuous apatinib administration group, the ORR was 43.3% (95%CI 25.5% -62.6%), no objective response was found in the first phase (ORR 0%, 95%CI 0% -30.8%), and this cohort was not further recruited after the first phase. In the consecutive apatinib arm, the ORR was 50.0% for patients receiving 95%CI 18.7% as a first-line treatment) and the ORR was 40.0% (95%CI 19.1% -63.9%). In the continuous dose group, the median PFS was 3.7 months (95%CI 2.0 to 6.4) compared to 1.9 months (95%CI 1.8 to 3.7) in the intermittent dose group. In terms of safety, the most common adverse events were fatigue (57.5%), hand-foot syndrome (55.0%), AST elevation (80.0%), and ALT (62.5%) with no treatment-related deaths. The main adverse events are detailed in Table 1.

Table 1 Major adverse events in patients in the intermittent and continuous administration groups of apatinib

| AE | Serial group (n=30) | Intermittent administration group (n=10) |
| --- | --- | --- |
| feel like vomiting | 4（13.3%） | 2（20.0%） |
| vomit | 4（13.3%） | 2（20.0%） |
| tired | 14（46.7%） | 9（90.0%） |
| hand-foot syndrome | 17（56.7%） | 5（50.0%） |
| AST go up | 25（83.3%） | 7（70.0%） |
| ALT go up | 18（60.0%） | 7（70.0%） |
| diarrhoea | 6（20.0%） | 1（10.0%） |
| hypertension | 11（36.7%） | 3（30.0%） |
| hematochezia | 0（0.0%） | 1（10.0%） |
| anemia | 5（16.7%） | 2（20.0%） |
| leuco penia | 11（36.7%） | 3（30.0%） |
| albuminuria | 16（53.3%） | 5（50.0%） |
| RCCEEP | 3（10.0%） | 3（30.0%） |
| erythra | 9（30.0%） | 1（10.0%） |
| gingival dleeding | 1（3.0%） | 2（20.0%） |
| hypothyrea | 7（23.3%） | 1（10.0%） |
| hyperthyrea | 4（13.3%） | 0（0.0%） |
| Any Grade 3 or 4 adverse event | 7（23.3%） | 2（20.0%） |

For the first-line treatment of advanced triple-negative breast cancer, a randomized controlled phase III clinical study of Camrelizumab (200mg, q2w) plus apatinib (250 mg, qd) plus albumin paclitaxel is ongoing.

#### Justification for the combination of antiangiogenic agents and chemotherapy

Bevacizumab (Bevacizumab) is a recombinant human anti-VE GF monoclonal antibody. Bevacizumab has been approved as a combination chemotherapy for first-line treatment for metastatic breast cancer in many countries (> 80 countries, except the US). Several clinical studies have demonstrated that bevacizumab combined with chemotherapy can significantly increase the neoadjuvant pCR rate of HER 2-negative breast cancer. NSABP-B40 study [17] showed a significantly higher pCR rate in bevacizumab compared with chemotherapy alone (34.5% vs. 28.02%, P=0.02); GeparQuinto Study [18] also showed a significantly higher pCR rate in EC-T + bevacizumab in HER 2-negative breast cancer compared with EC-T (18.4% vs. 14.9%, p = 0.042).

The above findings suggest that antivascular targeting drugs can improve the efficacy of single-agent chemotherapy in metastatic triple-negative breast cancer.

#### The combination of Camrelizumab, apatinib and chemotherapy

Immunocombination therapy is the most popular research direction at present, and many studies have confirmed that anti-PD-1 / PD-L1 antibody and other immunomodulatory agents, chemotherapy, molecular targeting combination can significantly improve the efficacy of immunotherapy.

Based on the results of a phase III study of pabolizumab [19] and atilizumab [8], Anti-PD-1 antibody pabolizumab plus chemotherapy and anti-PD-L1 antibody atenizumab plus albumin paclitaxel have been approved in the United States for the first-line treatment of PD-L1 positive metastatic triple negative breast cancer, Also targeting the neoadjuvant treatment for early triple-negative breast cancer, Large randomized controlled phase III studies of pembrolizumab [9] and atezolizumab [10] have both demonstrated significant efficacy, And the safety is controllable, Based on the results of KEYNOTE-522 studies with pabolizumab in early TNBC, The FDA has approved pembrolizumab as neoadjuvant therapy in patients with high-risk early triple-negative breast cancer, Postsurgery continued as adjuvant single agent. Therefore, it provides a basis for the development of anti-PD-1 antibody Camrelizumab combination therapy in the early TNBC. In addition, the SHR-1210-III-303-NSCLC study (NCT03134872) is a phase III, randomized, open-label controlled study of Camrelizumab combination therapy evaluating the efficacy and safety of Camrelizumab combined with pemetrexed and carboplatin in first-line treatment in patients with advanced or metastatic non-squamous non-small cell lung cancer. The results showed that PFS of Camrelizumab with pemetrexed and carboplatin was significantly better than pemetrexed and carboplatin (11.27 months vs. 8.25 months, HR 0.599, p= 0.0001) with manageable safety. The main adverse reactions were reactive capillary hyperplasia, anemia, neutropenia, leukopenia, increased AST, elevated ALT, thrombocytopenia, nausea, fatigue, decreased appetite, constipation, vomiting, increased GGT, edema, increased blood bilirubin, rash, pruritus, hypothyroidism, hypoalbuminemia, lymphopenia, and elevated blood creatinine. The most common grade 3 adverse effects were neutropenia, anemia, and leukopenia. Based on the results of this study, it has been approved for the first-line treatment of advanced or metastatic non-squamous non-small cell lung cancer in China.

Consider the mechanism of synergy of anti-vascular targeting drugs with combined immunization and combination chemotherapy. At present, the combination of bevacizumab combined with PD-L1 antibody atezolizumab has been carried out for several tumor species. The IMpower150[20] study, published in the New England Journal of Medicine in June 2018, was a randomized, controlled phase study of first-line treatment in patients with metastatic non-small cell lung cancer (NSCLC), comparing the efficacy and safety of atezolizumab plus chemotherapy (carboplatin plus paclitaxel) ± bevacizumab vs bevacizumab combined with chemotherapy. The results showed that PFS in Atezolizumab combined with bevacizumab + carboplatin + paclitaxel (ABCP group) was significantly better than bevacizumab + carboplatin + paclitaxel (BCP group) (median PFS: 8.3 months vs. 6.8 months; HR=0.62,95%CI=0.52-0.74; P <0.001) and was well tolerated, similar to previously reported safety data. Adverse events related to any treatment were 94.4% in the ABCP group and 95.4% in the BCP group, and the incidence of grade 1 or 2 treatment-related adverse events was 35.9% in the ABCP group and 45.4% in the BCP group. The most common grade 3 / 4 treatment-related adverse events were neutropenia, decreased neutrotrophil count, febrile neutropenia, and hypertension. The overall incidence of immune-related adverse events occurring in the ABCP group was 77.4%, and the most common immune-related adverse events were skin rash, hepatitis, hypothyroidism, hyperthyroidism, pneumonia, and colitis.

FUTURE-C-PLUS study [21] is a prospective, single arm, phase II study of Camrelizumab combined with antiangiogenic drug famitinib + albumin paclitaxel first-line treatment of immune regulatory advanced triple negative breast cancer, which mainly explores the efficacy and safety of Camrelizumab combined with antiangiogenic drug + chemotherapy in immune regulatory mTNBC. The results showed that Camrelizumab and the antiangiogenic drug famitinib + albumin paclitaxel had excellent first-line treatment of immunomodulatory mTNBC. The ORR in the ITT population was 81.3%, which was the highest ORR in the first-line treatment of mTNBC. Although PFS was not mature, the PFS rate at 9 months was 60.2%, and the 10-month PFS rate was 53.4%. In terms of safety, the combination arm had no unexpected adverse events and was relatively well tolerated, essentially consistent with previous reports of anti-PD-1 / PD-L1 antibodies and anti-VE GF inhibitors or chemotherapy. Major adverse events included neutropenia, anemia, thrombocytopenia, fatigue, anorexia, elevated TSH, nausea, vomiting, peripheral sensory neuropathy, hypertension, hypothyroidism, ALT / AST elevation, palmoplantar redness syndrome; the most common grade 3 / 4 adverse events included neutropenia, anemia, thrombocytopenia, and febrile neutropenia.

Based on the experience of the above studies, it provides a strong basis for combining Camrelizumab with the antiangiogenic drug apatinib and neoadjuvant chemotherapy for early TNBC.

## Potential risks and benefits

### Known potential risks

Any research drug or therapy may have unforeseen or even serious toxic side effects.

Albumin paclitaxel, epirubicin, cyclophosphamide are common clinical chemotherapy drugs, adverse reaction spectrum has been very clear, common adverse reactions include: bone marrow suppression including leukopenia, neutropaenia, thrombocytopenia, anemia, peripheral neuropathy, muscle and joint pain; allergic reaction, nausea, vomiting, mucositis, fatigue; motor neurotoxicity, sensory neurotoxicity, cardiovascular events, alopecia, local reaction at the injection site, diarrhea, etc.

Common adverse events of apatinib in previous clinical trials were hypertension, proteinuria, palmoplantar redness syndrome, bleeding, myelosuppression, hyperbilirubinemia, fatigue, ALT / AST, anemia, vomiting, decreased appetite, and occult blood positive, but most adverse events were mild to moderate (I / II).

In the phase II study of Camrelizumab plus apatinib for advanced triple-negative breast cancer, adverse events included fatigue, hand-foot syndrome, elevated AST, ALT, elevation, nausea, vomiting, hypertension, proteinuria, suppression, rash, hypothyroidism or hyperthyroid, reactive capillary hyperplasia.

In the Phase II study of Camrelizumab combined with the antiangiogenic agent famitinib plus ALB for immune regulatory advanced triple-negative breast cancer (FUTURE-C-PLUS), major adverse events included neutropenia, anemia, thrombocytopenia, fatigue, anorexia, elevated TSH, nausea, vomiting, peripheral sensory neuropathy, hypertension, hypothyroidism, elevated ALT / AST, and palmoplantar redness syndrome; the most common grade 3 / 4 adverse events included neutropenia, anemia, thrombocytopenia, and febrile neutropenia.

According to the public data published in clinical studies of similar products, when anti-PD-1 / PD-L1 antibody is combined with apatinib + chemotherapy, toxicity may be superimposed, and the incidence and severity of adverse effects may be increased. Possible adverse reactions include hematologic adverse reactions such as decreased neutrophil count, white blood cell count, decreased platelet count and anemia; elevated alanine aminotransferase, elevated aspartate aminotransferase, increased blood glucose, hypoesthesia, rash, pruritus, fever, fatigue, nausea, diarrhea, vomiting and other non-hematologic adverse reactions; And immune-related adverse reactions, with a higher incidence of immune-related interstitial pneumonia, hypothyroidism or hyperthyroidism, and a lower incidence (1%) of immune-related colorectal proctitis, nephritis, hepatitis, hypophysitis, adrenal insufficiency, etc. Most of these adverse reactions are mild and can be controlled, and a few are serious adverse events. The vast majority of adverse events can be adequately controlled under established toxicity procedures.

In addition, for all the large molecular protein monoclonal antibody drugs, other risks include infusion reaction: mainly for chills, chills, facial and limbs osis, then fever, accompanied by nausea, vomiting, headache, dizziness, irritability, fall, severe coma, blood pressure, shock and respiratory failure symptoms, is intravenous infusion by heat source, drugs, impurities, liquid temperature is too low, liquid concentration is too high and many factors such as infusion too fast. As a fully humanized monoclonal antibody, Camrelizumab is expected to have a lower incidence and a lesser degree.

### Probably known to benefit

As mentioned above, triple-negative breast cancer is refractory breast cancer with a poor prognosis, and immunotherapy combined with antiangiogenic drugs and chemotherapy is a new strategy expected to further improve the ability of the immune system to kill tumors. PD-1 antibody combined with apatinib plus chemotherapy provides a better treatment option for neoadjuvant therapy in patients with triple-negative breast cancer.

Foreign large phase III studies have shown that the neoadjuvant therapy of immunocombination chemotherapy for early triple-negative breast cancer is significant. At present, the neoadjuvant therapy of triple-negative breast cancer recommended in China is still based on taxane and anthracycline chemotherapy drugs, etc. The subjects have received standard chemotherapy, and their clinical benefit and tolerance can be expected.

## Research innovation

Triple-negative breast cancer, as a poor prognosis of refractory breast cancer, has still taken chemotherapy as the main treatment for many years. At present, preclinical studies have shown that immune combined with anti-angiogenic drugs can significantly improve anti-tumor activity, and clinical studies have also shown that Camrelizumab combined with anti-angiogenic drugs plus chemotherapy has good efficacy in the treatment of advanced TNBC with controllable safety. However, for the neoadjuvant treatment of early breast cancer, there is no research data of similar treatment at home and abroad.

Therefore, on the basis of previous studies, clinical studies of PD-1 immunotherapy combined with apatinib and neoadjuvant chemotherapy for triple-negative breast cancer are considered, aiming to explore more optimal treatment options for such patients.

# Study objective and study endpoint

## purpose of research

### Main research objectives

- To evaluate the efficacy of Camrelizumab combined with apatinib plus chemotherapy in neoadjuvant treatment of triple negative breast cancer.

### Secondary research purpose

- To evaluate the efficacy and safety of Camrelizumab combined with apatinib plus chemotherapy in neoadjuvant treatment of triple-negative breast cancer.

### Exploratory study objectives

- To explore potential predictive biomarkers related to efficacy in peripheral blood and tumor specimens.

## Study end point

### Main study endpoints

- To evaluate the pathological complete response rate (tpCR) (ypT 0 / is ypN0) of Camrelizumab plus apatinib plus chemotherapy for neoadjuvant treatment of triple-negative breast cancer.

### Secondary study endpoints

- To evaluate the efficacy of Camrelizumab combined with apatinib + chemotherapy in neoadjuvant treatment of triple negative breast cancer: bpCR (ypT 0 / is), RCB-0 / I, ORR, EFS, iDFS;
- To evaluate the safety of Camrelizumab plus apatinib + chemotherapy in neoadjuvant treatment in triple-negative breast cancer: adverse events (AEs), serious adverse events (SAEs), abnormal laboratory tests, etc.

### Exploratory study endpoints

- Potential predictive biomarker parameters related to efficacy (e. g., pCR, RCB-0 / I, EFS, iDFS, etc.) in peripheral blood and tumor tissue specimens, including but not limited to PD-L1, CTCs, TILs, CD4 / CD8, Tregs, BRCA 1 / 2, and PI3K / AKT / mTOR.

# research design

This study is a single arm, prospective phase II clinical study scheduled to enroll 35 screened eligible subjects receiving 8 cycles of Camrelizumab plus apatinib plus chemotherapy neoadjuvant therapy.

The overall design of the study is as follows:


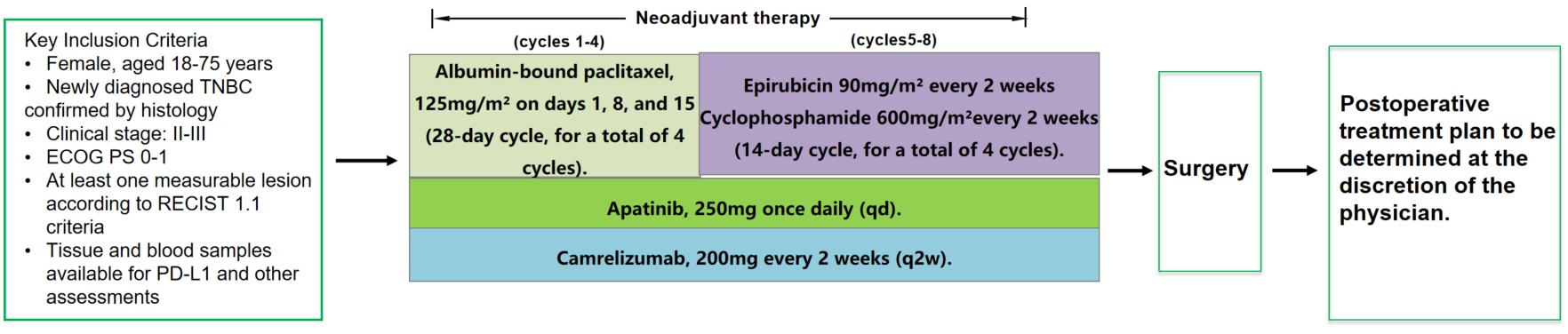


In this study, the screening period shall not exceed 28 days, and the subjects who pass after completing the screening examination and assessment shall enter the study treatment period. Subject will be administered for 72h after enrollment, 1-4 cycles every 4 weeks, 5-8 cycles every 2 weeks until the end of the course or disease progression, intolerable toxicity, subject withdrawal of consent, or the investigator.

# Subject selection and withdrawal

## Enrollment criteria

Subjects must meet all of the following inclusion criteria to be enrolled in this trial:

1. Women aged 18 years and 75 years old, treated for primary breast cancer;
2. Histopathologically confirmed early or locally advanced triple-negative invasive breast cancer, as defined by the latest ASCO / CAP guidelines, along with the following conditions:

- Pathological types were triple negative, specifically: ER negative: IHC <1%, PR negative: IHC <1%, HER 2 negative: IHC 0 / 1 + or IHC2 + but ISH negative;
- Tumor stage: stage II-III [according to the American Joint Committee on Cancer (AJCC) criteria].

1. ECOG score of 0~1;
2. The expected survival period is not less than 3 months;
3. At least one measurable lesion was present as per the RECIST 1.1 criteria;
4. The functional level of the organs must meet the following requirements:
   1. routine blood test
      - Neutrophil count (ANC)) 1.5109 / L (no 14 days prior to the first dose of the study);
      - White blood cell count (WBC) 3.0109 / L and 15109 / L;
      - Lymphocyte count (LC) 0.5109 / L;
      - Platelet count (PLT) 100109 / L (no blood transfusion within 14 days prior to the first dose of the study);
      - Hemoglobin (Hb): 90 g / L;
   2. Blood biochemical
      - TBIL≤1.5×ULN；
      - ALT and AST 2.5 ULN;
      - ALP≤2.5 ULN
      - BUN and Cr 1.5 ULN with a creatinine clearance of 50 mL/min (Cockcroft-Gault formula);
      - INR and APTT 1.5 ULN (no anticoagulant therapy)
      - Upper limit of normal (TSH) thyroid-stimulating hormone (ULN); abnormal T3 and T4 levels, normal T3 and T4 levels can be included;
   3. heart function
      - Heart color ultrasound: LVEF 50%;
      - 12-lead ECG: 1 ° QT interval <470 ms.
5. Tissue samples for evaluation of PD-L1 (at least 5 white slices of tissue specimens);
6. birth control:

Female fertile subjects are required to have a negative serum pregnancy test within 72 hours before the first dose and agree to use a medically approved, highly effective method of contraception during the study and within 90 days after the last administration of study drug.

After consultation with the subject, the investigator or his / her designee selected the appropriate contraceptive measures for him and her partner from the following contraceptive methods and confirmed that the subject had known how to correctly and consistently use the contraceptive method. At the time points listed in the flow sheet, the investigator will inform the subject of the need for continuous, correct contraception. In addition, the subject should be informed of the cessation of the chosen contraception or of suspected or confirmed pregnancy.

Efficient contraception is used correctly alone or combined with other methods, with a failure rate of less than 1% per year.

Including the following types:

1) The intrauterine device is correctly placed;

2) Condoms are simultaneously combined with topical spermicide (i. e., foam, gel, film, cream or suppositories);

3) Bilateral tubal ligation / bilateral salpingectomy or bilateral tubal occlusion surgery (occlusion surgery has been proved to be effective by relevant instruments);

4) Men were sterilized by vasectomy.

1. Volunteer to join the study, signed informed consent, had good compliance and willingness to cooperate with follow-up.

## Exclusion criteria

Not to be included in any of the following conditions:

1. Tumor-related symptoms and treatment:
2. Patients with metastatic breast cancer or bilateral breast cancer;
3. Inflammatory breast cancer patients;
4. Having received any anti-tumor therapy within 12 months before signing the informed consent form, including targeted therapy, chemotherapy, radiotherapy, endocrine therapy, immunotherapy, biological therapy or tumor embolization;
5. Prior treatment with PD-1 / PD-L1 antibody, CTLA-4 antibody, or other therapies against PD-1 / PD-L1 and / or VE GF R inhibitors.
6. Concurrent disease / medical history
7. Any other active malignancies within 5 years prior to signing the informed consent. Cuable localized tumors, such as skin basal cell carcinoma, skin squamous cell carcinoma, superficial bladder cancer, cervical carcinoma in situ, can be enrolled;
8. Have undergone a major surgical procedure unrelated to breast cancer within the 4 weeks before enrollment, or the patient has not fully recovered from such surgical procedure (tissue biopsy for diagnosis and central venous catheter placement through peripheral venous puncture [PICC] are allowed);
9. Subjects with any known or suspected autoimmune disease except: hypothyroidism due to autoimmune thyroiditis requiring only hormone replacement therapy; stable type I diabetes with blood glucose control;
10. Hypertension that could not be well controlled with antihypertensive medication (systolic> 140 mmHg or diastolic> 90 mmHg); within 6 months prior to enrollment, the following conditions occurred: myocardial infarction, severe / unstable angina, grade NYHA 2 or above cardiac dysfunction, clinically significant supraventricular or ventricular arrhythmia, and symptomatic congestive heart failure;
11. Presence of interstitial lung disease, noninfectious pneumonia, or uncontrollable systemic diseases (such as: diabetes mellitus, pulmonary fibrosis, and acute pneumonia, etc.);
12. History of live attenuated vaccination within 28 days before the first study administration or vaccination during the expected study;
13. Human immunodeficiency virus (HIV) infection or known acquired immunodeficiency syndrome (AIDS); active hepatitis B (defined as positive test for hepatitis B virus surface antigen and positive for HBV-DNA); hepatitis C (defined as HCV-RNA above the lower limit of detection of the analytical method) or combined co-infection with hepatitis B and hepatitis C; autoimmune hepatitis;
14. Severe infection within 4 weeks prior to first dose, including, but not limited to bacteremia, severe pneumonia requiring hospitalization; or active infection with CTCAE grade 2 within 2 weeks prior to first dose, or unexplained fever> 38.5°C during screening / before first dose (according to the investigator); evidence of active TB infection within 1 year prior to administration;
15. Subjects with previous or ready allogeneic bone marrow transplantation or solid organ transplantation;
16. Peripheral neuropathy grade 2;
17. There is a clinically significant intestinal obstruction;
18. Arteriovenous thrombotic events occurring within 3 months before enrollment, such as cerebrovascular accidents (including temporary ischemic attacks), deep venous thrombosis and pulmonary embolism;
19. Subjects with hemoptysis within 2 months before enrollment and a maximum daily hemoptysis of 2 ~ 2.5 mL. Within 3 months before enrollment, there were significant bleeding symptoms or clear bleeding tendency, such as gastrointestinal bleeding, hemorrhagic gastric ulcer, baseline fecal occult blood + + or above, or known genetic or acquired bleeding and thrombosis tendency, such as hemophilia, coagulation skills disorder, thrombocytopenia, hypersplenism, etc.;
20. Abnormal coagulation (INR> 1.5 or APTT> 1.5 ULN), with bleeding tendency or being on thrombolysis or requiring long-term anticoagulation with warfarin or heparin, or requiring long-term antiplatelet therapy (aspirin 300 mg / day or clopidogrel 75 mg / day).
21. Study treatment-related
22. Subjects who had received systemic immune stimulants (including but not limited to interferon or interleukin-2, including those in the clinical study phase) within 4 weeks prior to the first dose;
23. Subjects who had received systemic immunosuppressive agent therapy (including but not limited to glucocorticoids, azathioprine, methotrexate, thalidomide, anti-tumor factor agents) within 2 weeks prior to the first dose. Not including nasal spray and inhaled corticosteroids or physiological doses of systemic steroid hormones (i. e., no more than 10 mg/d of prednisone or other corticophysiological doses of equivalent drugs);
24. Known allergy to study drug or any of its excipients; or a severe allergic reaction to other monoclonal antibodies.
25. Female patients during pregnancy and lactation, female patients with fertility and positive baseline pregnancy test, or female patients of childbearing age who are not willing to take effective contraceptive measures throughout the trial;
26. A clear prior history of neurological or psychiatric disorders, including epilepsy or dementia, and a known history of psychotropic substance abuse, alcohol abuse, or drug use;
27. The patient was not considered fit by the investigator to participate in any other circumstances of the study.

## Subjects withdrew from the study or terminated the study treatment

### Exit from the study

Reasons for subject withdrawal from the study may include:

- The subject withdrew his / her informed consent to participate in the study and refused further follow-up;
- A clinical adverse event, abnormal laboratory tests or concurrent disease, which the investigator believes is not in the best interest of the subject;
- Other situations that the investigator considers necessary to withdraw from the study, such as the subject's ability to freely express his willingness due to imprisonment or isolation;
- Lost visit;
- Subjects died;
- The Sponsor has terminated the study.

The reason for subject withdrawal must be documented in the case report form and the subject's medical record.

It should be noted that withdrawal of informed consent means that the subject withdrew his consent to further contact, or ceased agreeing to give further information to the previously authorized person. Whenever possible, the subject should notify the investigator in writing that he has decided not to consent to follow-up. The investigator shall try to explain and document the withdrawal of informed consent to receive the study drug or to comply with the visit specified in the study protocol.

### Termination of the study standard of treatment

Termination of study treatment does not represent withdrawal from the study. Subjects who terminate the study treatment must continue to complete the remaining study visits as required by the protocol. Subject must terminate any study treatment if he meets any of the following criteria:

- The subject asked to terminate the study medication;
- Medical imaging or clinical features indicate disease progression, unless the subject meets the criteria for continued treatment after progression;
- The event of pregnancy during the study;
- Any clinical adverse events, laboratory abnormalities or other medical conditions may allow the subjects to continue the medication;
- Overall deterioration of health status and inability to continue participation in the trial;
- Significant protocol deviations were found after enrollment;
- Lost visit;
- The Sponsor terminates the study;
- Subjects died;
- Other reasons that the investigator considered unable to continue the study treatment.

### Steps for withdrawing from the study or terminating the study treatment

Efforts must be made to complete the efficacy and safety checks specified in the protocol, to complete the safety follow-up period, and to fully record the adverse events (AEs) and outcomes. The investigator may recommend or provide new or alternative treatments to the subject based on the actual situation. Non-disease progression subjects should continue to follow-up as much as possible for radiographic evaluation until the subject begins new antitumor therapy or disease progression.

Unlike subjects who withdraw their consent to withdraw from the study, subjects who request to terminate study treatment will be retained in the trial and should be followed in accordance with the study procedures specified in the protocol.

If the subject refuses to make further visits, they should continue as far as possible unless the subject withdraws further information or consent to be contacted. In this case, no further research evaluation and no more data should be collected.

## Remove the standard

- Do not meet the inclusion criteria, and meet the exclusion criteria;
- Insufficient data affects the judgment of efficacy and safety;
- Failure to use the drug according to the dose, method and course of treatment specified in the protocol shall affect the judgment of the drug efficacy;
- Use of the prohibited drugs as prescribed in the protocol.

## The study was prematurely terminated or suspended

The study may be terminated prematurely or suspended for sufficient justification. This may be due to the decision of the regulatory body, the change of opinion of the ethics committee, to the efficacy or safety concerns of the study drug, or in the judgment of the principal investigator. The party who decides to suspend / terminate the study will give a written notice and record the reason for the study termination or suspension to the investigator. The investigator shall notify the EC immediately and provide the relevant reasons.

**Early Termination site:**

- If the principal investigator finds serious or persistent noncompliance with the protocol and other trial procedures of the principal investigator, the right to terminate the proper conduct of the trial.
- If the investigator concludes or suspended the trial at the site, the subject must be immediately informed, and reported in writing to the site / ethics committee of the site as required by regulations.

**Reasons for early termination or suspension of the entire study may include:**

- Unexpected, significant, or unacceptable risks to the subject.
- Existing efficacy results support the early termination of the study.
- Major errors were found during the execution of the test.
- Study drug / trial treatment is ineffective, or continuing the trial is meaningless.
- It was extremely difficult to complete the trial due to reasons such as severe lag in subject inclusion or frequent protocol deviations.

Once the aforementioned drug safety, protocol compliance, data quality issues causing the study suspension are resolved and agreed by the principal investigator, the study may be continued.

## Definition of the end of the study

The end of the trial definition includes:

- The last 1 subject received surgical treatment and obtained the results of pathological evaluation.
- When the principal investigator decides to terminate the study.

# Research medication

## Drug allocation

After the subject signs a written informed compassion letter and completes the necessary baseline assessment, a subject number is automatically assigned that will be used for all CRFs and study documents. After the completion of the screening process, the patient will be formally entered into the study.

## Supply of the study drug

### dosage form and packaging

**Camrelizumab for injection**

Manufacturer: Suzhou Shengdiya Biomedical Co., Ltd.

Dosage form: lyophilized powder.

Specification: This product is 200 mg, packaged in 20 ml Xilin bottle.

Batch number: see the drug test report for details.

Route of administration: i. v.

Expiry date: from the production date, the shelf life is tentatively set for 2 years.

Storage conditions: sealed away from light, storage should be placed in a 2-8 °C refrigerator, do not freeze this product.

**Apatinib mesylate**

Manufacturer: Jiangsu Hengrui Pharmaceutical Co., Ltd.

dosage form: tablet.

Specification: 250mg / tablet.

Batch number: see the drug test report for details.

Route of administration: oral after meals (at the same time as possible).

Expiry date: 2 years.

Storage conditions: shading, sealing, storage below 25 °C.

**Paclitaxel for injection (albumin-bound type)**

Manufacturer: Jiangsu Hengrui Pharmaceutical Co., Ltd.

Formulform: This product is white to yellow lyophilized block or powder.

Specification: 100mg / box

Batch number: see the drug test report for details.

Route of administration: the intravenous route.

Expiry date: 2 years.

Storage conditions: shading, seal, and save below 20-30 °C.

**Epirubicin together with the cyclophosphamide**

See local prescription information for drug name and specifications

Labelling and packaging of each study drug are subject to the actual packaging.

The label of the test drug will be conducted in accordance with the guidelines of Good Clinical Practice (GCP), and the contents of the label shall include, but not limited to the study name, drug name, drug name, drug number, packaging specification, production batch number, expiration period, usage and dosage, drug storage conditions, and indicate the words "for clinical research only".

### Drug preparation, dispensing, and recovery

The preparation, distribution and recovery of the study drugs are under the responsibility of each site GCP pharmacy and the Investigator must ensure that all the study drugs are used only for subjects participating in this study and their dose and usage are prepared according to the drug manual and the latest market available instructions. The remaining study drug needs to be returned to the study drug provider. The expired or remaining liquid medicine shall be destroyed directly according to the medical waste standards.

Study drug was kept as labeled. Study drug receipt form must be signed in duplicate upon arrival at the study site, one each for the clinical study unit and the study drug provider. If the remaining drugs and empty boxes need to be recovered at the end of the study, both parties shall sign the study drug recovery form. The distribution and recovery of each study drug should be timely recorded on a special record sheet.

The monitor is responsible for monitoring the supply, use, storage of the study drug and the handling of the remaining drug.

The study drug can be destroyed by the study drug provider or by the study site. Before commissioning the site to destroy drugs, the monitor needs to confirm the site destruction process and provide relevant destruction certificates after destruction.

## Use methods of the study drugs

The study drugs Camrelizumab and albumin paclitaxel are used by intravenous infusion, which must be administered in the outpatient department or ward of the study center by qualified or experienced researchers, and should not be taken out of the study center for use.

The study drug must be used under the guidance of physicians with experience in the application of chemotherapeutic drugs. Due to the possibility of severe allergic reactions, appropriate emergency facilities should be available, and major safety indicators are recommended to be closely monitored during injection.

The drug configurations of Camrelizumab and albumin paclitaxel are provided in the drug instructions and IB manual.

No subject allergy pretreatment is required before the first dose of Camrelizumab. During the subsequent administration period, if any infusion reaction occurs, the subject shall be given the allergy drug pretreatment according to the infusion reaction treatment recommendations.

Before the first administration of albumin paclitaxel. During the subsequent administration period, if there is any allergic reaction, the subject should be pretreated according to the allergic reaction treatment recommendations.

The following drug use regimen was recommended for initial treatment in all trial subjects:

**Camrelizumab Injection:**

200 mg every 2 weeks, Cycles 1-4 on Days 1,15, Cycle 5-8 for approximately 30min (no less than 20min, no more than 60min). No dose increase or decrease is allowed, delay / interruption, delay / interruption maximum 12 weeks, calculated from the last dose, otherwise treatment was terminated.

**Apatinib tablets:**

250mg qd orally, about half an hour after meals (the time of daily medication should be the same as far as possible), with warm boiled water, take 8 consecutive treatment cycles, if missing is not allowed to fill. No dose increase or downregulation is allowed, delay / interruption is 28 days, calculated from the last dose, otherwise treatment was terminated.

**Albumin-Paclitaxel Injection:**

Every 4 weeks is one treatment cycle, cycles 1-4 for 4 cycles; 100mg / m2 albumin on days 1,8,15, IV infusion, approximately 30min. Dose down and delayed dose were allowed, with a maximum of 3 weeks, calculated from the last dose time, otherwise treatment was terminated.

**epirubicin:**

Every 2 weeks was a treatment cycle, cycles 5-8 for 4 cycles; 90mg / m2 epirubicin, intravenous infusion, for approximately 30min. Dose down and delayed dose were allowed, with a maximum of 3 weeks, calculated from the last dose time, otherwise treatment was terminated.

**cyclophosphamide:**

Every 2 weeks was one treatment cycle, cycles 5-8 for 4 cycles; 600mg / m2 cyclophosphamide was administered on day 1 of each cycle, with intravenous infusion of approximately 30min. Dose down and delayed dose were allowed, with a maximum of 3 weeks, calculated from the last dose time, otherwise treatment was terminated.

**Order of the combination of drug administration:**

Camrelizumab was given on day 1 of each cycle, at least 30min apart.

The subject should continue study treatment until the end of the course, disease progression, toxicity intolerance, active request for study treatment termination or withdrawal from the study, or the investigator determines the subject to withdraw from the study.

## Administration interruption and dose adjustment

### Overall dose adjustment principle

Immune checkpoint inhibitors and chemotherapy and antiangiogenic targeted drugs have different mechanisms of action, and large differences in toxicity profiles, so there is no evidence of significant mutual influence. Therefore, if the investigator determines that the toxicity of the subject is related to the drug but not the other, the dose of the part of the drug related to toxicity can be adjusted, and the interruption / delay / termination of the part of the drug related to toxicity. If it is not determined whether it is only related to some drugs, the whole study drugs will be adjusted.

The efficacy of antitumor drugs is continuous, interrupt / delaying treatment within the prescribed time limit, and no evidence of significant reduction in efficacy. Therefore, subjects only interrupt / delay the treatment of some drugs related to toxicity, until the toxicity basic recovery all drug treatment as planned, avoid disrupting the combination treatment plan, for each drug cycle given within the prescribed time window, for card and albumin paclitaxel two intravenous drugs, try to ensure that on the same day within the specified time window.

The reasons for dose adjustment or delayed administration, and the symptomatic treatment measures and recovery results should be recorded in the medical record and eCRF. The grade of adverse events was graded according to NCI CTCAE v5.0.

Overall, the start of each cycle can be delayed due to no recovery of toxicity, and administration at the treatment window (+ 3 days) in each cycle does not count as delayed treatment, and administration beyond the treatment window (+ 3 days). For cases not specified in the protocol, the benefit / risk ratio of the subject. Once the dose is reduced, it cannot be adjusted back.

If the chemotherapy regimen albumin paclitaxel, epirubicin and cyclophosphamide cannot meet the requirements of chemotherapy for more than 3 weeks, it will be permanently stopped. If Camrelizumab is stopped for more than 12 weeks, it will be stopped permanently. If apatinib is not stopped for more than 28 days, it will be stopped permanently.

### Interruption of Camrelizumab administration with dose adjustment

Camrelizumab is administered at a fixed dose and does not allow to increase or decrease the dose of Camrelizumab if the subject delays or breaks Camrelizumab treatment due to toxicity when the toxicity improves within the prescribed time limit. A delay / interruption was allowed up to 12 weeks, calculated from the last dose, otherwise treatment was terminated. For subjects who returned to baseline after 12 weeks, the decision to resume treatment with Camrelizumab is based on the benefit-risk ratio judged by the investigator.

Cycles 1 to 4 are a fixed treatment cycle every 4 weeks and 2 weeks for cycles 5 to 8, and the time window of each dose was calculated from the date of the first dose of each cycle, allowing no delay in the overtreatment window (+ 3 days) during the cycle. In case of a delay (3 days), the next dose window, the next cycle dose window, as planned. If D15 administration is delayed for more than 3 days, then D15 administration is skipped, the dose will not be replaced in this cycle, and the drug will continue in the next cycle administration time window.

If subjects are allowed to interrupt medication due to other medical or surgical procedures, or unexpected (holiday) events not related to the study treatment, subjects should resume study treatment within 3 weeks of the planned administration date, unless other resolution is discussed with the principal investigator. The reason for the treatment interruption must be recorded in the eCRF.

Adverse events associated with Camrelizumab may be immunotoxicity, possibly within a short time after the first dose or months after the last dose. In the cases listed in Table 2 below, delay or interruption of Camrelizumab therapy, if the investigator considers the benefit / risk ratio or during the clinical procedure, should be discussed with the principal investigator.

| **Ta****ble 2 Dose adjustment provisions of Camrelizumab due to immune-related toxicity** | | | |
| --- | --- | --- | --- |
| Toxicity associated with Camrelizumab | Level of treatment suspension  (NCI CTC v5.0) | Re-start treatment  The time of | Permanent withdrawal of drugs |
| Diarrhea / colitis | Level 2-3 | The toxicity was reduced to grade 0-1 | Prednisone (or equivalent) with toxicity not resolved within 12 weeks after the last dose or failed to reduce corticosteroid dosage to 10 mg / day or less within 12 weeks |
|  | Level 4 | Permanent withdrawal of drugs | Permanent withdrawal of drugs |
| ^The AST, ALT, or bilirubin was increased a^ | Level 2 | The toxicity was reduced to grade 0-1 | Prednisone (or equivalent) with toxicity not resolved within 12 weeks after the last dose or failed to reduce corticosteroid dosage to 10 mg / day or less within 12 weeks |
|  | Level 3-4 | ^Permanent withdrawal of drug A^ | Permanent withdrawal of drugs |
| Type I diabetes mellitus (e. g., a new onset) or  Hyperglycaemia associated with signs of islet β -cell exhaustion | Grade 3-4 or new onset of type I diabetes mellitus | Use of SHR-1210 should be suspended for new onset type 1 diabetes or grade 3-4 elevated glucose with evidence of impaired beta cells | Camrelizumab therapy may be restarted after the subject has a stable clinical and metabolic status. |
| hyperthyrea | Level 3 | The toxicity was reduced to grade 0-1 | Prednisone (or equivalent) with toxicity not resolved within 12 weeks after the last dose or failed to reduce corticosteroid dosage to 10 mg / day or less within 12 weeks |
|  | Level 4 | Permanent withdrawal of drugs | Permanent withdrawal of drugs |
| hypothyrea | After starting thyroid hormone replacement therapy, the treatment can be continued | | |
| pneumonia | Level 2 | The toxicity was reduced to grade 0-1 | Prednisone (or equivalent) with toxicity not resolved within 12 weeks after the last dose or failed to reduce corticosteroid dosage to 10 mg / day or less within 12 weeks |
|  | Level 3-4 | Permanent withdrawal of drugs | Permanent withdrawal of drugs |
| hypophysitis | Level 2-4 | Toxicity reduced to grade 0-1; endocrine replacement therapy may be continued after initiation | Prednisone (or equivalent) with toxicity not resolved within 12 weeks after the last dose or failed to reduce corticosteroid dosage to 10 mg / day or less within 12 weeks |
| infusion reaction | ^Level 2 b^ | The toxicity was reduced to grade 0-1 | If the symptoms relapse after adequate prophylaxis, the drug is permanently discontinued. |
|  | Level 3-4 | Permanent withdrawal of drugs | Permanent withdrawal of drugs |
| Renal failure or nephritis | Level 2 | The toxicity was reduced to grade 0-1 | Prednisone (or equivalent) with toxicity not resolved within 12 weeks after the last dose or failed to reduce corticosteroid dosage to 10 mg / day or less within 12 weeks |
|  | Level 3-4 | Permanent withdrawal of drugs | Permanent withdrawal of drugs |
| ^Other drug-related toxicities, c^ | Level 3 | The toxicity was reduced to grade 0-1 | Prednisone (or equivalent) with toxicity not resolved within 12 weeks after the last dose or failed to reduce corticosteroid dosage to 10 mg / day or less within 12 weeks |
|  | Level 4 | Permanent withdrawal of drugs | Permanent withdrawal of drugs |

Note: Repeat any serious or grade-3 (pneumonia-2) or drug-related AEs, or any life-threatening AE.

A For subjects with Grade 2 AST or ALT elevated at baseline, if AST or ALT increases 50% from baseline for at least 1 week.

1 b If the symptoms resolve within 1 hour of temporary withdrawal, the infusion can be continued at 50% of the initial infusion rate. Otherwise, you must wait for complete remission of symptoms before remedication. The subject should also be given adequate prophylactic medication at the next dose. For further treatment, see Table 5 for recommendations for infusion reaction handling.

1 c For subjects with intolerable or persistent grade 2 drug-related AEs at investigator discretion, and for subjects with persistent Grade 2 drug-related AEs, medication should be permanently stopped if the toxicity fails to decrease to level 0-1 within 12 weeks after the last dose.

### Interruption of albumin-paclitaxel administration with dose adjustment

The dose of each chemotherapy preparation was calculated based on the body surface area of the subject. Baseline weight and height should be recorded and weight at each specified visit. If the baseline weight was> 10%, the dose was recalculated. If the dose is recalculated due to a> 10% change from baseline of body weight, this weight will be used as a new baseline to calculate the chemotherapy dose for subsequent courses.

Overall, the absolute value of neutrophils must be 1500 / μ L and platelets be 100000 / μ L before each dose. During the treatment period, the patient with severe neutropenia (ANC <500 / μ L for 1 week or more) or 3 degrees of sensory neurotoxicity should be suspended until the absolute neutrophil value returns to 1500 / μ L and neurotoxicity to 2 degrees, and the treatment dose is reduced to 100mg / m2. For such severe neutropenia or sensory neurotoxicity, the subsequent therapeutic dose should be reduced to 75mg / m2. No dose adjustment for grade 1 or 2 peripheral neurotoxicity. Up to two dose downdoses were allowed per patient, and the above severe neutropenia or 3 degree sensory neurotoxicity after the second downdose. If the delay to restoring albumin paclitaxel is greater than 7 days (e. g., until D15 neutrophil and platelet counts), the dose of albumin paclitaxel should also be reduced according to Table 3. Delay or interruption of dose was allowed for up to 3 weeks, calculated from the last dose, otherwise treatment was terminated. For subjects whose toxicity returned to baseline beyond 3 weeks, the sponsor may discuss whether to resume treatment with nab paclitaxel based on the benefit-risk ratio judged by the investigator.

**Table 3 Dose adjustment regulations of albumin paclitaxel due to hematologic toxicity**

| hematologic toxicities | Frequency of occurrence | Weekly paclitaxel dose (mg / m2) |
| --- | --- | --- |
| Defic fever (ANC <500 / μ L and axillary temperature T> 38 degrees)  perhaps  Albumin paclitaxel was delayed> 7 days before D1 use due to ANC <1500 / μ L  perhaps  The ANC <500 / μ L was greater than 7 days | for the first time | 100 |
|  | second time | 75 |
|  | third time | TD |
| Platets <50000 / μ L | for the first time | 100 |
|  | second time | TD |

The time interval of albumin paclitaxel administration should be no less than 7 days, every 4 weeks. From the time of first dose, the in-period delay dose window will generally not exceed 3 days. If there is a delay (3 days), the next medication time will be recalculated with the actual dose time, and the subsequent dose time window will remain 3 days. If D8 is delayed (> 3 days), skip directly to D15. If the delay of D15 is more than 3 days, the next dose time, i. e. in the next cycle, is recalculated with the actual dose time, and the window period is still 3 days.

Albumin paclitaxel has been marketed, and dose adjustment caused by other adverse reactions during treatment can be treated by the investigator by referring to the latest instructions.

### Apatinib administration was adjusted

Dose adjustments due to apatinib-related toxicity included: dose delay (up to 28 days) and permanent termination of treatment.

Haematological toxicity grade 3 or non-haematological toxicity grade 2 requires suspension; for non-haematological toxicity, such as controllable nausea, vomiting, and fever with established cause (below 38°C), aggressive symptomatic treatment may be performed first without immediate administration delay.

In case of apatinib-related toxicity, the administration should be delayed at the original dose (250 mg, qd). The recommended dose adjustment method is shown in Table 4. An increase or reduction of apatinib dose was not allowed during the study.

**Table 4 Principles of Dose Adjustment of Apatinib (NCI-CTCAE5.0)**

| **Apatinib-related toxicity** | **classify** | **Whether dosing is suspended** | **Reinstatement of administration criteria** | **Termination of medication criteria** |
| --- | --- | --- | --- | --- |
| **hematologic toxicities** | Level 1-2 | deny | - | - |
|  | Level 3-4 | yes | To allow the toxicity to return to grade 2 | Apatinib was stopped for over 28 days |
| **^Non-hematologic toxicity a^** | Level 1 | deny | - | - |
|  | Level 2-3 | yes | To allow the toxicity to return to grade 1 | Apatinib was stopped for over 28 days |
|  | Level 4 | Termination of dosing | | |
| **^hypertension b^** | Grade 3 (corrected treatment) | yes | To allow the toxicity to return to grade 1 | Apatinib was stopped for over 28 days |
| **Proteinuria (without a significant increase in blood creatinine)** | Grade 3 (24-hour urine protein quantification) | yes | To allow the toxicity to return to grade 1 | Apatinib was stopped for over 28 days |
| **^hand-foot syndrome c^** | Level 3 | yes | To allow the toxicity to return to grade 1 | Apatinib was stopped for over 28 days |
| **headache** | Grade 2 (for 7 days) or Grade 3 | yes | To allow the toxicity to return to grade 1 | Apatinib was stopped for over 28 days |

1. The following recommendations are provided for bleeding, cardiotoxicity, and liver toxicity:

- Grade 3-4 apatinib-related bleeding: Suspension is recommended and continued when return to grade 1; stop if repeat Grade 3-4 bleeding.
- Grade 3-4 apatinib-related cardiotoxicity: Suspension is recommended; continue if return to grade 1; but stop if repeat Grade 3-4 cardiotoxicity; stop subjects with grade III-IV cardiac insufficiency or LV ejection fraction <50%.
- Grade 3-4 apatinib-related liver toxicity: ALT / AST and / or total bilirubin increased, suspension is recommended, while monitoring serum transaminase total bilirubin level until grade 1; terminate the grade 3-4 liver toxicity again.

1. Correction treatment for apatinib-related grade 4 hypertension should be terminated.
2. For three consecutive episodes of Grade 2 apatinib-related hand-foot syndrome, recommended to be administration; if Grade 2 if if again

If apatinib is suspended for more than 28 days and the toxicity does not recover, apatinib treatment will be terminated unless the investigator considers the continuation of apatinib treatment beneficial to the subject and decided after discussion with the Sponsor.

Apatinib should be permanently discontinued in patients with gastrointestinal perforation, requiring clinical wound dehiscence, fistula, severe bleeding, nephrotic syndrome, or hypertensive crisis.

### Epirubicin with cyclophosphamide administration was interrupted with dose adjustment

The dose of each chemotherapy preparation was calculated based on the body surface area of the subject. Baseline weight and height should be recorded and weight at each specified visit. If the baseline weight was> 10%, the dose was recalculated. If the dose is recalculated due to a> 10% change from baseline of body weight, this weight will be used as a new baseline to calculate the chemotherapy dose for subsequent courses.

The administration of epirubicin combined with cyclophosphamide should be no less than 14 days apart, with a fixed cycle for every 2 weeks. Starting from the first dose time, the next dose time is recalculated with the actual dose time.

Since this phase is a dose-intensive regimen, the absolute value of neutrophils must be 1500 / μ L and platelets be 100000 / μ L before each dose. During the treatment period, if patients develop severe neutropenia (ANC <500 / μ L for 1 week or more), the administration should be suspended, and the treatment should not be continued until the absolute value of neutrophils is restored to 1500 / μ L. In order to ensure efficacy, the prophylaxis of G-CSF (secondary prevention) is recommended, and the treatment dose should be reduced to 75mg / m2 in the subsequent course. If the above severe neutropenia occurs again, the subsequent therapeutic dose should be reduced to 50mg / m2. Up to two dose reductions were allowed per patient, and if the subject repeated the above severe neutropenia after the second dose reduction, the anthracycline plus cyclophosphamide chemotherapy was terminated. If the delay to recovery of anthracycline with cyclophosphamide is greater than 7 days (e. g. until the D15 neutrophil and platelet count), the dose should also be tapered as per Table 5. Delay or interruption of dose was allowed for up to 3 weeks, calculated from the last dose, otherwise treatment was terminated. For subjects who returned to baseline after 3 weeks, the return of epirubicin with cyclophosphamide was determined after the benefit to risk ratio judged by the investigator.

Management of anthracycline-related cardiotoxicity is shown in Figure 1. Schematic diagram of handling when asymptomatic LVEF decreases.

**Table 5 Provisions for dose adjustment of chemotherapy due to hematologic toxicity**

| hematologic toxicities | Frequency of occurrence | epirubicin  Dose of (mg / m2) | cyclophosphamide  Dose of (mg / m2) |
| --- | --- | --- | --- |
| Defic fever (ANC <500 / μ L and axillary temperature T> 38 degrees)  perhaps  Delay of> 7 days for ANC <1500 / μ L before D1 use in each cycle  perhaps  The ANC <500 / μ L was greater than 7 days | for the first time | 75 | 450 |
|  | second time | 50 | 300 |
|  | third time | TD | TD |
| Platets <50000 / μ L | for the first time | 75 | 450 |
|  | second time | TD | TD |


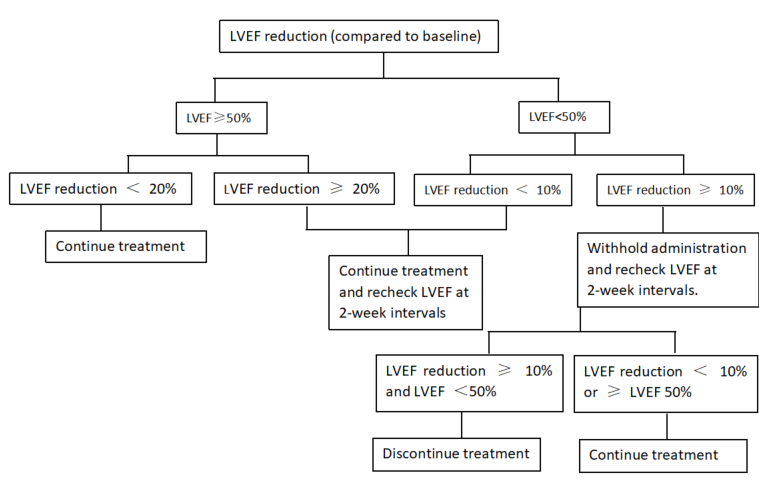


**Figure 1. Schematic diagram of the treatment when the asymptomatic LVEF drops**

## Symptomatic treatment of common adverse reactions of Camrelizumab

### Safety management rules of immunooncology drugs

The toxicity of immune checkpoint inhibitors (immune checkpoint inhibitors, ICPi) can be divided into infusion reactions and immune-related adverse events (immune-related adverse events, irAE), with irAEs most frequently occurring in the skin, colon, endocrine organs, liver and lung. Other organs and tissues, although rare, may be relatively more serious and even life-threatening, such as neurological lesions and myocarditis.

Camrelizumab is one of ICPi, but toxicity varies between different immune checkpoint inhibitory drugs. The treatment of related adverse events can be referred to the Guidelines for the Toxicity Management of Immunotherapy: Clinical Practice for the Diagnosis, Treatment and Follow-up of ESMO [22] and Guidelines for the Toxicity Management of Immunocheckpoint Inhibitors 2021 Edition [23]. The guidelines applies to all immune checkpoint inhibitors.

Early identification and treatment of adverse events occurred during the study to reduce the occurrence of serious toxic events.

### infusion reaction

Potential infusion reactions and / or allergic reactions, especially acute immune-mediated adverse reactions (including cytokine storm), will require close attention throughout the study.

Since Camrelizumab is a fully humanized monoclonal antibody and is less likely to have infusion or allergic reactions, preventive medication is generally not needed before the infusion of Camrelizumab. Based on the published information, the most likely allergic reaction / allergic event occurred within 24 hours of the infusion. If it occurs, the infusion should be slowed down or interrupted according to the situation, clinical support treatment should be given, and prophylactic medication should be given before later medication. Possible allergic reactions include fever, chills, chills, headache, rash, arthralgia, low or hypertension, or bronchospasm.

Management of anaphylaxis should be performed according to the medical practice and guidelines of the research institution. The following are the treatment recommendations for infusion reactions (Table 6).

**Table 6 Treatment recommendations for Camrelizumab infusion reactions**

| CTCAE rank | clinlcal symptom | Recommended processing | Camrelizumab therapy |
| --- | --- | --- | --- |
| Level 1 | Mild transient response | Bedside observation and closely monitored until recovery. Pre-infusion prophylaxis is recommended: 50 mg, or equivalent and / or acetaminophen 325-1000 mg at least 30 min before SHR-1210. | Continue to use |
| Level 2 | Moderate response, requiring treatment or suspension, and can be quickly relieved after symptomatic treatment (such as antihistamines, nonsteroidal anti-inflammatory drugs, anesthetics, bronchodilators, intravenous fluids, etc.). | Normal saline intravenous infusion, diphenhydramine 50 mg IV or equivalent and / or acetaminophen 325-1000 mg; observed at the bedside and monitored closely until recovery.  Corticosteroids or bronchodilators may be considered as clinical needs;  Original medical record records the amount of study drug infusion;  Pre-infusion prophylaxis is recommended: 50 mg, or equivalent and / or acetaminophen 325-1000 mg at least 30 min before SHR-1210. If necessary, cortisolhormone (hydrocortisone dose equivalent to 25 mg) may be used. | suspend. When re-medication after symptom resolved, an initial infusion speed of 50% was used. If there are no complications within 30 minutes, it can return to the original infusion rate.  Watch closely. If symptoms recur, no infusion was given. |
| Adventitia level 3 | Grade 3: severe response, no rapid response after treatment and / or suspension; or recurrence of symptoms after remission; sequelae requiring hospitalization.  Level 4: a life-threatening condition. | Immediately stop the infusion of SHR-1210;  An intravenous infusion of normal saline was started.  A bronchodilator is recommended, subcutaneous 1:1000 adrenaline solution 0.2-1 mg or 0.1-0.25 mg of 1:10000. If necessary, intravenous diphenhydramine 50 mg and methylprednisolone 100 mg (or equivalent dose).  Follow the institutional medical practices and guidelines for the treatment of anaphylaxis. Bedside observation and closely monitored until recovery. | Termination of medication. |

## Dose tracking

The site will complete study drug preparation and record as required above.

The clinical research site documentation system shall include all relevant or required information for drug preparation and administration.

## Study drug storage

The Investigator, or his authorized representative (e. g. pharmacist), will ensure that all study drugs (including Camrelizumab, apatinib, albumin paclitaxel) are stored in a controlled, safe area and in accordance with applicable regulatory requirements.

Camrelizumab, apatinib and albubupaclitaxel should be stored in their original containers and consistent with the drug label. Once Camrelizumab and albumin paclitaxel products are prepared or diluted, refer to the storage conditions of the drug package insert.

If the storage conditions on the label and other data of the study drug (e. g. IB) are inconsistent, the storage conditions on the label shall prevail.

The site must be able to record the daily maximum and minimum temperatures at all storage locations (e. g., frozen, refrigerated or room temperature). The recording period shall start with the receiving product until the end of the trial. Even with a continuous monitoring system, the site shall log to ensure correct storage temperature. Temperature monitoring devices and storage devices (e. g., refrigerator) should be checked regularly to ensure proper operation.

Once any deviation from the product label conditions occurs, it shall be reported in time. The site should take active steps to return the product to the storage conditions described in the label as soon as possible, and also report the temperature deviations and actions taken to the study drug provider.

Study drug affected by the temperature deviation should be temporarily isolated until permitted by the study drug provider, which is not a protocol deviation. Continuing use of the affected study drug without the permission of the study drug provider is a protocol deviation. The study drug provider will provide specific steps for the reporting temperature deviation.

For home administration studies, the site staff will guide the subjects on the correct way to store the drug.

Refer to the study drug manual for other storage guidance and actions to be taken when stored outside the specified conditions.

## MAR

The site must maintain good records of study medication supply, including receipt, distribution, use, recovery, loss, or other whereabouts. In cases where trial drug has been distributed to individual subjects, these subjects should be required to return the unused trial drug and packaging when they return for each subsequent visit. Subjects recorded their medication information in a daily diary.

## Concomitant medication and concomitant therapy

Concomitant medication / concomitant treatments are other drugs / treatments given to the subject at the discretion of the investigator.

From the 30 days before the end of the study safety visit, all concomitant medication, blood products and non-drug intervention of participants should be recorded in the case report form in strict accordance with the provisions of the GCP.

Concomitant medication records should include drug name, drug dose, route of administration, frequency of administration, purpose of administration, and start and end dates. If the subject initiated new systemic systemic anti-tumour therapy during safety follow-up, only concomitant medication / concomitant therapy for adverse events related to the study drug were recorded. Once the subject interrupted trial treatment, only concomitant medication and concomitant treatments for new or unresolved adverse events related to trial treatment should be recorded. All of the above concomitant medication and concomitant treatments must be recorded in the subject's original records and the corresponding portion of the electronic case report forms (eCRFs).

Drugs or vaccines that are explicitly prohibited in the study protocol are prohibited throughout the study. If the subject has a complicated disease that must use the prohibited drug, the study drug treatment may need to be stopped or receive the prohibited drug, the investigator shall discuss with the sponsor. the subject to continue the study treatment or receive the prohibited drug shall be decided jointly by the investigator, the sponsor and the subject.

In addition to the above, subjects who receive Camrelizumab, apatinib mesylate, albumin paclitaxel, epirubicin, cyclophosphamide should also refer to the latest version of the marketed drug instructions, IB manuals, or requirements for contraindications and precautions in medical practice practices.

### Permissible concomitant medication use

Medications or treatments permitted during the study period include:

• Treatment of complications or adverse events, or symptomatic treatment (including blood products, blood transfusions, fluids, antibiotics, antidiarrheal drugs, etc.), except for those expected to interfere with the evaluation (or interaction) of the study treatment;

• antanacathartic;

• nutritional support;

Drugs or treatment required for previous diseases;

Adrencorticoid replacement therapy with an equivalent dose of 10mg / day prednisone;

For patients with infusion reaction after the first dose of Camrelizumab, they can subsequently receive prophylactic medication (antihistamine therapy, acetaminophen, corticosteroids or 5-HT 3 antagonist therapy, or prophylactic medication according to local diagnosis and treatment guidelines);

Granulocyte colony-stimulating factor, erythropoietin, or prothrombopoietin;

Postoperative analgesic use.

### Prohibited / restricted concomitant medication use

Medications or treatments prohibited during the study treatment period include:

Any other system with anti-tumor effect of chemotherapy, endocrine therapy, radiotherapy, immunotherapy, biological therapy, molecular targeted therapy or modern TCM treatment (including but not limited to rhinoceros yellow pill, golden dragon capsule, Kang Lai Te injection, eddy injection, Kang Ai injection and cancer tablet, etc., can refer to the tumor palliative care Chinese patent medicine using expert consensus (2013 edition) related classification and regulations), etc.;

Any other clinical trial drugs;

Immunosuppressive drugs include, but are not limited to, systemic corticosteroids, methotrexate, azathioprine, or their equivalent, tumor-necrosis factor- α (TNF- α) blockers, CTLA-4 inhibitors, etc. Exceptions to the following cases:

- Treatment treatment related AEs with immunosuppressive drugs;
- Short-term prophylaxis for subjects expected to receive chemotherapy, if drug prescription information requires corticosteroids in patients with documented hypersensitivity;
- In patients with contrast agent allergy;
- In addition, the use of inhaled, other local (ocular, intra-articular, nasal cavity) corticosteroids is also allowed;
- Short-term use of corticosteroids (for example, for control of chronic obstructive pulmonary disease, radiotherapy, nausea, etc.) is permitted.

Live vaccination within 28 days before the first use of the study drug and participating in the study process, including but not limited to: measles, mumps, rubella, chickenpox, yellow fever, rabies, BCG, typhoid vaccine. Allow receipt of inactivated virus vaccine against seasonal influenza, but not live attenuated influenza vaccine for intranasal use;

The use of immunomodulatory agents, including but not limited to interferon, IL-2, thymoin, thymus xin, immune cell therapy, are prohibited.

### surgical operation

Any surgery performed during the study should have its theoretical basis and necessity. The time interval between the operation and the study drug administration should not affect the wound recovery and the search of the unknown cause of bleeding. Recommendation of withdrawal of study drug 1 week before surgery depends on clinical evaluation of wound healing and postoperative recovery.

## Subjects adherence

Medication of the study treatment will be recorded in the eCRF both during the study and in the follow-up period. Any deviation from the medication specified in the study protocol will be recorded in the eCRF, including the date and reason of the medication. The Clinical Monitor (CRA) will review treatment compliance during the site visit and at the end of the study.

For i. v:

The site will complete medication preparation and record according to the study medication manual. The clinical research site documentation system shall include all relevant information for drug preparation and administration. These records include at least the following information:

• Record of drug delivery received (acceptance date and quantity)

The dispensing log must be recorded in real time, including the following information:

• Study number for the patients receiving the study drug

• Date, drug number, and quantity of the study drug issued to the patient.

Copies of the drug dispensing and inventory sheets must be kept. Destruction instructions for unused and partially used study drugs or packages are provided in Section 5.2.2.

# stages of research

## dressing by screening

After signing the informed consent form, the subject entered the screening period. Unless specifically noted, the following screening procedure must be completed within 28 days prior to initiation of study medication (see the Trial Flow Chart for details):

- Written informed consent was obtained signed from the subject.
- Demographic data were collected: gender, date of birth, nationality, height, weight, etc.
- Tumor diagnosis: the date of pathological diagnosis, pathological classification, pathological stage (TNM), and clinical stage.
- History of tumor treatment
  - History of tumor surgery: surgery name, date of surgery;
  - History of radiotherapy: site, dose, start and end date of radiotherapy.
  - History of concomitant diseases, past medication history, drug allergy history, etc.;
- Full physical examination: general condition, head and neck (including thyroid), chest (including heart, lung), abdomen (liver, gallbladder, spleen, kidney), limbs, skin, lymph nodes, genito-urological system, musculoskeletal, nervous system and mental state, etc.
- Vital signs: pulse rate, respiratory rate, body temperature, and blood pressure.
- ECOG grade.
- Blood routine: red blood cell count (RBC), hemoglobin (Hb), platelet count (PLT), white blood cell count (WBC), neutrophil count (ANC), lymphocyte count.
- Blood biochemistry: alanine aminotransferase (ALT), glutamate transaminase (AST), glutamyl transpeptidase (γ -GT), total bilirubin (TBIL), direct bilirubin (DBIL), alkaline phosphatase (ALP), blood urea nitrogen (BUN) or urea (preferably blood urea nitrogen), total protein (TP), albumin (ALB), lactate dehydrogenase (LDH), creatinine (Cr), blood glucose (GLU), K +, Na +, Ca 2 +, Mg2 + and Cl-.
- Urine routine: white blood cells, red blood cells, urine protein.
- Fecal occult blood (OB): if positive, it should be reviewed for fecal occult blood +. The investigator should perform gastrointestinal examination to exclude gastrointestinal bleeding.
- Coagulation function: including the international normalized ratio INR, activated partial thromboplastin time APTT, fibrinogen FIB, thrombin time TT; if the international normalized ratio INR is not available, then prothrombin time PT is used instead.
- Thyroid function: serum thyroid-stimulating hormone (TSH), free triiodothyronine (FT3), free thyroxine (FT4); if FT3 and FT4 are not available, T3 and T4 are replace.
- Virorology: HBsAg, HBsAb, HBeAg, HBeAb, HBcAb (qualitative, HBV DNA if HbsAg is positive), HCV-Ab (HCV-RNA), and HIV-Ab.
- Pregnancy test (for safety reasons, requiring all childbearing age female subjects for serum pregnancy tests): within 72 hours before the first dose and at the treatment termination visit.
- 12-lead ECG: Heart rate, QT, QTc, and P-R intervals should be indicated.
- Echocardiography: You need to indicate at least the left ventricular ejection fraction (LVEF). Performed within 28 days prior to the first dose.
- Imaging examination: According to the diagnosis and treatment principles of each study center, complete imaging examinations, such as head, neck, chest, abdomen, pelvic cavity and bone tissue (such as head CT / MRI, chest, upper abdomen, pelvic CT scan, systemic bone scan, etc.), CT / MRI results within 28 days before enrollment, and bone scan results within 42 days before enrollment can be used. Imaging findings obtained before signing informed consent can be used for screening tumor evaluation as long as they are met.
- Adverse Events: Adverse events were recorded from signing the informed consent form per NCI CTC AE v5.0 criteria.
- Concomitant / concomitant treatment: Concomitant / concomitant therapy was recorded within 30 days prior to the first study medication.

See the test flow chart for each inspection time point.

### Baseline tumor and lymph node status assessment

- Baseline tumor assessment will include clinical breast examination (CBE) (including breast / axillary / supraclavicular fossa), mammography and ultrasound, and breast magnetic resonance imaging (MRI) scans.
- Before neoadjuvant therapy, the tumor lesion was physically labeled using site standard clinical practices (such as skin tattoos or surgical clips) so that after complete tumor regression before surgery (see Annex VI for more details).
- Tumor tissue and peripheral blood samples were obtained before enrollment for the detection of biomarkers such as PD-L1.
- Baseline axillary lymph node status should be assessed from a clinical perspective based on each site's clinical practice and documented with the details found. For subjects with clinical and / or ultrasound suspected axillary lymph node metastasis at baseline, axillary staging should include fine needle aspiration (FNA) or hollow needle needle biopsy before neoadjuvant therapy.

## Enter the group

- Confirm the entry and arrangement criteria.
- Pregnancy test: Serum pregnancy test results within 72 hours prior to the first dose.
- Pre-dose laboratory tests (blood routine, blood biochemistry, urine routine, fecal occult blood, coagulation function, and thyroid function) and electrocardiogram tests, such as the corresponding baseline laboratory tests performed within 7 days before drug administration, may not be repeated.
- Subjects were administered.

## stage of therapy

Study treatment was initiated within 72 hours of enrollment.

The treatment period begins with the first dose. The first study medication should be as close as possible to the time to complete the screening period and confirm the entry criteria. Cycles 1-4 are every 4 weeks and cycles 5-8 are every 2 weeks.

All examinations and assessments (except imaging tests) should be completed within 3 days before administration, and laboratory tests (blood routine, blood biochemistry, urine routine, fecal occult blood, coagulation function, and thyroid function) and electrocardiogram tests, if not repeated within 7 days before administration.

Before the start of the new cycle, the subjects should go to the study site for the corresponding examination and evaluation:

- ECOG score: it is required to be performed during each cycle of follow-up;
- Vital signs: All signs are required at every cycle of follow-up;
- Body weight: it should be performed at each cycle of follow-up, and before each chemotherapy;
- Blood routine: it should be performed during each follow-up, before each chemotherapy;
- Urine routine: follow-up in each cycle; if urine routine shows urine protein + + or above, add urine protein quantification for 24h;
- Occult blood in stool: every cycle of follow-up;
- Blood chemistry: every cycle of follow-up; additional tests if necessary. Albumin paclitaxel before each chemotherapy; Camrelizumab before each treatment;
- Coagulation function: as required in every cycle of follow-up;
- Thyroid function: at every cycle of follow-up;
- 12-lead ECG: required at every cycle of follow-up; it can be repeated if necessary (at least 10 min interval).
- Cardiac color ultrasound: once at the beginning of C5D1; if the chest area during the study, pain, palpitations and other symptoms, as appropriate;
- Record of adverse events.
- Concomitant medications and concomitant treatments were recorded.
- Tumor imaging: Tumor assessment Visit After treatment begins, every 2 cycles of breast ultrasound or MRI evaluation. Subjects must undergo CBE, mammography and ultrasound and breast MRI scans after completion of neoadjuvant therapy (before surgery). During neoadjuvant therapy, in addition to the primary lesion, additional necessary imaging tests can be added according to the RECIST 1.1 criteria (see Annex III). If the lesion progsignificantly, the subject should discontinue study treatment and receive standard therapy at the study center. If contralateral ductal carcinoma is found during the course, it is not considered as PD. However, the contralateral invasive breast cancer is seen as a PD.
- Blood sampling: peripheral blood was collected once at the beginning, before and after C5D1. At least 7.5 mL of peripheral blood was collected each time.

## Surgical treatment after the completion of the neoadjuvant therapy

• Breast surgery treatment (the recommended time interval between breast surgery and the last administration of apatinib is at least 30 days).

- Surgical management of axillary lymph nodes includes SLNB after neoadjuvant therapy and ALND in grades I and II at the time of breast surgery. The choice of axillary surgery depends on the clinical status of the axillary, T stage and clinical practice of the research center.

## Postoperative pathological assessment

• Tumor pathological remission will be assessed through tumor tissue specimens obtained during the surgery, and all remission assessments will be assessed by each center according to the key points in Annex VI.

## Diagnosis of recurrence or recurrence

Recurrent disease includes local, regional and distant recurrence and contralateral breast cancer. For subjects with in situ breast disease or second (non-breast) malignancy, maintenance should be done through regular follow-up to keep abreast of possible subsequent recurrent disease events. If the diagnosis is in doubt (e. g., an ambiguous palpable mass in the breast radiotherapy area), histological or cytology confirmation of the recurrence should be performed as far as possible. Some subjects may have suspected recurrence and die quickly before arrival and confirming recurrence. The autopsy reports or death information for such subjects should be obtained as far as possible.

For data collection, the earliest date of diagnosis of recurrent disease should be used and recorded. The date of diagnosis should be based on clinical, radiological, histological, or cytological evidence. The date of disease recurrence should be the date of first diagnosis of the lesion (i. e. objective finding), rather than the date of symptoms.

All second primary malignancy reported during the study: a second primary malignancy diagnosed without systemic treatment (i. e., chemotherapy, hormone therapy, targeted therapy, etc.) and subjects without signs of breast cancer recurrence may continue to participate in the study, and continue to receive study drug treatment based on the study protocol and evaluation schedule, as long as the investigator considers that continued treatment is in the best interest of the subject.

The following events are not considered as recurrent disease but should be truthfully recorded:

• Ipsilateral and contralateral leaflet carcinoma in situ

• Ipsilateral and contralateral ductal carcinoma in situ

• carcinoma in situs of cervix

• Skin basal or squamous cell carcinoma

Relevant serious adverse events and non-breast second primary malignancy (as reported as serious adverse events) should also be reported until the end of the study.

## follow-up period

After completing surgical treatment, subjects should be followed up for survival indicators and adverse events.

• Safety follow-up: 30 days (±7 days) or before starting new anti-tumor treatment, safety follow-up was 90 days (±7 days) or before starting new anti-tumor treatment, the first safety visit was 30 days (±7 days) at the site. The second and third follow-ups are no longer checked for safety indicators and can be visited by telephone. All SAEs considered related to the study drug were collected after the safety follow-up period.

• Survival follow-up: All subjects will undergo survival follow-up after completing surgery / early termination of treatment. Follow-up every 3 months (±28 days) and every 6 months (±28 days), through telephone (or clinic), or their family, or local physician, or follow-up or until 5 years after the last subject is enrolled (whichever occurs first).

• Information on survival (date of death and cause of death) and after completion of study treatment (including anti-tumor therapy received) was collected and recorded for each survival visit and recorded in the corresponding table of eCRF.

## Termination of treatment visit

Subjects should come to the study site to complete the treatment termination visit (EOT) within 28 ± 7 days of the last study medication.

- - Physical examination: targeted physical examination with clinical indication.
  - weight.
  - Vital signs: pulse rate, respiratory rate, body temperature, and blood pressure.
  - ECOG grade.
  - Blood routine: red blood cell count (RBC), hemoglobin (Hb), platelet count (PLT), white blood cell count (WBC), neutrophil count (ANC), lymphocyte count.
  - Blood biochemistry: alanine aminotransferase (ALT), glutamalotranstransferase (AST), glutamytranspeptidase (γ -GT), total bilirubin (TBIL), direct bilirubin (DBIL), alkaline phosphatase (ALP), blood urea nitrogen (BUN) or urea (preferably blood urea nitrogen), total protein (TP), albumin (ALB), lactate dehydrogenase (LDH), creatinine (Cr), blood glucose (GLU), K +, Na +, Ca 2 + and Cl-.
  - Urine routine: white blood cells, red blood cells, urine protein. If the urine protein is 2 +, a 24-hour urine protein quantification test should be added.
  - Fecal occult blood test.
  - Coagulation function: including the international normalized ratio INR, activated partial thromboplastin time APTT, fibrinogen FIB, thrombin time TT; if the international normalized ratio INR is not available, then prothrombin time PT is used instead.
  - Thyroid function: serum thyroid-stimulating hormone (TSH), free triiodothyronine (FT3), free thyroxine (FT4); if FT3 and FT4 are not available, T3 and T4 are replace.
  - pregnancy tests.
  - And a 12-lead ECG.
  - echocardiogram.
  - Record of adverse events.
  - Record the concomitant medication / concomitant treatment: Record the concomitant medication / concomitant treatment until 28 days after the last dose.

## Early termination of the study visit

For disease progression (at neoadjuvant treatment) or intolerable toxicity as assessed by the investigator, subjects must return to the site for a treatment discontinuation visit (+ 7) 28 days after the last study treatment and should complete all tests required in the treatment termination visit (EOT). A complete tumor imaging session (unless done within 28 days) is recommended.

## Visit outside the plan

If the subject needs unscheduled follow-up for an adverse event, the following items should be recorded:

- - - Record the concomitant medication / concomitant therapy;
    - Record the adverse events;
    - Record relevant performed (including imaging, etc) if any.

## Biomarker examination

At least 10 white slices of formalin-fixed paraffin-embedded (FFPE) tumor tissue specimens for biomarkers such as PD-L1. Fine needle puncture specimens, brush specimens, pleural effusion cell precipitation specimens, bone metastasis specimens, and lavage specimens were not sufficient for biomarker detection and review.

Peripheral blood will be collected within 3 days before the first dose and after cycle 5, and at least 7.5 mL each time.

# appraise

## Effectiveness evaluation

### validity index

**The primary efficacy measure of the study was total pathological complete response (tpCR).**

- TpCR (ypT0/is, ypN 0): After completion of neoadjuvant therapy, no residual invasive carcinoma existed in the pathological assessment of hematoxylin and eosin-stained resected breast cancer samples and all ipsilateral lymph node samples.

**Complete breast pathological response (bpCR), residual tumor burden grade 0 and I (RCB-0 / I), objective response rate (ORR), event-free survival (EFS), and invasive disease-free survival (iDFS).**

- The bpCR (ypT0/is): After completion of neoadjuvant therapy, there was no residual invasive carcinoma in the pathological evaluation of hematoxylin and eosin-stained resected breast cancer samples.
- RCB-0 / I: After completion of neoadjuvant therapy, in the breast cancer samples and lymph node samples, the residual tumor range (mm x mm), the cell density (%), the proportion (%) of carcinoma in situ, the maximum diameter of positive lymph node (mm); input the above 5 pathological parameters into the network calculator (www.mdanderson.org/breastcancer_RCB) to obtain the RCB index and the corresponding RCB grade.
- ORR: The proportion of subjects who achieved either CR or PR as the optimal tumor response during neoadjuvant therapy. The investigator will evaluate the objective response rate according to the efficacy evaluation criteria of solid tumors (RECIST v1.1).
- EFS: Time interval between subject enrollment and the first recorded related event, including preoperative disease progression, postoperative disease recurrence, and death from any cause. Observation time was calculated for at least 5 years from randomization.
- IDFS: Time from enrollment to first episode of recurrent disease, including ipsilateral or contralateral recurrent breast cancer, local or regional recurrence, distant recurrence, and death from any cause. Observation time was calculated for at least 5 years from randomization.

**Exploratory indicators of the study**

- Potential predictive biomarker parameters related to efficacy (e. g., pCR, RCB-0 / I, EFS, iDFS, etc.) in peripheral blood and tumor specimens, including but not limited to PD-L1, CTCs, TILs, CD4 / CD8, Tregs, BRCA 1 / 2 and PI3K / AKT / mTOR.

## safety evaluation

### Safety indicators

Adverse events; serious adverse events; abnormal laboratory values; clinical presentation characteristics, severity, onset, duration, drug association, treatment and prognosis.

### adverse event

AEs are all adverse medical events occurring after the clinical trial subject received the investigational product, but are not necessarily causally related to the treatment. AEs may be any adverse and undesired symptoms, signs, laboratory abnormalities, or disease, including at least the following conditions:

1) exacerbation of original medical condition / disease (before entering clinical trials) (including exacerbation of symptoms, signs and laboratory abnormalities);

2) Any new AE: any new adverse medical condition (including symptoms, signs, newly diagnosed disease);

3) Abnormal and clinically significant laboratory test values or results

The evaluation of adverse events (AEs) included type, incidence, severity (grade according to version 5.0 NCI CTCAE), occurrence and end time, treatment measures, serious adverse events and correlation, and outcome. Adverse events from the beginning to the end of the safety follow-up period will be recorded. Only all SAEs considered related to the study drug will be collected after the safety follow-up period.

AEs occurring during the study period, including signs and symptoms during the screening period, will be recorded on the AE page of the eCRF.

### Definition of serious adverse events (SAEs)

Serious adverse events refer to the medical events that require hospitalization or prolonged hospitalization, disability, affecting work ability, life-threatening or death, and causing congenital malformation in the course of clinical research. Include the following undesirable medical events:

- Events leading to the death;
- Life-threatening event (defined as the risk of immediate death at the time of the event);
- Events requiring hospitalization or prolonged hospitalization;
- Events that can cause permanent or severe disability / dysfunction / affect work ability;
- A ital abnormality or birth defect;
- Other important medical events (although not immediately life-threatening, causing death or hospitalization, which, based on reasonable medical and scientific judgment, may cause harm to the subject or may require intervention [such as drugs or surgery] to prevent serious consequences listed in the definition above).

Adverse events leading to hospitalization or prolonged hospitalization in clinical studies should be considered SAEs. Any initial hospital admission (even if less than 24 hours) meets this criterion. Hospitalization does not include the following: rehabilitation facilities; nursing home; routine emergency room admissions; same-day surgery (such as outpatient / same-day / ambulatory surgery); social reasons (Medicare reimbursement, etc.).

Hospitalization or prolonged length of stay unrelated to worsening of AEs were not SAEs.for instance:

- He was admitted to the original disease with no new adverse events or aggravation of the original disease (e. g., to check the abnormal laboratory tests persisting before the study);
- Hospitalization for administrative reasons (e. g., annual routine physical examination);
- Hospitalization specified in the protocol during the clinical study (e. g. according per the protocol);
- Elective hospitalization not related to worsening of adverse events (e. g. elective surgery);
- Scheduled treatment or surgical procedures should be documented throughout the study protocol and / or in the baseline information of the individual subjects;
- Admitted only because of blood product use.

Diagnostic or therapeutic invasive (e. g. surgery), non-invasive procedure should not be reported as an AE, but if the disease condition causing this operation meets the definition of an AE, acute appendicitis occurring during the AE report should be reported as an AE, and the appendectomy should be recorded as the treatment for the AE.

### Adverse events, and of special interest

If the subject has adverse events of the following type and severity, report the AE of special concern (i. e. SIE, as below) specified in the study protocol, the investigator shall fill in the Adverse Event Report Form of Special Interest and notify the principal investigator and Hengrui within 24h of learning. If it is also a SAE, please complete the Serious Adverse Event Report Form simultaneously.

- - - - Grade 3 infusion reaction;
      - Grade 3 of immune-related adverse events
      - AEs that meet Section 7.2.6 criteria for abnormal liver function tests

### Immune-related adverse events

Immune-related adverse events are specific events (including pneumonia, diarrhea / colitis, hepatitis, nephritis, renal dysfunction, rash, and endocrine disease). Endocrine events (hypothyroidism / thyroiditis, hyperthyroidism, hypophysitis, diabetes, adrenal insufficiency) are usually exceptional, although irAEs, are usually unrelated to treatment and can be managed without immunosuppressive intervention.

In this study, irAEs included such events occurring within 90 days after the last dose of Camrelizumab.

### The liver function test was abnormal

If abnormal AST and / or ALT levels occur with abnormal elevation of total bilirubin levels that meet the following (1) (2) (3) conditions and no other reasons for the abnormality, such condition should be considered as an important medical event, if the SAE criteria according to the SAE procedure.

| satisfy the condition | criterion for judgement |
| --- | --- |
| (1) Abnormal of ALT or AST | Normal baseline: ALT or AST 3 ULN during treatment;  Baseline abnormalities: ALT or AST 2 baseline levels during treatment, with a value of 3 ULN, or 8 ULN. |
| (2) Abnormal TBIL | Normal baseline: TBIL> 2 ULN during the treatment period;  Baseline abnormalities: TBIL increased by 1 ULN or values> 3 ULN. |
| (3) No hemolysis, and alkaline phosphatase <2 ULN (or information not available) | |

ULN (Normal Value is Online)

If a subject has abnormal AST and / or ALT levels and an abnormal increase in total bilirubin levels during the safety follow-up period, he / she should return to the study center for evaluation as soon as possible (preferably within 48 hours) after learning the abnormal results. The evaluation should include laboratory tests, detailed medical history, and physical evaluation, and the possibility of liver tumor (primary or secondary).

In addition to repeated AST and ALT, laboratory tests should include albumin, creatine kinase, total bilirubin, direct and indirect bilirubin, γ -glutamyltransferase, prothrombin time (prothrombin time [PT]) / international standardized ratio (international normalized ratio [INR]), alkaline phosphatase, etc. Detailed medical history collection should include: history of alcohol consumption, acetaminophen, soft drugs, various supplements, family medical history, occupational exposure, sexual behavior, travel history, contact with jaundice patients, surgery, blood transfusion, liver disease or allergic disease history. Further tests may also include the detection of acute hepatitis A, B, C, and E, and liver imaging (e. g., biliary tract). If the laboratory criteria, it should be reported as an SAE in the absence of other causes of liver enzyme abnormalities.

### Severity of adverse events and interpretation criteria

Refer to the grading criteria for drug AE in NCI-CTC AE 5.0. If AEs not listed in the NCI-CTC AE 5.0 table, refer to the List 7 criteria below:

**Table 7 Reference criteria for severity of AEs not listed in NCI-CTC AE 5.0**

| **grade** | **Clinical description of the severity level** |
| --- | --- |
| 1 | Mild; asymptomatic or mild; clinical or diagnostic observation only; treatment-free. |
| 2 | Moderate; requiring small, local or non-invasive treatment; referring to cooking, clothing purchase, telephone use, financial management, etc. |
| 3 | Serious or medically significant but not immediately threatening life; hospitalization or prolonged hospitalization; disability; restricted activities of daily life. Self-rational daily living activities refer to bathing, dressing and eating  Rice, toilet, medicine, etc., are not bedridden. |
| 4 | Life-threatening; requiring urgent treatment |
| 5 | Death associated with an AE |

### Criteria for judging the relationship of the adverse events to the study drug

Adverse events include all undesired clinical manifestations, and should be reported as long as they occur after signing the informed consent form, regardless of their relationship to the study drug, whether assigned to the study drug group, or even regardless of their drug application. Any abnormal changes in patient complaints or objective laboratory indicators should be truthfully recorded, and the severity, duration of adverse events, treatment measures and outcome should be indicated.

The investigator should comprehensively determine the relationship between the adverse events and the study drug, such as whether the occurrence of AEs has a reasonable time order with the medication, the characteristics of the study drug, the toxicology and pharmacological effect of the study drug, whether the subject uses other combined drugs, the subject's underlying disease, past medical history, family history, and stimulate and restimulate reactions. The possible association between adverse events and study drug was assessed by the "positive, possible, possible, unrelated, and undetermined" classification.

### Safety assessment criteria

The assessment of adverse events (AEs) included type, incidence, severity, onset and end time, being a serious adverse event, adverse events of particular interest, treatment / drug association, and outcome.

### Laboratory safety evaluation

Laboratory test samples will be collected from the time points specified in the Clinical Trial Flow Chart. The following laboratory indicators are sampled and tested by the research center. For subject safety concerns, unplanned clinical laboratory tests may be performed at any time.

**Table 8. Laboratory examinations performed at the study site**

| routine blood test | Blood biochemical | Fecal routine | routine urine test ^a^ |
| --- | --- | --- | --- |
| hemoglobin  red blood cell  white blood cell  Neutrophil count was indicated  LC  platelet count | TBIL  DBIL  ALT  AST  ALP  γ-GT  TP  ALB  LDH  BUN  Cr  GLU  potassium  sodium  chlorine  calcium | fecal occult blood | Urinary white blood cells (WBC)  Urine protein (PRO)  Urinary red blood cells (RBC) |
| Screening of infectious diseases | coagulation function | thyroid function | other |
| Hepatitis B five  HIV antibody  HCV antibody | PT  APTT  TT  INR  FIB | Free T3  Free T4  T3  T4  TSH | pregnancy tests ^b^ |

Note: a. If the urine routine shows protein 2+, a 24-hour urine protein quantification test was performed.

b. Women of childbearing age should have blood HCG test during the screening period and at the end of treatment to exclude pregnancy, and urinary HCG test can be performed at the rest time points.

The following abnormal laboratory examination results shall be recorded:

- All laboratory findings identified as clinically significant or meeting the SAE definition;
- All abnormal laboratory results that require the subject's withdrawal or interruption of study drug;
- All laboratory findings required the subject for specific symptomatic treatment.

It is best for the reporting investigator to use clinical terms rather than laboratory terms (e. g. anemia, not low hemoglobin values).

### Pregnancy test

For fertile female subjects, serum HCG, will be performed within 72 hours before the start of dosing. Test should at least have a sensitivity of 25 mIU / mL to hCG. Appropriate contraception should begin after negative results for screening pregnancy testing. A minimum of one urine pregnancy test / blood pregnancy test is required at the termination visit. During the trial, the pregnancy test can be added according to the diagnosis and treatment routine of the trial center. Positive urinary pregnancy test during the test should be verified by blood HCG test. If the hCG test was positive, the subject was stopped and treated according to Section 8.3.

# Adverse event report

## Adverse event report

Collection of AE information should be initiated from the subject signed informed consent to the end of the safe follow-up period.

Observe and record all adverse events from signing the informed consent form per NCI CTC AE v5.0 criteria and collect them until the end of the safety follow-up period. All adverse events should be followed until the end or disappearance of the safe follow-up period, remission to baseline level or level 1 (CTCAE v5.0), steady state or reasonably explained (e. g. lost to follow-up, death).

## Report of serious adverse events

Serious adverse events should be reported when the subject signed the informed consent until the end of the safety follow-up period. In case of a serious adverse event, either the first report or the follow-up report, the investigator must immediately fill in the Serious Adverse Event (SAE) Report Form, sign and date, immediately notify the main research unit and Hengrui Drug Safety Department within 24 hours after the investigator learns of the SAE, and timely report to the relevant units according to the regulatory requirements (see Annex 5 SAE reporting method). SAEs occurring after the safety follow-up period should be collected and reported to suspected study drug participants.

Serious adverse events should be recorded in detail for symptoms, severity, correlation, time of occurrence, time of treatment, measures taken with the trial drug (Camrelizumab, apatinib, paclitaxel, cyclophosphamide, epirubicin), time and method of follow-up, and outcome, etc. If the investigator considers a serious adverse event unrelated to the trial drug and potentially related to the study condition (e. g. termination of original treatment, or comorbidities during the trial), this relationship should be detailed in the narrative section of the SAE report form. If the intensity of an ongoing serious adverse event or its relationship with the test drug changes, a follow-up report should be submitted immediately. If the investigator considers that previously reported SAEs are misreported, corrections, revocation or degradation instructions may be made in the follow-up report and reported according to the SAE reporting procedures.

## Pregnancy report

If the female subjects were pregnant during the clinical study, the subjects were enrolled. The investigator should fill in the Clinical Study Pregnancy Report / Follow-up Form within 24 hours after learning of the pregnancy event and report it to the Hengrui Drug Safety Department and timely report to the Ethics Committee.

The investigator will follow up the pregnancy results until 1 month after the mother is delivered, and report the results to the Sponsor and the Hengrui Drug Safety Department. If the pregnancy result is stillbirth, spontaneous abortion, and fetal malformation, it is considered an SAE and needs to be reported within the time limit of the SAE.

If a subject has an SAE during pregnancy, complete the Serious Adverse Event Report Form and report with SAE reporting time and procedures.

SAE / Pregnancy Report Email address: hengrui_drug_safety@hrglobe.cn.

## Disease progression and death reports

Disease progression was defined as the deterioration of the subject's condition due to the indication of the study drug. Including radiographic evidence of progression, or the progression of clinical symptoms and signs. The new metastasis of the primary tumor, or the progression of the original metastasis were considered to be disease progression. Life-threatening events resulting from symptoms and signs of disease progression, requiring hospitalization or prolonged hospitalization, or causing permanent or severe disability/ dysfunction/affecting work capacity, congenital abnormalities or birth defects, are not reported as SAEs. If there is any uncertainty about whether the SAE is due to disease progression, it should be reported as an SAE.

In the study population of this trial, "disease progression" is expected to occur and should not be reported as an AE term. When disease progression occurs, the event used to confirm disease progression should be reported as an AE. For example, the subject developed epilepsy and was determined to be related to brain metastasis, and the AE term should be recorded as "epilepsy" rather than "disease progression" or "brain metastasis".

If the subject dies during the trial, whether he or she has received new antitumor therapy, it must be reported as a SAE (see Table 8 AE / SAE collection and follow-up duration principles).

Death as assessed by the investigator due to signs and symptoms of disease progression may be reported as an SAE. However, the word "death" should not be used as an AE or a SAE term, but as the result of an event. The event causing or leading to death should be recorded as an SAE. If the cause of death is unknown and could not be determined at the time of reporting, the SAE term is recorded as "unexplained death".

## Drug overdose report

An overdose is an accidental or intentionally excessive and medically significant dose, with the dosage of either study drug exceeding 120% of the dose specified in the regimen. A single overdose event does not need to be reported per an SAE. If an AE / SAE occurs due to an overdose, the AE / SAE should be recorded separately, otherwise only protocol violations were recorded.

## Collection and follow-up of the AE / SAE

Collection of AE / SAE should start when the subject signed the informed consent until the end of the safety follow-up period.

All AEs / SAEs should be followed until the end or disappearance of the safe follow-up period, remission to baseline or level 1, stable status, or reasonably explained (e. g. lost to follow-up, death).

The investigator should inquire about the AE / SAEs occurring after the last visit at each visit and provide timely follow-up information according to the challenge requests received.

For details, please refer to the following table:

**Table 9 Principles of collection and follow-up duration for AE / SAE**

| **classify** | **Collect requirements** | **Follow-up requirements** |
| --- | --- | --- |
| AE / SAE, with or without a drug association | By the end of the safety follow-up period | Follow-up to disappearance, remission, or to baseline, or grade 1, or stable status, or reasonably explained (e. g. lost to follow-up, death). |
| SAE with a drug relevance | indefinite duration | Follow-up to disappearance, remission, or to baseline, or grade 1, or stable status, or reasonably explained (e. g. lost to follow-up, death). |

Note: 1. Safety follow-up period refers to subjects with Camrelizumab from 30 days (±7 days) after the last medication or before the start of new anti-tumor treatment, safety follow-up is 90 days (±7 days) after the last dose or before the start of new anti-tumor therapy, whichever comes first. Only all SAEs considered related to the study drug will be collected after the safety follow-up period.

2. If the death occurs during the safety follow-up period, it must be reported as an SAE.

# Data analysis and statistical methods

## sample capacity

This study is a single arm design, and according to the study data of the KEYNOTE-522 and IMpassion031 trial treatment regimen, the tpCR rate of Camrelizumab+ apatinib + chemotherapy regimen in early TNBC. Using NCSS PASS 15 (LLC. Kaysville,Utah,USA,ncss.com/software/pass), the 95% confidence interval of tpCR rate for the approximately 31 subjects was [44%, 78%]. Considering the 10% shedding rate, a total of 35 subjects will be enrolled in this study.

## Statistical analysis plan

Detailed summary and statistical analysis methods for the data collected in the study will be included in the Statistical Analysis Plan (SAP), finalized and filed by the sponsor. Any changes to the study protocol, as determined by the sponsor or the principal investigator, require the SAP to align with the study protocol. The statistical analysis plan may be revised to the relevant content of this protocol, but if the main and / or key factors of the protocol are defined as endpoints or its analysis, the revision should also be reflected in the protocol amendment.

## Analyze the crowd

The analysis population of this study included the full analysis set (Full Analysis Set, FAS), the per-protocol set (Per Protocol Set, PPS), and the safety set (Safety Set, SS).

Full Analysis set (FAS set): included all subjects enrolled and received at least one study medication. The FAS set was the primary analysis set for the efficacy endpoints in this study.

Protocol Compliance Analysis Set (PPS Set): A subset of the FAS set, including subjects meeting the FAS set definition, all subjects who completed protocol specified treatment and visits and had no major protocol deviations. The judgment criteria for major protocol deviations should be finalized before the database lock library, and the list of subjects included or excluded from the PPS set should be determined by the principal investigator and statistician in the data review meeting before the database lock library. The PPS set is the secondary analysis set for the efficacy endpoints of this study.

Safety Analysis set (SS set): included all subjects enrolled and received at least one study medication. The SS sets will be used for all safety analyses.

## statistical method

### General principles for statistical analysis

Analysis will be performed using SAS ® System version 9.4 or above, and all tables, pictures and lists will be generated in an RTF file.

For descriptive statistical analysis, unless specified, number of subjects (n) versus percentage (%) and missing versus percentage for subtype data will be provided. For continuous data, the number of non-missing subjects (n), arithmetic mean, standard deviation, median, minimum, maximum will be provided.

### efficiency analysis

The analysis of the efficacy endpoints of this study will be based on the FAS set and PPS set and will dominate the results of the FAS set.

The primary analysis will be conducted for the primary efficacy measure, the investigator-assessed tpCR. The secondary analysis will be conducted for the secondary efficacy measures, which refers to bpCR, RCB-0 / I, ORR, EFS, and iDFS.

For the efficacy endpoint of the second classification (including pCR, RCB-0 / I, ORR), the number and percentage of subjects under each classification will be summarized and the 95% confidence interval calculated using the Clopper-Pearson method.

For efficacy endpoints (including EFS, iDFS), the number and percentage of subjects with events and censoring were summarized and summary statistics were pooled for event types and cause of censoring.

The 3-and 5-year EFS, iDFS rates will be estimated using the Kaplan-Meier method and survival plots will be drawn. The 95% confidence interval for survival was calculated using the log (-log) method.

### safety analysis

All safety analyses will be based on the SS set. All adverse events will be graded according to NCI-CTCAE 5.0, with descriptive statistics: AE, SAE, Grade 3 AE, Grade 3 SAE, drug-related AE, AE, dose adjustment, and AE, treatment termination. For AEs of special concern, frequency and percentage were summarized, including the mean, standard deviation, median, maximum and minimum.

Laboratory test results, vital signs, and electrocardiogram data will be analyzed from baseline and after baseline.

### exploratory analysis

Correlation analysis of pCR, ORR, RCB-0 / I, EFS and iDFS according to the biomarker condition.

# Research management

## Ethics practices and informed consent

### Ethical norms

This clinical trial must comply with the Declaration of Helsinki (2008 edition), the Good Laboratory Practice (GCP) of the NMPA (formerly CFDA) and relevant regulations. Before the study, approval from the Ethics committee must be obtained. During the clinical study, some modifications of the trial protocol should be reported to the Ethics committee and filed. The investigator has the responsibility to submit the mid-term report regularly according to the relevant requirements of the EC, and shall notify the EC that the trial has ended.

### informed consent

The subject must give informed consent to participate in the trial before receiving the drug to protect the legitimate rights and interests of the subject. The investigator has the responsibility to fully and comprehensively inform the subject or his designated representative about the purpose of the study, the role of the drug, possible toxic effects and possible risks, informing the subject of their rights, risks and benefits. Talking is an extremely important informed consent process. If the subject and his legal representative are not literate, the informed consent process shall be attended by the witness. After the oral consent of the subject or the legal representative signs the informed consent form, and the signature of the witness shall be on the same day as that of the signature of the subject. The informed consent form shall indicate the version number and version date.

## Test drug management

The management, distribution and recovery of the clinical drugs in this trial shall be the responsibility of special personnel. The investigator must ensure that all experimental drugs are only used for subjects participating in the clinical trial, their dosage and usage, and the remaining drugs shall be returned to the research center and the clinical drugs shall not be transferred to any non-clinical trial participants.

The monitor is responsible for monitoring the supply, use, storage of clinical trial drugs and the processing process of surplus drugs.

## Revision of the programme

No one can modify the protocol except the study leader. Any necessary changes to the protocol shall be made in the form of protocol revision, and shall be submitted to the Ethics Committee for approval or filing after signing and agreeing by the investigator, and the details of previous modifications shall be explained in the protocol.

## inspect

The research sponsor (Hengrui Medicine) shall appoint personnel with appropriate medical, pharmacy or related education, familiar with GCP and relevant regulations as necessary to monitor the clinical trial to monitor and report the progress and verify the data, ensure that the trial records and the reported data are accurate, complete and in compliance with the approved protocol, GCP and relevant regulations.

Monitor the adverse events and serious adverse events in clinical trials to ensure that all adverse events are accurate, reliable and quickly recorded and reported.

## Quality control and assurance

The clinical research unit must be a drug clinical research base as determined by NMPA (formerly CFDA);

Researchers must be physicians trained in clinical trials and work under the guidance of senior professionals;

Before the test, the clinical ward must meet the standardized requirements and ensure that the rescue equipment is complete;

Professional personnel shall guide the subjects to take drugs, understand the use of drugs in detail, and ensure the compliance of the subjects;

Each sites must strictly follow the protocol and fill in the eCRF truthfully;

The monitor shall follow the standard operating procedures, supervise the clinical trial, confirm the records and reports of all data, all eCRFs are filled correctly and consistent with the original data, and ensure that the trial is performed in accordance with the clinical study protocol;

Once the SAE occurs, the monitor shall notify each test unit in time and temporarily stop the study if necessary;

All centers participating in the trial should be audited by the authorities, and it is particularly important that the investigator and their related personnel should provide convenience and time for monitoring and audit.

## data management

### Collection of data

This study uses the eCRF for study data collection; the monitor will train the designated research institution personnel in the EDC system. Institution staff can only be trained to log on to the EDC system. The investigator or dedicated data entry personnel (CRC) shall enter the data into the EDC system in accordance with the requirements of the visit process and the eCRF filling guidelines. The system logic verification procedure will check the integrity and logic of the clinical trial data entered into the EDC system and generate error information prompts for the problem data to allow the modification or interpretation of the problem data by the investigator or CRC. After the database is locked, the investigator will receive a copy of the CD-ROM or document of the patient data for archiving at the study facility.

### Data management and quality control

In order to ensure the authentic and reliable clinical trial data and improve the quality of clinical data, the clinical monitor during the trial project will review the trial data for integrity, consistency and accuracy of the clinical database according to the standard operation process, and guide the research institution personnel to make necessary supplements or corrections to the problem data. The clinical monitor or data management personnel will question the investigator or the CRC in the form of an electronic challenge form. The investigator or the CRC must respond to the question and correct or explain the problem data, and may issue questions several times if necessary until the problem data is resolved. At the end of the trial project, the data management personnel and medical personnel will conduct the final quality control of all the data in the database, summarize all the events of protocol deviation and protocol violation during the trial, and hold a data verification meeting. After the data in the database meets the quality requirements, the locked data manager of the database will export the data to the statistical department for data analysis.

### Review of data and monitoring of research institutions

Prior to the trial, the monitor introduced the trial protocol and the eCRF during the initial visit to the study facility or the investigator. During the trial, the monitor will visit the site regularly to check the completeness of the patient records and the accuracy of the eCRF, compliance with the protocol and clinical trial quality practices, and ensure that the test drugs are stored, distributed and counted as specified. During these visits, the key researchers must be able to assist the monitors. The investigator must maintain the original documentation of each patient enrolled in the trial, including study medical records and visit records (inpatient or outpatient medical records), including demographic indicators and medical information, laboratory data, ECG, and results of any other examinations or evaluations. All information on the eCRF must be derived from the original file in the patient file. The investigator must also keep the informed consent form signed by the patient.

The investigator must confirm that all relevant original documents can be monitored to verify that they are consistent with the eCRF content. Monitoring standards require that 100% monitoring has obtained informed consent, compliance with inclusion / exclusion criteria, records of SAEs, and data required for evaluation of all major and safety indicators. Additional checks of the consistency of the raw data and the eCRF were performed following the monitoring plan specified for the trial. Any information on the patient&#039;s identity in the original documents will not be made publicly available.

## The scheme against

All the requirements specified in the study protocol must be strictly enforced. Any intentional or unintentional deviation or violation of the trial protocol and GCP principles may be classified as a deviation or violation of the protocol. In the process of monitoring, either the investigator or the monitor shall fill in the violation record, record the time found, time, process of the event and the corresponding treatment measures, signed by the investigator and notified to the Ethics committee.

## Data preservation

In order to ensure the evaluation and supervision of clinical research by the State Food and Drug Administration, the investigator shall agree to keep all the study data, including the original records of the subject&#039;s hospitalization, informed consent, case report form, detailed records of drug distribution, etc. The study data shall be kept by the research institution until 5 years after the end of the clinical trial. All the data of this clinical study belongs to the applicant, and, unless required by CFDA, shall not be provided to any third party in any form without the written consent of the applicant.

## Publication of the study results

All articles and reports related to the trial should be published only with the consent of the investigator and the applicant.

# Case off

All subjects who have completed informed consent and screened for entry shall have the right to withdraw from the clinical trial at any time. Regardless of withdrawal, subjects who have not completed at least 1 trial drug dose and cannot safety and efficacy evaluation were shedding cases (those with disease progression and clear medical evidence). When the subject falls off, the investigator must fill out the shedding reason in the eCRF, complete the possible assessment items, and carefully complete the last visit record in the eCRF. Those shedding due to adverse reactions and finally judged to be associated with the trial drug after follow-up should be recorded in the eCRF and notified to the investigator. Subjects who withdrew from the study after screening without a drug number were not included as shedding cases. If the subject completed a complete cycle with detailed records, statistical analysis should be performed during safety evaluation.

Subjects withdrawn from the study must not re-enter the study and their numbers cannot be used again.

# Research progress

(1) April 2021 to December 2021: Study start-up stage, including protocol design, EDC database construction, trial data preparation, investigator screening, ethical approval, center agreement signing, participant research personnel training, project launch meeting and other study preparation;

(2) December 2021 to December 2022: First subject enrollment expected December 2021, last subject enrollment September 2022, and last patient end treatment follow-up (surgery) March 2023.

(3) April 2023 to June 2023: In the data analysis stage, collect data, make statistical analysis, write the paper and submit it, conclude the topic and complete the result appraisal.

(4) July 2023 to June 2028: Five-year survival follow-up, collect survival data, statistical analysis, write the paper and publication.

# reference documentation

- - - 1. Pareja F, Reis-Filho JS: Triple-negative breast cancers - a panoply of cancer types. Nat Rev Clin Oncol. 2018;15(6):347–8. 10.1038/s41571-018-0001-7.
      2. 2020 NCCN Guidelines Breast Cancer Version 6.2020 — Sep 8, 2020.
      3. Breast Cancer Professional Committee of Chinese Anti-Cancer Association, 2019 Guidelines and Guidelines of Chinese Anti-Cancer Association. [J] Chinese Journal of Cancer, 2019,29 (8): 609-680.
      4. von Minckwitz G, Schneeweiss A, Loibl S, et al. Neoadjuvant carboplatin in patients with triple-negative and HER2-positive early breast cancer (GeparSixto; GBG 66): a randomised phase 2 trial. Lancet Oncol . 2014;15:747-756.
      5. Sikov WM, Berry DA, Perou CM, et al. Impact of the addition of carboplatin and/or bevacizumab to neoadjuvant once-per-week paclitaxel followed by dose-dense doxorubicin and cyclophosphamide on pathologic complete response rates in stage II to III triple-negative breast cancer: CALGB 40603 (Alliance). J Clin Oncol . 2015;33:13-21.
      6. Nanda R, Chow LQ, Dees EC et al. Pembrolizumab in Patients With Advanced Triple-Negative Breast Cancer: Phase Ib KEYNOTE-012 Study. J Clin Oncol 2016; 34: 2460-2467.
      7. Hu, Xichun, et al. "Multicenter phase II study of apatinib, a novel VEGFR inhibitor in heavily pretreated patients with metastatic triple‐negative breast cancer." International journal of cancer 135.8 (2014): 1961-1969.
      8. G, Henschel V, Molinero L, Chui SY, et al. Atezolizumab and Nab-Paclitaxel in Advanced Triple-Negative Breast Cancer. N Engl J Med. 2018 Nov 29;379(22):2108-2121.
      9. Schmid P, Cortes J, Pusztai L, et al. Pembrolizumab for Early Triple-Negative Breast Cancer. N Engl J Med. 2020 Feb 27;382(9):810-821.
      10. Mittendorf EA, Zhang H, Barrios CH, et al. Neoadjuvant atezolizumab in combination with sequential nab-paclitaxel and anthracycline-based chemotherapy versus placebo and chemotherapy in patients with early-stage triple-negative breast cancer (IMpassion031): a randomised, double-blind, phase 3 trial. Lancet. 2020 Oct 10;396(10257):1090-1100.
      11. Schmittnaegel, Martina, et al. Dual angiopoietin-2 and VEGFA inhibition elicits antitumor immunity that is enhanced by PD-1 checkpoint blockade. Science translational medicine 9.385 (2017): eaak9670.
      12. Allen E , Jabouille A , Rivera LB , et al . Combined antiangiogenic and anti-PD-L1 therapy stimulates tumor immunity through HEV formation. Sci Transl Med 2017; 9: eaak9679.
      13. Li Q , Wang Y , Jia W , et al . Low-dose anti-angiogenic therapy sensitizes breast cancer to PD-1 blockade. Clin Cancer Res 2020;26: 1712–24.
      14. Xu J, Zhang Y, Jia R, et al . Anti-PD-1 Antibody SHR-1210 Combined with Apatinib for Advanced Hepatocellular Carcinoma, Gastric, or Esophagogastric Junction Cancer: An Open-label, Dose Escalation and Expansion Study. Clin Cancer Res. 2019 Jan 15;25(2):515-523.
      15. Mei K, Qin S, Chen Z, et al. Camrelizumab in combination with apatinib in second-line or above therapy for advanced primary liver cancer: cohort A report in a multicenter phase Ib/II trial. J Immunother Cancer. 2021 Mar;9(3):e002191.
      16. Liu JQ , Liu Q, Li Y, et al . Efficacy and safety of camrelizumab combined with apatinib in advanced triple-negative breast cancer: an open-label phase II trial. J Immunother Cancer. 2020 May;8(1):e000696.
      17. Bear HD, Tang G, Rastogi P, et al. Bevacizumab added to neoadjuvant chemotherapy for breast cancer. N Engl J Med. 2012; 366:310–20.
      18. von Minckwitz G, Loibl S, Untch M, Eidtmann H, Rezai M, Fasching PA, et al. Survival after neoadjuvant chemotherapy with or without bevacizumab or everolimus for HER2-negative primary breast cancer (GBG 44-GeparQuinto). Ann Oncol 2014;25:2363–72.
      19. Cortes J, Cescon DW, Rugo HS, et al. Pembrolizumab plus chemotherapy versus placebo plus chemotherapy for previously untreated locally recurrent inoperable or metastatic triple-negative breast cancer (KEYNOTE-355): a randomised, placebo-controlled, double-blind, phase 3 clinical trial. Lancet. 2020 Dec 5; 396(10265):1817-1828.
      20. Socinski MA, Jotte RM, Cappuzzo F, et al. Atezolizumab for First-Line Treatment of Metastatic Nonsquamous NSCLC. N Engl J Med. 2018 Jun 14; 378(24):2288-2301.
      21. Zhi-Ming Shao. 2021 ASCO Abstract 1007.
      22. J.B.A.G.Haanen, et al. Management of toxicities from immunotherapy: ESMO Clinical practice Guidelines for diagnoses, treatment and follow-up. Annals of Oncology 28 (Supplement 4): iv119-iv142,2017.
      23. Working Committee of the Chinese Society of Clinical Oncology, Guidelines for the Management of Immune checkpoint inhibitors 2021. People's Health Publishing House, 2021.4.

**Attachment I Clinical Stage Criteria for Breast Cancer (AJCC Breast Cancer TNM Stage)**

| Phase 0 | TisN0M0 |
| --- | --- |
| Phase I | T1N0M0 |
| Phase IIA | T0N1M0 |
|  | T1N1M0 |
|  | T2N0M0 |
| Phase IIB | T2N1M0 |
|  | T3N0M0 |
| Phase IIIA | T0N2M0 |
|  | T1N2M0 |
|  | T2N2M0 |
|  | T3N1,2M0 |
| Phase IIIB | T4N0M0,T3N1M0,T4N2M0 |
| Phase IIIC | Any T, N3M0 |
| Phase IV | Any T of any N, M1 |

**Attachment II Physical Status Scoring Criteria (ECOG)**

(Eastern Cooperative Oncology Group)

| Activity score | description |
| --- | --- |
| 0 | Asymptomatic, fully active, and able to perform unrestricted activities. |
| 1 | Symptoms, can walk, but heavy physical activity is limited, can engage in light Or to sit-based, work, such as minor housework, office work. |
| 2 | Symptoms, can walk, life can take care of themselves, but can not have any physical strength |
| 3 | Symptoms, limited ability to take care of themselves, awake time Chair> 50%, but not yet bedridden |
| 4 | Completely lost function, living completely unable to take care of themselves, bedridden. |
| 5 | die. |

**Annex III efficacy criteria for solid tumors**

Efficacy Evaluation Criteria for Solid Tumors Version 1.1 (Excerpt)

（ New Response Evaluation Criteria in Solid Tumors：Revised RECIST Version 1.1）

Description: This attachment is an internal translation material for reference only. Please refer to the English version.

1. background

summary

1. purpose

summary

1. Measureability of the tumors at the baseline levels
   1. definition

At baseline, tumor lesions / nodes will be measurable and not measurable as defined below:

- - 1. Measured lesions

Tumor lesion: at least one diameter (which can be recorded as maximum) with the following minimum length:

- CT scan 10 mm (CT scan layer thickness not greater than 5mm)
- Clinical routine examination instrument 10 mm (tumor lesion cannot be accurately measured with diameter instrument should be recorded as unmeasurable)
- Chest X-ray at 20 mm
- Malignant lymph nodes: Pathologically enlarged and measurable, short CT scan diameter 15 mm (CT scan thickness recommended not more than 5 mm). At baseline and follow-up, only short paths were measured and followed up.
  - 1. Non-measurable lesions

All other lesions, including small lesions (maximum diameter <10 mm or 10 mm to <15 mm) and unmeasurable lesions. Unmeasured lesions include meningeal disease, ascites, pleural or pericardial effusion, inflammatory breast cancer, cancerous lymphangitis of the skin / lung, imaging of unconfirmed and follow-up abdominal mass, and cystic lesions.

- - 1. Specific considerations regarding the lesion measurement

Bone lesions, cystic lesions, and previously locally treated lesions should be specifically noted:

Bone lesions:

- Bone scan, PET scan or plain film are not suitable for measuring bone lesions, but can be used to confirm the presence or disappearance of bone lesions;
- If an osteolytic lesion or a mixed osteogenic lesion has a defined soft tissue component that meets the above measurable definition, these lesions can be considered as measurable lesions if they can be evaluated by tomographic imaging techniques such as CT or MRI;
- Osteogenic lesions are non-measurable lesions.

Cystic lesions:

- Lesions that meet the criteria for the definition of radiographic simple cyst should not be considered malignant lesions because they are a simple cyst by definition, neither measurable lesions nor unmeasurable lesions;
- If it is a cystic metastatic lesion and meets the above measurable definition, it may be a measurable lesion. However, if non-cystic lesions exist in the same patient, non-cystic lesions should be preferred as the target lesion.

Topically treated lesions:

- Lesions located at sites previously irradiated or treated with other regional areas are generally considered as non-measurable lesions, unless the lesion has clearly progressed. The study protocol should describe in detail the conditions under which these lesions are measurable lesions.
  1. Description of measurement method
     1. Focal measurement

For clinical evaluation, all tumor measurements were recorded in the metric rice system. All baseline assessments of tumor lesion size should be completed as close as possible prior to treatment initiation and must be done within 28 days (4 weeks) before treatment initiation.

- - 1. evaluation methodology

The same technique and methods should be used for the baseline assessment and subsequent measurements of the lesions. All lesions must be evaluated using imaging, except those that cannot be examined and can only be evaluated by clinical examination.

Clinical lesions: Clinical lesions can only be considered as measurable lesions (such as skin nodules) when they are superficial and measured at 10 mm in diameter. For patients with skin lesions, color photographs containing a scale to measure the size of the lesion, are recommended for archiving. When the lesions are evaluated using both imaging and clinical examination, imaging evaluation should be used as far as possible because the imaging is more objective and reproducible at the end of the study.

Chest X X: When tumor progression is an important study endpoint, chest CT should be preferred because CT is more sensitive than X-ray, especially for new lesions. Chest X-ray detection is applicable only if the measured lesion is well defined and the lungs are well ventilated.

CT, MRI: CT is currently the best available and reproducible method for efficacy evaluation. The definition of measurability is based on the 5 mm thickness of the CT scan layer. If the CT layer thickness is greater than 5 mm, the minimum measurable lesion should be 2 times the layer thickness. MRI is also acceptable in some cases (e. g. whole body scan).

Ultrasound: Ultrasound should not be used as a measurement method to measure the lesion size. Because of its operation dependence, ultrasound examination is not reproducible after the end of measurement, which cannot guarantee the identity of technology and measurement between different measurements. If new lesions are identified using ultrasound during testing, they should be confirmed using CT or MRI. If radiation exposure to CT is considered, MRI can be used instead.

Endoscopy, laparoscopy: These techniques are not recommended for objective tumor evaluation, but they can be used to confirm CR in biopsy specimens obtained or in trials of recurrence after the study endpoint of CR or surgical resection.

Tumor markers: Tumor markers cannot be used alone to evaluate the objective tumor response. However, if the marker level exceeds the upper normal limit at baseline, it must return to normal when used to evaluate complete remission. Because tumor markers vary by disease, this factor needs to be taken into account when writing the measurement criteria into the protocol. Specific criteria for CA-125 remission (recurrent ovarian cancer) and PSA (recurrent prostate cancer) remission have been published. In addition, the International Gynecological Cancer Organization has formulated the CA-125 progression criteria, which will be added to the tumor objective evaluation criteria for the first-line treatment of ovarian cancer.

Cytology / histology techniques: in the specific circumstances specified in the protocol, these techniques can be used to identify PR and CR (such as residual benign tumor tissue often present in lesions of germ cell tumors). When exudation may be a potential side reaction of a therapy (e. g., treatment with taxane compounds or angiogenesis inhibitors) and measurable tumor meets the criteria for remission or disease stabilization, tumor-related exudation occurs or aggravation during treatment can be confirmed by cytology to distinguish between remission (or disease stabilization) and disease progression.

1. Evaluation of tumor remission
   1. Assessment of all tumors and measurable lesions

To evaluate the objective response or possible future progression, a baseline assessment of the total tumor burden in all tumor lesions is necessary for the future measurements. In a clinical regimen with objective remission as the primary treatment endpoint, only patients with measurable lesions at baseline will be enrolled. A measurable lesion was defined as the presence of at least one measurable lesion. For trials with disease progression (time to progression or degree of fixed date progression) as the primary treatment endpoint, the protocol inclusion criteria must be limited to patients with measurable lesions or no measurable lesions can be included.

- 1. Baseline recordings of both target and non-target lesions

When there are more than one measurable lesions during the baseline assessment, all lesions should be recorded and measured, a total of not more than 5 (no more than 2 each organ), as the target lesions represent all involved organs (that is, patients with only one or two cumulative organs choose up to two or four target lesions as the baseline measurement lesions).

Target lesions must be selected based on size (longest diameter), representative of all organs involved, and measurements must be well reproducible. Sometimes when the largest lesion cannot be repeatedly measured, the largest lesion can be reselected.

Lymph nodes need special attention because they are normal tissue and can be detected by imaging even in the absence of tumor metastasis. Pathological lymph nodes defined as measurable nodules or even target lesions must meet the following criteria: CT measurement of a short diameter of 15 mm. Baseline only detects the short diameter. Radiologists usually use the short diameter of the nodule to determine whether the nodule has a metastatic tumor. The nodular size is generally expressed by two-dimensional data from imaging detection (CT axial plane and MRI selects a plane from the axial, sagittal or coronal plane). The minimum value is the short diameter. For example, a 20 mm 30 mm abdominal nodule with a short diameter of 20 mm can be regarded as a malignant, measurable nodule. In this example, 20 mm is the measurement of the nodule. Nodules with 10 mm diameter but <15 mm should not be considered as target lesions. However, nodules <10 mm do not belong to the category of pathological nodules and do not need to be recorded and further observed.

The sum of the calculated diameter (including the longest diameter of non-nodular lesions and the short diameter of nodular lesions) will be reported as the sum of the baseline diameters. If containing the lymph node diameter, as mentioned above, only the short diameter is included. The sum of the baseline diameters will serve as the reference value for the disease baseline level.

All remaining lesions including pathological lymph nodes may be considered non-target lesions, but should be recorded at baseline assessment. If recorded as "present", "missing" or in rare cases "clear progress". Widespread target lesions can be recorded with target organs (e. g., massive expanded pelvic lymph nodes or large liver metastases).

- 1. Relieve the standard
     1. Target lesion assessment

Complete response (CR): All target lesions disappear, and the short diameter of all pathological lymph nodes (including target nodules and non-target nodules) must be reduced to <10 mm.

Partial response (PR): The sum of target lesion diameter decreased by at least 30% from baseline.

Disease progression (PD): Using the minimum of the sum of all target lesions measured throughout the experimental study, the diameter and relative increase are at least 20% (baseline if the minimum baseline measurement); otherwise, the absolute increase of at least 5 mm must be met (one or more new lesions are also considered as disease progression).

Disease stability (SD): the degree of target lesion reduction did not reach PR, the degree of increase did not reach the level of disease progression, between the two, the minimum value of the sum of the diameter can be used as a reference.

- - 1. Precautions for target lesion evaluation

Lymph node: Even if the lymph nodes identified to be the target lesion decrease to less than 10 mm, the actual short diameter value corresponding to the baseline should be recorded at each measurement (consistent with the anatomical plane at the baseline measurement). This means that if the lymph node belongs to the target lesion, even if the criteria for complete remission are achieved, it cannot be said that the lesion has all disappeared, because the short diameter of the normal lymph node is defined as <10 mm. The target lymph short lesion needs to be recorded at specific locations in the CRF table or other recording methods: for CR, all lymph short diameters must be <10 mm; for PR, SD, and PD, the actual measurements of the target lymph short diameter will be included in the sum of the target lesion diameter.

Small to unmeasurable target lesions: In clinical studies, all baseline recorded lesions (nodules or non-nodules) should be recorded again in the later evaluation, even if the lesions are very small (e. g. 2 mm). But sometimes it may be too small to cause the CT scan to be blurred, and the radiologist is struggle to define the exact value, potentially it as &quot;too small to measure.&quot;In this case, it is important to record a value on the CRF table. If the radiologist believes that the lesion may have disappeared, it should also be recorded as 0 mm. If the lesion is indeed present but is vague and a precise measurement cannot be given, the default can be 5 mm.(Note: lymph nodes are unlikely to do this because they normally have a measurable size or are often enclosed by adipose tissue as in the retroperitoneal cavity; but if this measurement cannot be given, the default is 5 mm). The default value of 5 mm stems from the cut thickness of the CT scan (this value is not changed by the different cut thickness values of the CT scan). Since the same measurement is not repeated, providing this default value will reduce the risk of misassessment. Repeat, however, that if the radiologist can give the exact value of the lesion size, the actual value must be recorded even if the lesion is less than 5 mm in diameter.

Isolated or combined lesion: When the non-nodular lesion is divided into fragments, the longest diameter of each separated part is combined to calculate the sum of the lesion diameter. Similarly, for the binding lesions, they can be distinguished by the plane between the bound parts, and then the respective maximum diameter is calculated. However, if the combination is inseparable, the longest diameter should be taken as the longest diameter of the whole fusion lesion.

- - 1. Assessment of non-target lesions

This section defines the remission criteria for non-target lesion tumors. Although some non-target lesions are actually measurable, no measurement requires only a qualitative assessment at the time points specified in the protocol.

Complete response (CR): All non-target lesions disappeared and tumor markers returned to normal levels. All lymph nodes were of non-pathological dimensions (short diameter <10 mm).

Non-complete response / non-disease progression: presence of one or more non-target lesions and / or persistence of tumor markers at levels greater than normal.

Disease progression: a definite progression of preexisting non-target lesions. Note: The presence of one or more new lesions is also considered as disease progression.

- - 1. Special considerations for the progression assessment of non-target lesions

The supplementary explanation of the definition of non-target lesion progression is as follows: When a patient has a measurable non-target focus, even if the target lesion is assessed as stable or partial remission, to make a clear definition of progression based on the non-target focus, the overall deterioration of the non-target lesion has reached the point that the treatment must be terminated. However, the general increase in the size of one or more non-target lesions is often not enough to meet the progression criteria. Therefore, the overall tumor progression is almost rare in the target lesion when the target lesion is stable or partially relieved.

This occurs when patients&#039; non-target lesions are not measurable: in some phase III trials, when measurable lesions are not specified in the inclusion criteria. The overall assessment was based on the above criteria, but because there was no measurable data for the lesion in this case. The deterioration of the target lesions is not easy to assess (according to the definition: must all non-target lesions can indeed be measured), so when the target lesions change increases the overall disease burden of the target lesions of the disease progression, according to the definition of the target focus to make clear progress, need to establish an effective detection method for evaluation. As described, an increase in tumor burden is equivalent to an additional 73% increase in volume (equivalent to a 20% increase in measurable lesion diameter). Or peritoneal exudation from &quot;trace&quot; to &quot;massive&quot; lymphangiopathy from &quot;local&quot; to &quot;widespread spread&quot;; or described in the protocol as &quot;sufficient to change treatment&quot;. Examples include pleural exudate from trace to mass, spread of lymphatic involvement from the primary site to the distance, or may be described as &quot;necessary therapeutic changes&quot; in the protocol. If definite progression is found, the patient should be treated as disease progression at that time point. It is best to have objective criteria that can be applied to the assessment of unmeasurable lesions, noting that the increased criteria must be reliable.

- - 1. New lesions

The presence of new malignant lesions indicates the progression of the disease; so some evaluation of the new lesions is very important. There are no specific criteria for imaging detection of lesions, however the discovery of a new lesion should be clear. For example, progression cannot be attributed to differences in imaging techniques, changes in imaging morphology, or other lesions other than the tumor (for example, some so-called new bone lesions are only the cure of the original lesion, or the recurrence of the original lesion). This is important when a patient&#039;s baseline lesion is partially or completely reactive, for example, a necrosis of a liver lesion may be identified as a new cystic lesion on the CT report, but not.

Lesions detected at follow-up but not detected at baseline will be considered as new and estive of disease progression. For example, if a patient with a visceral lesion on baseline examination has metastases on CT or MRI, his intracranial metastases will be considered as the basis for disease progression, even if he did not have a cranial examination at baseline examination.

If a new lesion is unclear, for as due to its small morphology, further treatment and follow-up evaluation are needed to confirm whether it is a new lesion. If repeated examinations confirms it is a new lesion, the time of disease progression should be counted from the time of its initial discovery.

FDG-PET assessment of lesions generally requires additional testing for complementary confirmation, and the combination of FDG-PET and supplementary CT test results is reasonable to evaluate progression (especially for new suspected diseases). New lesions may be identified by FDG-PET according to the following procedures:

Baseline FDG-PET was negative and the following FDG-PET was positive, indicating progression of the disease.

No baseline FDG-PET test was performed, and the subsequent FDG-PET test result was positive:

If the follow-up FDG-PET positive examination result was consistent with the CT examination results, the disease progression was proved.

If the new lesion found by the positive test result of the follow-up FDG-PET is not confirmed by the CT test result, the CT test should be confirmed again (if confirmed, the time of disease progression starts from the previous FDG-PET abnormality).

If the follow-up FDG-PET results with a pre-existing lesion by CT and the lesion does not progressive on imaging tests, no disease progression.

- 1. Best overall efficacy evaluation

The best overall efficacy evaluation is the best efficacy record from the beginning of the trial to the end of the trial, with any necessary conditions taken into consideration for confirmation. Sometimes the efficacy response occurs after the end of treatment, so the regimen should specify whether the efficacy evaluation after the end of treatment should be considered in the best overall efficacy evaluation. The protocol must clarify how any new treatment affects optimal efficacy response. The best response response depends on the outcome of the target and non-target lesions and the performance of the new lesions. In addition, it relies on the nature of the trial, protocol requirements, and outcome measures. Specifically, the efficacy response profile is the primary goal in nonrandomized trials, and the confirmation of efficacy by PR or CR is mandatory to confirm which is the best overall efficacy response.

- - 1. Time point reaction

It is assumed that an efficacy response will occur at the specific time point of each regimen. Table 1 will provide a summary of the overall efficacy response of a patient population with measurable disease at the baseline level, and at each time point.

**Table 1 Time point response: Subjects with target lesions (including or excluding non-target lesions)**

| Target focus | Non-target lesions | New lesions | Total relief |
| --- | --- | --- | --- |
| CR | CR | mistake | CR |
| CR | Non-CR / non-PD | mistake | PR |
| CR | Can&#039;t evaluate | mistake | PR |
| PR | Non-progressive or could not be fully assessed | mistake | PR |
| SD | Non-progressive or could not be fully assessed | mistake | SD |
| Not fully evaluated | Non-progression | mistake | NE |
| PD | Any situation | Yes or no | PD |
| Any situation | PD | Yes or no | PD |
| Any situation | Any situation | yes | PD |
| CR= complete response, PR= partial response, SD= stable disease, PD= disease progression, and NE = could not be evaluated | | | |

If the patient has no measured lesions (no target lesion), the evaluation can be presented in Table 2.

**Table 2 Time point responses-Only subjects with non-target lesions**

| Non-target lesions | New lesions | Total relief |
| --- | --- | --- |
| CR | mistake | CR |
| Non-CR or non-PD | mistake | Non-CR or non-PD^a^ |
| Not fully evaluated | mistake | Can&#039;t evaluate |
| Can not be clearly defined for the PD | Yes or no | PD |
| Any situation | yes | PD |
| A: For non-target lesions, &quot;non-CR / non-PD&quot; means superior efficacy over SD. As SD is increasingly used as an endpoint to evaluating efficacy, non-CR / non-PD efficacy is developed to address conditions where no measurable lesion is not specified. | | |

- - 1. Missing missing and none-valuable instructions

If lesion imaging or measurement cannot be performed at a particular point in time, the patient will not be evaluated at that time point. If only some of the lesions can be evaluated in an evaluation, it is usually considered impossible to evaluate at that time point, unless there is evidence that the missing lesion does not affect the efficacy response evaluation at the specified time point. This situation is very likely to occur in the case of disease progression. For example, a patient with three lesions with a total of 50 mm at baseline, but then only two lesions, with a total of 80 mm, will be evaluated as disease progression, regardless of the impact of the missing lesion.

- - 1. Optimum overall response: All time points

Once all the patient data are available, the best total response can be determined.

Assessment of the best total response when the study does not require confirmation of a complete or partial efficacy response: Best efficacy response in the trial was the best response at all time points (e. g., a patient was evaluated as SD in the first cycle, PR in the second cycle, PD in the last cycle, but the best total response as PR. When the best total response is evaluated as an SD, it must meet the minimum time from the baseline level specified in the protocol. If the criterion of the shortest time is not met, even if the best overall response evaluation as SD is not approved, the best overall response of this patient will depend on the subsequent evaluation. For example, a patient was evaluated as SD in the first cycle and the second cycle as PD, but it did not meet the shortest time requirement of SD, and its best overall response was evaluated as PD. The same patient lost to follow-up after the first cycle evaluation of SD will be considered as none-valuable.

Assessment of the best total response when the study requires confirmation of complete or partial response: a complete or partial response is confirmed only if each subject meets the partial or complete response criteria specified in the trial and is specifically mentioned in the protocol at subsequent time points (usually four weeks later). In this case, the best total response is illustrated in Table 3.

**Table 3 Optimum overall response for CR and PR efficacy**

| Total remission at the first time point | Total remission at the subsequent time point | Best total relief |
| --- | --- | --- |
| CR | CR | CR |
| CR | PR | SD, PD, or PR^a^ |
| CR | SD | SD if SD lasts sufficient duration, otherwise PD |
| CR | PD | SD if SD lasts sufficient duration, otherwise PD |
| CR | NE | SD if SD lasts enough, otherwise NE |
| PR | CR | PR |
| PR | PR | PR |
| PR | SD | SD |
| PR | PD | SD if SD lasts sufficient duration, otherwise PD |
| PR | NE | SD if SD lasts enough, otherwise NE |
| NE | NE | NE |
| CR= complete response, PR= partial response, SD= stable disease, PD= disease progression, and NE = could not be evaluated.  A: If any disease actually occurs at the first time point and at a subsequent time point, the efficacy evaluation will remain PD at the later time point even if the subject meets the PR criteria at baseline (as the disease will reappear after CR). Optimal response depends on having SD within the shortest treatment interval. However, sometimes the first evaluation is CR, but the subsequent time point scan indicates that small lesions seem to appear, so the subject efficacy should be PR rather than CR at the first time point. In this case, the first CR judgment should be modified to PR, while the best reaction is PR. | | |

- - 1. Special tips for the efficacy assessment

When nodular lesions are included in the total target lesion assessment and the nodule size decreases to a "normal" size (<10 mm), they will still have a lesion size scan report. To avoid excessive assessment based on the increased nodule size, the measurements will be recorded even if the nodule is normal. As already mentioned, this means that subjects with complete response will not be recorded as 0 on the CRF table.

During the trial. The analysis plan of the trial must state that these missing data / assessments can be explained clearly when determining efficacy. For example, in most trials, the response of a subject PR-NE-PR can be confirmed as the efficacy.

It should be reported as symptomatic progression when the subject experienced an overall deterioration of his or her health, but with no objective evidence. Objective progression should be assessed even after treatment termination. Symptomatic deterioration is not an objective assessment of assessment: it is the reason for discontinuing treatment. The objective response of such subjects will be assessed by the target and non-target lesion conditions shown in Tables 1 to 3.

Defined as early progression, early death, and none-valuable conditions are study exceptions and should be clearly described in each protocol (depending on the treatment interval and treatment cycle).

In some cases, it is difficult to identify local lesions from normal tissue. When evaluation of complete response is based on such a definition, biopsy is recommended before efficacy evaluation of local focal complete response. When abnormal focal imaging findings in some subjects are considered to represent focal fibrosis or scar formation, FDG-PET is used as a similar assessment criterion as biopsy to confirm the efficacy of complete response. In such cases, the application of FDG-PET should be prospectively described in the protocol, while supported by reports of the specialist medical literature for this situation. However, it must be realized that the limitation of FDG-PET and biopsy itself (including their resolution and sensitivity) will lead to false positive results in complete remission evaluation.

Treatment with ambiguous progressive findings (e. g. very small uncertain new lesions; preexisting cystic or necrotic lesions) can be continued until the next evaluation. If disease progression is confirmed in the next assessment, the date of progression should be the date of prior suspected progression.

- 1. Frequency of the tumor reevaluation

The frequency of tumor reevaluation during treatment is determined by the treatment regimen and should be consistent with the type and schedule of treatment. However, in phase II trials where the benefit of treatment is unclear, follow-up every 6 to 8 weeks (designed at the end point of a cycle) is reasonable, and the length of the interval can be adjusted under special protocol or circumstances. The protocol should specify which tissue sites require baseline evaluation (usually those most likely to be closely associated to the metastatic lesion of the tumor type studied) and the frequency of evaluation repeats. Normally, target lesions and non-target lesions should be evaluated at each evaluation. In some optional cases, some non-target lesions can be evaluated less frequently. For example, if the efficacy evaluation of the target disease is confirmed as CR or the bone scan is suspected.

After completion of treatment, the tumor re-evaluation depends on taking the response rate or the time to an event (progression / death) as the clinical trial endpoint. Time for an event (e. g. TTP / DFS / PFS) requires the routine repeat evaluation specified in the protocol. In particular, in randomized comparative trials, scheduled evaluations should be included in the schedule (e. g., 6 to 8 weeks during treatment, or 3 to 4 months after treatment) and should not be affected by other factors, such as treatment delay, dosing interval, and any other events that may lead to unbalanced treatment arm in the choice of disease evaluation time.

- 1. Efficacy assessment / confirmation of the remission period
     1. affirm

For non-randomized clinical studies with efficacy as the primary study endpoint, the efficacy of PR and CR must be confirmed to ensure that efficacy is not the result of misevaluation. This also allows for a reasonable interpretation of the results where historical data are available, but the efficacy in the historical data of these trials should also have been confirmed. However, in all other cases, such as randomized trials (phase II or III) or studies with disease stabilization or disease progression as the primary endpoint, efficacy confirmation is no longer required because this is of no value in the interpretation of trial results. Removing the requirement for efficacy confirmation, however, makes the central review even more important, especially in unblinded experimental studies.

In the case of SD, at least one measurement within the shortest time interval after the start of the trial (generally not less than 6 to 8 weeks) will meet the SD criteria specified in the protocol.

- - 1. Total remission period

The total response period was from the time of measuring the first CR or PR (which was measured first) to the time of the first true record of disease recurrence or progression (using the minimum measurement recorded in the trial as a reference for disease progression). Total time to total response was from time to first meeting CR criteria to time to first true recording of disease relapse or progression.

- - 1. Stable period of disease

Is the time from the start of treatment to disease progression (in randomized trials, from the time of randomization), with the smallest sum in the trial as a reference (if the sum of baseline is the minimum, as the reference for PD calculation). The clinical relevance of disease stabilization varies between studies and different diseases. If in the particular trial, the proportion of patients maintaining the shortest time stability period is used as the study endpoint, the protocol should specifically state the shortest time interval between the two measurements in the SD definition.

Note: Response, stabilization, and PFS were affected by the frequency of follow-up after baseline evaluation. Defining the standard follow-up frequency is not within the scope of this guideline. The frequency of follow-up should consider many factors, such as disease type and stage, treatment cycle and standard norms. However, if inter-trial comparisons are required, the endpoint accuracy limitations of these measurements should be considered.

- 1. PFS/TTP
     1. A Phase II clinical trial

This guideline focuses on the use of objective remission as a study endpoint in phase II clinical trials. In some cases, remission rates may not be optimal to evaluate the potential anticancer activity of new drugs / novel regimen. In these cases, PFS / PPF at the demarcation time points can be considered a suitable surrogate indicator of the original signal that provides the biological activity of new drugs. But it is clear that in an uncontrolled trial, these evaluations will be questioned, because seemingly valuable observations may be related to biological factors such as patient screening, rather than the role of pharmacological interventions. Therefore, phase II clinical trials with these as study endpoints should better be designed as randomized controls. But the clinical manifestations of some tumors is consistent (usually always poor) and non-randomized trials are reasonable. However, in these cases, the evidence of efficacy should be carefully documented when assessing the expected PFS or PPF due to the lack of positive controls.

**Annex IV Classification criteria for drug AE (NCI-CTC AE 5.0)**

| grade | Clinical description of the severity level |
| --- | --- |
| 1 | Mild; no clinical or mild clinical symptoms; only clinical or laboratory abnormalities; no treatment required |
| 2 | Moderate; requiring small, local or non-invasive treatment; age-appropriate limited activities of daily living (Activities of Daily Living, ADL), tool use means cooking, shopping, phone calls, counting, etc |
| 3 | Severe or medically severe symptoms but not temporarily life threatening; hospitalization or prolonged hospitalization; disability; restricted daily self-care (Self care ADL). Daily life self-care refers to: bathing, dressing, stripping, eating, going to the bathroom, taking medicine, etc., non-bedridden |
| 4 | Life-threatening and requires emergency treatment |
| 5 | Death related to the adverse events |

**Annex V SAE reporting Method**

| destination | | Reporting method |
| --- | --- | --- |
| Ethics Committee | | Submit in person |
| Support for this project | Jiangsu Hengrui Clinical Research and Development Department, Drug Safety Department | Send the signature scan copy and the word version to the email address: hengrui_drug_safety@hrglobe.cn |
| State drug administration | Drug Research Division, Department of Drug Registration, National Medical Products Administration | Email: yjjdc@nmpa.gov.cn (preferred)  Fax: 010-88363228  EMS Address: Building 2, No.26, Xuanwumen West Street, Xicheng District, Beijing (Postcode: 100053) Tel: 010-88330732 |
| The state health administration department | Medical Administration, Medical Management Authority, National Health Commission | Email address: saefax@163.com |
| Drug regulatory departments of provinces, autonomous regions and municipalities directly under the Central Government | | Each region sends it according to the requirements of the department |

**Annex VI Standard Guidelines for surgical sampling after neoadjuvant therapy**

**Determine the lesion location (tumor or tumor bed)**

Sample classification

• Samples with a pre-specified location are the samples obtained at the obvious calibration of the lesion location with a skin tattoo or metal clips before neoadjuvant therapy.

Samples without a prespecified location are samples obtained at pre-specified but unqualified lesion locations or without any localization markers, such as:

a) Samples with pre-specified but substandard localization were those obtained at lesions not clearly localized before neoadjuvant therapy but localized prior to surgery.

b) Samples without any localization markers were those obtained at lesions where localization was not clearly defined either before neoadjuvant therapy or before surgery.

The following table will provide reference for the pathology to determine the location of the lesion.

|  | (Modified) radical radical surgery | Breast preservation |
| --- | --- | --- |
| Pre-positioning  • Tattooing of skin  • Metal clips or other | Sign the corresponding area | Identified according to the surgical markers |
| Not pre-positioned:  • Localization was performed only before surgery  • No positioning | 1. Identify the quadrants according to the obvious anatomical markers on the sample;  2. Find the quadrant of the localized mass recorded at clinical diagnosis;  3. Quadrant identification | Identified according to the surgical markers |

Surgical markers

• Breast preservation is used

• Methods: Use markers in two different spots (example below; located by clinicians and pathologists on a specific basis)

graph 1 Example of the surgical markers


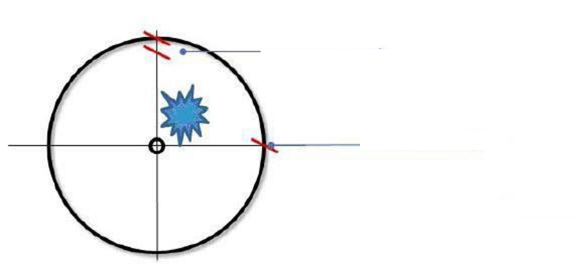


Determine lesion size (tumor or bed) Carefully identify lesions and determine tumor or bed size with three diameters:

• Pre-located samples: Carefully identify the lesion under the marker and determine the tumor or tumor bed size in three diameters.

• Pre-specified but unqualified positioning samples: carefully identify the lesions in the corresponding quadrant (see "surgical markers" above, the same below), and determine the size of the tumor or tumor bed in three different diameters.

• Samples without any localization markers: carefully identify the lesion in the quadrant and determine the tumor or bed size in three diameters.

**Tumor samples were excised**

mammary tissue

• Pre-located samples: Samples obtained from breast tissue corresponding to predefined markers (e. g., skin tattoos), with an cut every 1 cm along the long axis of breast tissue, cut into several block tissues. At least one sample was obtained from each tumor block. For example, at least 5 samples were obtained from a 5-cm large tissue.

• Samples with pre-specified but nonqualified positioning and samples without any localization markers: Samples should be carefully obtained from the quadrants corresponding to the primary tumor mass

1. Samples with significant tumor mass: Make an incision every 1cm along the long axis of the sample, and cut the sample into several massive tissues. At least one sample should be obtained from each tumor block. For example, at least 5 samples were obtained from a 5-cm large tissue.

A) If there is a microscopic, non-pathological complete response, additional sampling is not required

And b) obtain samples from all quadrants if microscopic pCR (from all breast tissue)

2. Samples with nonsignificant tumor mass but fibrobed: Samples were obtained from the bed using the above method.

A) For microscopic non-pCR, no additional sampling is necessary

And b) obtain samples from all quadrants if microscopic pCR (from all breast tissue)

3. Samples with no significant tumor mass or tumor bed: from all areas under the marker (for breast conserving, from all breast tissue)

lymph gland

• The lymph nodes of the axillary adipose tissue were strictly examined.

• Sections were along the long axis of the lymph nodes.

The largest section was submitted for the histological evaluation of the adjacent connective tissue. All identified axillary lymph nodes should be delivered for histological examination. More detailed description of the sentinel lymph node biopsy is provided in each guideline document. This sampling guideline provides standard post-operative sampling methods only after neoadjuvant therapy.
